# Bioactive Cyclopeptide Alkaloids and Ceanothane Triterpenoids from *Ziziphus mauritiana* Roots: Antiplasmodial Activity, UHPLC-MS/MS Molecular Networking, ADMET Profiling, and Target Prediction

**DOI:** 10.3390/molecules30142958

**Published:** 2025-07-14

**Authors:** Sylvestre Saidou Tsila, Mc Jesus Kinyok, Joseph Eric Mbasso Tameko, Bel Youssouf G. Mountessou, Kevine Johanne Jumeta Dongmo, Jean Koffi Garba, Noella Molisa Efange, Lawrence Ayong, Yannick Stéphane Fotsing Fongang, Jean Jules Kezetas Bankeu, Norbert Sewald, Bruno Ndjakou Lenta

**Affiliations:** 1 Department of Organic Chemistry, Faculty of Science, University of Yaoundé I, Yaoundé P.O. Box 812, Cameroon; saidoutsila@yahoo.fr (S.S.T.); jumetakevine@yahoo.fr (K.J.J.D.); 2 Department of Chemistry, Higher Teacher Training College, University of Yaoundé I, Yaoundé P.O. Box 47, Cameroon; mcjesuskinyok@yahoo.fr (M.J.K.); tamekombasso@yahoo.fr (J.E.M.T.); mountessou@yahoo.com (B.Y.G.M.); bk_jeanjules@yahoo.fr (J.J.K.B.); 3 Department of Basic Science Education, National Advanced School of Maritime and Ocean Science and Technology, University of Ebolowa, Kribi P.O. Box 292, Cameroon; garbakoffijean@yahoo.fr; 4 Department of Biochemistry and Molecular Biology, Faculty of Science, The University of Buea, Buea P.O. Box 63, Cameroon; noella.efange@yahoo.com; 5 Malaria Research Service, Centre Pasteur du Cameroun, Yaoundé P.O. Box 1274, Cameroon; ayong@pasteur-yaounde.org; 6 Department of Chemistry, Higher Teachers’ Training College, The University of Maroua, Maroua P.O. Box 55, Cameroon; 7 Organic and Bioorganic Chemistry, Faculty of Chemistry, Bielefeld University, D-33501 Bielefeld, Germany; norbert.sewald@uni-bielefeld.de

**Keywords:** *Ziziphus mauritiana*, cyclopeptide alkaloids, ceanothane triterpenoids, antiplasmodial activity, in silico pharmacology, ADMET profiling

## Abstract

Malaria continues to pose a significant global health burden, driving the search for novel antimalarial agents to address emerging drug resistance. This study evaluated the antiplasmodial potential of *Ziziphus mauritiana* Lam. (Rhamnaceae) roots through an integrated phytochemical and pharmacological approach. The ethanol extract, along with its derived fractions, demonstrated potent in vitro activity against the *chloroquine-sensitive Plasmodium falciparum* strain 3D7 (*Pf*3D7), with the ethyl acetate-soluble (IC_50_ = 11.35 µg/mL) and alkaloid-rich (IC_50_ = 4.75 µg/mL) fractions showing particularly strong inhibition. UHPLC-DAD-ESI-QTOF-MS/MS-based molecular networking enabled the identification of thirty-two secondary metabolites (**1**–**32**), comprising twenty-five cyclopeptide alkaloids (CPAs), five of which had not yet been described (**11**, **20**, **22**, **23**, **25**), and seven known triterpenoids. Bioactivity-guided isolation yielded thirteen purified compounds (**5**, **6**, **14**, **26**–**30**, **32**–**36**), with betulinic acid (**30**; IC_50_ = 19.0 µM) and zizyberenalic acid (**32**; IC_50_ = 20.45 µM) exhibiting the most potent antiplasmodial effects. Computational ADMET analysis identified mauritine F (**4**), hemisine A (**10**), and nummularine R (**21**) as particularly promising lead compounds, demonstrating favourable pharmacokinetic properties, low toxicity profiles, and predicted activity against both family A G protein-coupled receptors and evolutionarily distinct Plasmodium protein kinases. Quantitative analysis revealed exceptionally high concentrations of key bioactive constituents, notably zizyberenalic acid (24.3 mg/g) in the root extracts. These findings provide robust scientific validation for the traditional use of Z. mauritiana in malaria treatment while identifying specific cyclopeptide alkaloids and triterpenoids as valuable scaffolds for antimalarial drug development. The study highlights the effectiveness of combining advanced metabolomics, bioassay-guided fractionation, and computational pharmacology in natural product-based drug discovery against resistant malaria strains.

## 1. Introduction

Malaria remains a life-threatening disease endemic to tropical and subtropical regions, with Africa bearing the highest burden. Despite available treatments, drug resistance poses a significant challenge to malaria control. In 2024, approximately 263 million malaria cases resulted in nearly 597,000 deaths worldwide, with 95% of cases and 76% of fatalities occurring in Africa, primarily among children under five [1]. Artemisinin-based combination therapies (ACTs) are currently the standard for malaria treatment in Africa, but resistance has emerged in Asia and is now being reported in Africa [2,3]. The search for new antimalarial leads remains crucial in combatting drug-resistant malaria. Notably, all major antimalarial drugs, including quinine and artemisinin, are plant-derived. This study focuses on *Ziziphus mauritiana* (Rhamnaceae), a medicinal plant used in folk medicine to treat malaria and other ailments [4,5]. *Z. mauritiana* is a shrub or small tree reaching up to 15 m in height that is widely distributed in tropical and subtropical zones [6]. Its fruits are consumed fresh or dried and are processed into various food products [7], while folk medicine employs the plant to treat diverse conditions, including malaria, diarrhea, tumors, cough, ulcers, inflammation, asthma, syphilis, gonorrhea, wounds, fever, and psychiatric disorders [4,5].

Previous chemical investigations of *Z. mauritiana* led to the isolation of cyclopeptide alkaloids (CPAs), ceanothane-type triterpenoids, saponins, lignans, and flavonoids, with CPAs and ceanothane-type triterpenoids as chemical markers [6,8]. The chemical study of the leaves and fruits principally revealed the presence of phenolic derivatives, while ceanothic acid and belunic acid were reported as the major secondary metabolites from the roots and stem bark, respectively [9]. Previous chemical studies on the leaves and stem bark of *Z. mauritiana* also involved the use of LC-MS/MS metabolomic profiling, leading to the identification of several classes of secondary metabolites including flavonoids, alkaloids, and triterpenoids showing a similar chemical composition to flavonoid derivatives as principal constituents [10,11,12]. In addition, the LC-MS/MS metabolomic profiling of fruits and seeds also presented several classes of compounds such as flavonoids, alkaloids, terpenoids, and organic acid [12,13,14]. Despite recent work on *Z. mauritiana*, no LC-MS/MS metabolomic profiling has been reported on its roots. A previous biological study presented *Z. mauritiana* as a plant possessing various biological activities including antiplasmodial, antimicrobial, and cytotoxic effects. The roots are reported to possess antiplasmodial and antimycobacterial activities and a cytotoxicity effect [15,16]. The biological study of the bark and leaves showed their anti-oxidant activity and α-amylase inhibitory activity. Likewise, the fruit pulps and seeds are reported to possess antioxidant, anti-inflammatory and antibacterial potential [12,17]. Modern drug discovery increasingly incorporates computational approaches for metabolite identification, toxicity prediction, ADMET parameter evaluation, and target prediction. Despite previous research on *Z. mauritiana*, comprehensive metabolic profiling and in silico pharmacokinetic studies of its root constituents with antiplasmodial activity remain unexplored. As part of our ongoing search for antimalarial candidates from Cameroonian medicinal plants, we investigated *Z. mauritiana* roots and report the UHPLC-DAD-ESI-QTOF-MS metabolic profiling implemented with two approaches, in addition to conventional methods combining MS/MS data with the SNAP-MS and GNPS platform, the in vitro antiplasmodial evaluation of extracts and isolated compounds, and in silico ADMET studies.

## 2. Results

### 2.1. Antiplasmodial Activity

The antiplasmodial assay was performed in triplicate. The ethanol (EtOH) crude extract demonstrated moderate antiplasmodial activity against the chloroquine-sensitive *Plasmodium falciparum* strain, *Pf*3D7 (IC_50_ = 32.70 µg/mL), while the ethyl acetate (EtOAc) extract showed no significant activities at the tested concentrations compared to the reference drugs artemisinin (IC_50_ = 26.3 nM) and chloroquine (IC_50_ = 29.9 nM). Bioactivity-guided fractionation of the active EtOH extract yielded two potent fractions: an alkaloid-rich fraction exhibiting promising antiplasmodial activity (IC_50_ = 4.75 µg/mL) and an EtOAc-soluble fraction with moderate efficacy (IC_50_ = 11.35 µg/mL).

### 2.2. Metabolic Profiling of Z. mauritiana Roots

**Figure 1:**
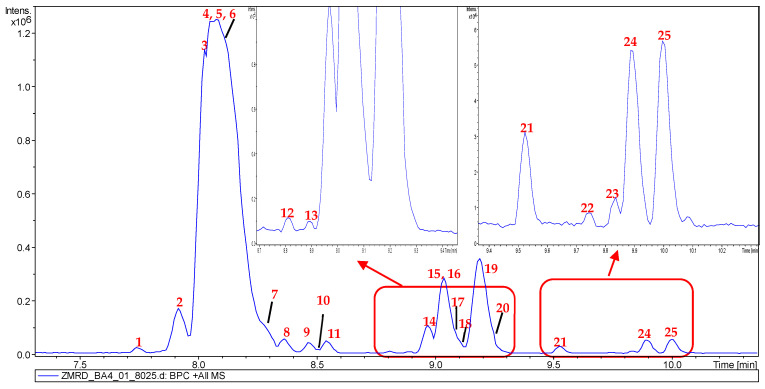
UHPLC-DAD-ESI-MS of the alkaloid-rich fraction of *Z. mauritiana*.

**Figure 2:**
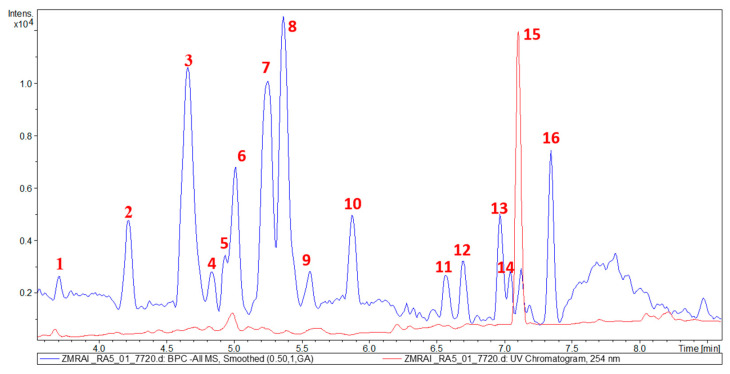
UHPLC-DAD-ESI-MS of the EtOAc soluble fraction of *Z. mauritiana*.

Comprehensive metabolic characterization of the bioactive fractions was conducted using UHPLC-MS/MS analysis. The alkaloid-rich fraction exhibited a relatively simplified chemical profile, with approximately twenty major chromatographic peaks detectable in the base peak chromatogram (Figure 1). In contrast, the ethyl acetate (EtOAc)-soluble fraction demonstrated greater chemical complexity, containing approximately sixteen major peaks in its chromatographic profile (Figure 2).

Three complementary mass spectrometry-based approaches were employed for comprehensive metabolite annotation: the Similarity Network Annotation Platform for Mass Spectrometry (SNAP-MS) molecular networking, GNPS2 Feature-Based Molecular Networking (FBMN) with ChemWalker integration, and comparative analysis with published MS/MS data.

**Figure 3:**
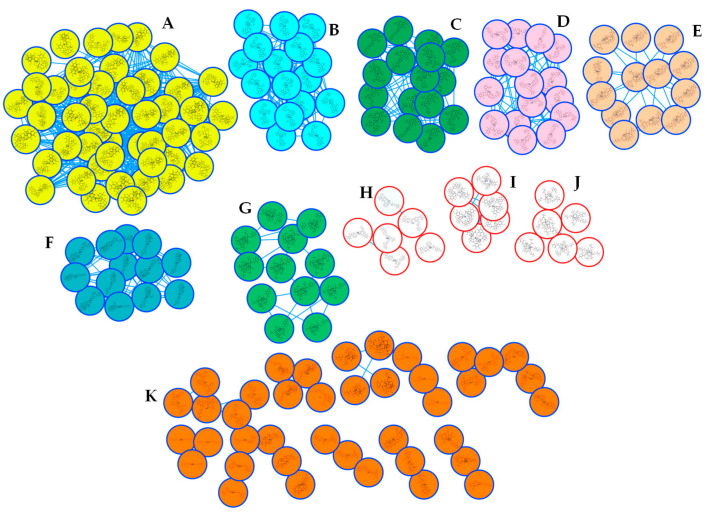
SNAP MS molecular networking from the alkaloid-rich fraction. (**A**): CPA derivatives, (**B**): indoloquinoline alkaloid derivatives, (**C**): benzo[a]heptalene alkaloid derivatives, (**D**): indole alkaloid derivatives, (**E**): steroid derivatives, (**F**): steroid peptide derivatives, (**G**): derivatives of lycotonine-type diterpenoid alkaloids, (**H**): piperidine and piperazine derivatives, (**I**): derivatives of cyclic pentapeptide alkaloids, (**J**): diterpenoids, (**K**): triterpenoids.

**Figure 4:**
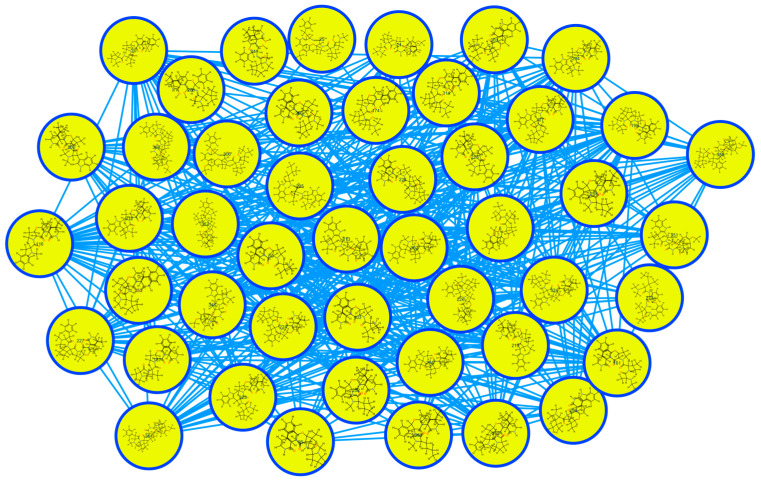
SNAP-MS Subnetwork A from the alkaloid-rich fraction.

The SNAP-MS molecular networking analysis (Figure 3), utilizing the COCONUT database, revealed 11 distinct molecular subnetworks corresponding to different phytochemical classes. Network construction was based on structural similarity fingerprints and filtered by plant genus specificity. Notably, subnetwork A (Figure 4) was predominantly composed of cyclopeptide alkaloids (CPAs), as evidenced by characteristic fragmentation patterns and precursor masses. Network visualization and compound annotation were performed using Cytoscape 3.10.1, with node–edge relationships representing mass spectral similarities (cosine score > 0.7).

**Figure 5:**
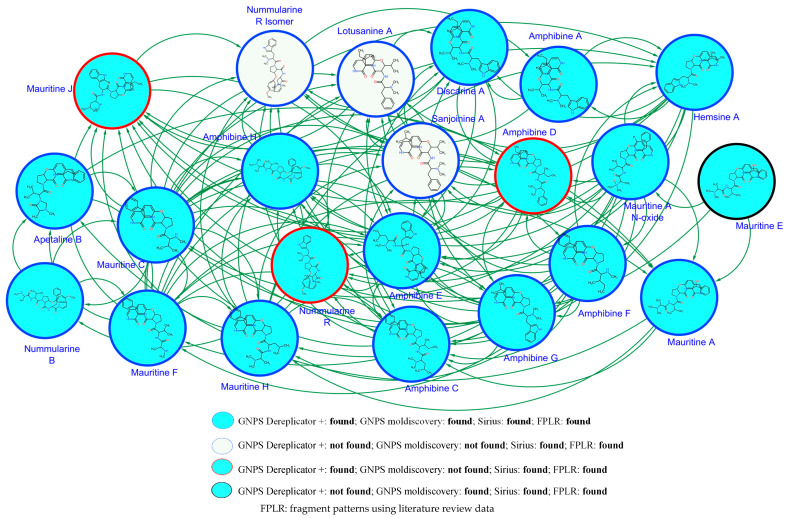
SNAP-MS Subnetwork A from the alkaloid-rich fraction after filtering.

Compounds within subnetwork A (Figure 4) were further characterized using an integrated analytical approach. Initial annotation was performed using GNPS Dereplicator+ and GNPS MolDiscovery, followed by in silico fragmentation analysis with SIRIUS software 5.8.6. Structural assignments were confirmed by comparing experimental MS/MS spectra with the literature-reported fragmentation patterns of known cyclopeptide alkaloids [18,19,20,21]. The filtered molecular network, presented in Figure 5, revealed distinct clusters of structurally related metabolites. The final annotated compounds, including their retention times, accurate masses, and spectral matches, are summarized in Table 1. This multi-platform strategy enabled the confident identification of both known and potentially novel cyclopeptide alkaloids in the active fractions. Key structural features identified included characteristic cyclopeptide alkaloid backbones, consistent with prior reports from *Ziziphus* species, as well as modified derivatives exhibiting close spectral similarity to reference compounds. The integration of computational tools with literature data provided robust evidence for the presence of these bioactive constituents, supporting their potential role in the observed antiplasmodial effects.

**Figure 6:**
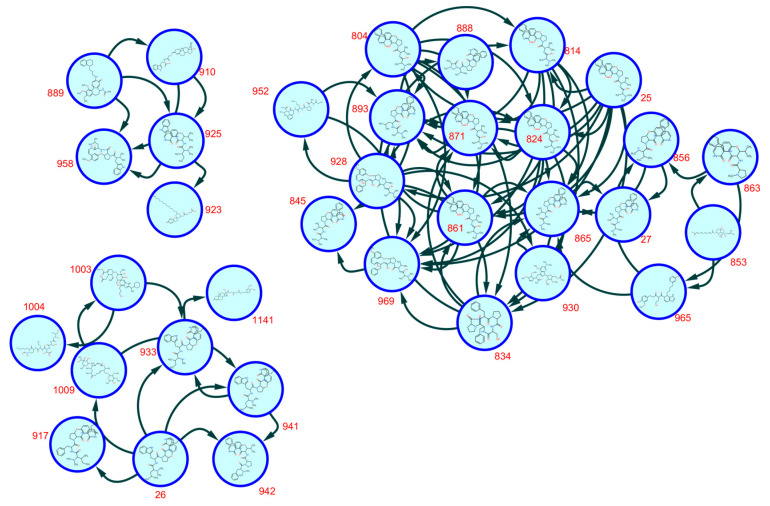
Feature-based molecular networking of the alkaloid-rich fraction annotated with GNPS2 ChemWalker tools.

Analysis of the alkaloid-rich fraction through Feature-Based Molecular Networking (FBMN) did not yield any matches in the MS/MS spectral library. However, structural annotation was achieved using the GNPS2 ChemWalker workflow (Figure 6), which enabled the tentative identification of compounds based on their fragmentation patterns and spectral similarity. The annotated compounds in the alkaloid-rich fraction and the EtOAc soluble fraction are compiled in Table 1 and Table 2, respectively.

From the alkaloid-rich fraction, 25 compounds in total were identified, predominantly belonging to the cyclopeptide alkaloid class (compounds **1**–**10**, **12**–**19**, **21**, and **24**). Additionally, the LC-MS/MS analysis of the EtOAc-soluble fraction led to the identification of seven triterpenoids (**26**–**32**) (Table 2). Notably, compounds **1**, **6** and **13** were not detected by GNPS Dereplicator+, while **6**, **12**, **13**, **17** and **21** were not annotated via GNPS MolDiscovery. Furthermore, compounds **11**, **20**, **22**, **23**, and **25** remained unidentified by SNAP MS, GNPS Dereplicator+, SIRIUS, or literature comparisons, necessitating structural proposals based solely on their MS/MS fragmentation patterns. This multi-strategy approach facilitated the comprehensive annotation of both known and potentially novel metabolites, reinforcing the chemical diversity within the bioactive fractions of *Z. mauritiana* roots.

### 2.3. Structure Elucidation of CPAs Using MS/MS Patterns

**Figure 7:**
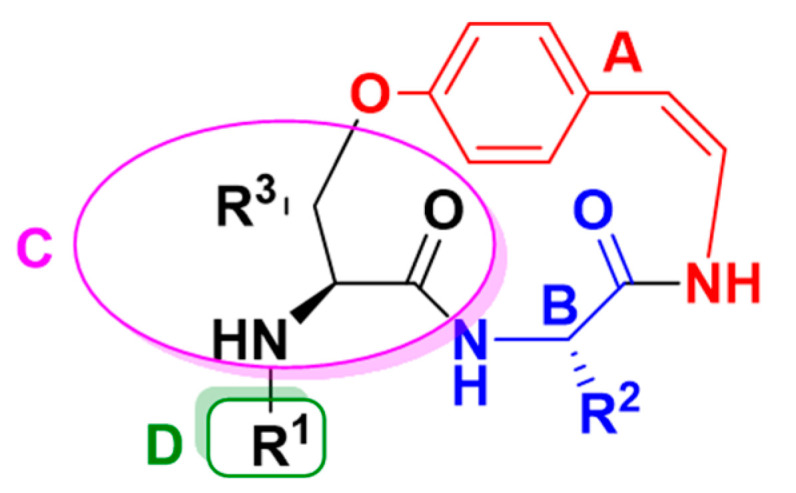
Cyclopeptide alkaloids units.

**Figure 8:**
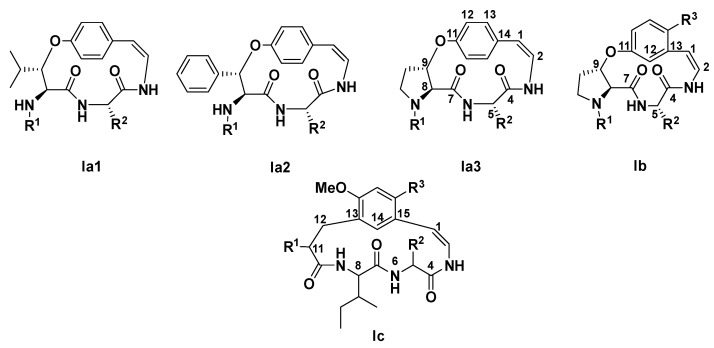
Cyclopeptide alkaloids of type I core.

Compound identification follows established metabolomics standards, with LC-MS/MS analyses corresponding to confidence level 1 [22]. Cyclopeptide alkaloids (CPAs) are classified into eight structural types and their structures can be subdivided into four main parts: hydroxy-styrylamine unit (A), ring-bound amino acid (B), *β*-hydroxy-amino acid, basic terminal (end) amino acid (D). Those from *Z. mauritiana* exclusively belong to type I [13,20]. This type comprises six subclasses (Ia1–Ia4, Ib, and Ic) (Figure 7 and Figure 8), distinguished by characteristic structural features (Table 3).

The putative structures of compounds **11**, **20**, **22**, **23** and **25** were proposed based on their MS/MS fragmentation patterns and a comparison with identified CPA analogues, as no literature reports currently exist for these metabolites. Fragmentation pathways consistent with known CPAs [15,18,19,20] provided critical evidence for structural assignments (Supporting Information). Nevertheless, NMR data would be needed for complete characterization if the putative compounds were isolated.

**Figure 9:**
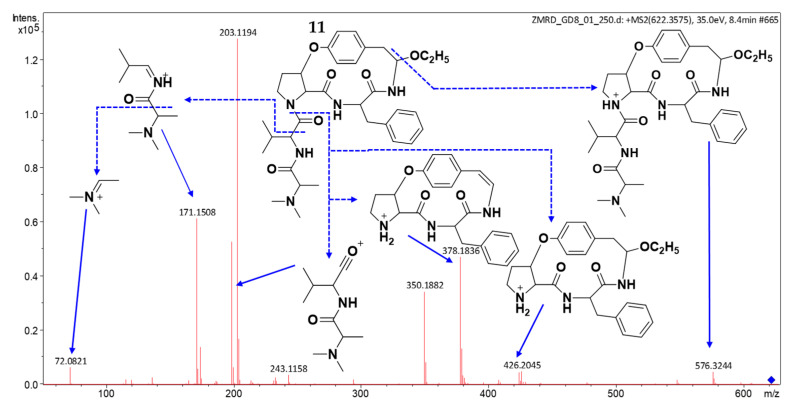
(+) ESI MS/MS spectrum of **11**.

The molecular formula, C_34_H_47_N_5_O_6_, was found for compound **11** (t_R_: 8.58 min) based on the (+) HRESIMS spectrum, which showed the protonated molecular ion peak [M + H]^+^ at *m*/*z* 622.3603 (calcd for C_34_H_48_N_5_O_6_^+^, 622.3599). MS/MS fragmentation (Figure 9) exhibited close similarity to that of mauritine A (**5**) and 1-hydro-2*β*-methoxymauritine A (**6**) (Figures S11 and S13), with diagnostic fragments at *m*/*z* 378 indicating a type Ia3 CPA core featuring phenylalanine at either the R^2^ side chain or C-position (Figure 8) [20,21,23]. Key supporting fragments included *m*/*z* 199 and 171 (valine and *N*,*N*-dimethylalanine residues), *m*/*z* 72 (confirming *N*,*N*-dimethylalanine in unit A), and *m*/*z* 576/424 (suggesting an ethoxy-modified Ia3 core). These collective data identify compound **11** as a putative novel cyclopeptide alkaloid derivative.

**Figure 10:**
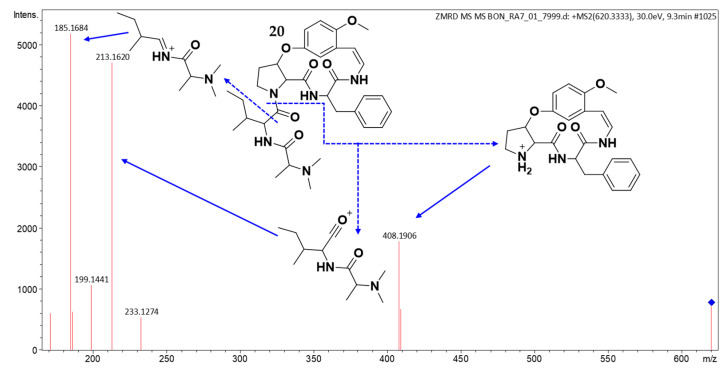
(+) ESI MS/MS spectrum of **20**.

Compound **20** showed a protonated molecular ion peak [M + H]^+^ at *m*/*z* 620.3446 in (+)-HRESIMS, corresponding to the molecular formula C_34_H_45_N_5_O_6_ (calcd for C_34_H_46_N_5_O_6_^+^, 620.3443, Δ 0.5 ppm). The MS/MS spectrum (Figure 10) contained a characteristic fragment at *m*/*z* 408, indicating either a type Ib CPA core with a benzyl residue as R^2^ (phenylalanine) and methoxy as R^3^, or 4(13)-nummularine C/5(13)-zizyphine A-type structures with phenylalanine at unit C [20,21,23]. Additional fragments at *m*/*z* 213, 199, 185, and 171 revealed terminal leucine/isoleucine and *N*,*N*-dimethylalanine residues. This evidence collectively identifies compound **20** as a putative previously unreported cyclopeptide alkaloid.

**Figure 11:**
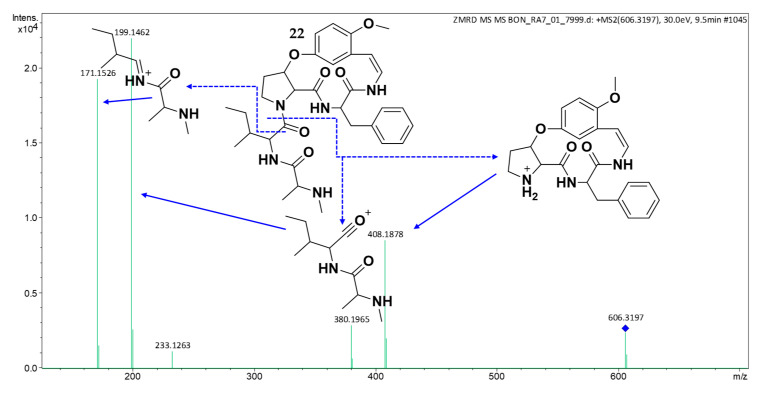
(+) HRESIMS/MS of compound **22**.

HRESIMS analysis of compound **22** (t_R_: 9.74 min) established the molecular formula, C_33_H_41_N_5_O_5_, and {[M + H]^+^ observed *m*/*z* 606.3278, calcd for C_33_H_42_N_5_O_5_^+^, 606.3286; Δ 1.3 ppm}. The MS/MS spectrum (Figure 11) exhibited a diagnostic fragment at *m*/*z* 408, consistent with either (1) a type Ib CPA core featuring phenylalanine at R^2^ with methoxy substitution at R^3^, or (2) 4(13)-nummularine C/5(13)-zizyphine A-type structures containing phenylalanine at unit C [20,21,23]. Key fragments at *m*/*z* 199 and 171 indicated terminal amino acids comprising isoleucine/leucine and *N*-methylalanine. The close similarity of the MS/MS pattern to amphibine H (**16**) suggested that compound **22** represents a structural isomer. These findings identify **22** as a putatively novel cyclopeptide alkaloid derivative.

**Figure 12:**
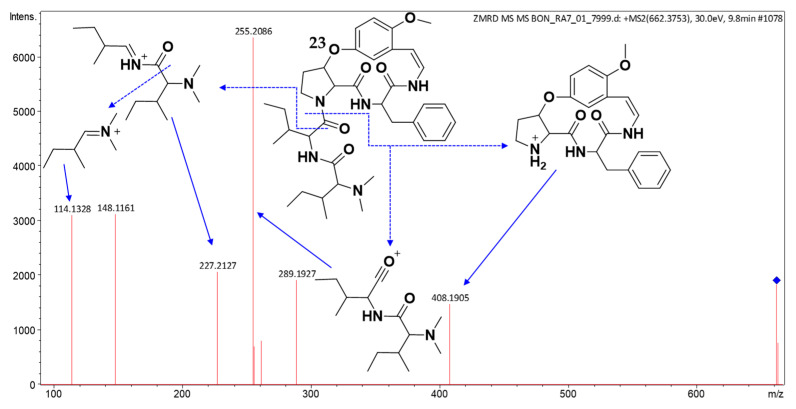
(+)-ESI MS/MS spectrum of **23**.

Compound **23** (*t*_R_: 9.82 min) was shown to have the molecular formula C_37_H_51_N_5_O_6_, by (+)-HRESIMS analysis, which showed the protonated molecular ion peak at *m*/*z* 662.3902 (calcd for C_37_H_52_N_5_O_6_^+^, 662.3912; Δ 1.5 ppm). The MS/MS spectrum (Figure 12) exhibited a characteristic fragment at *m*/*z* 408, consistent with either a type Ib cyclopeptide alkaloid core featuring phenylalanine at the R^2^ position with a methoxy substitution at R^3^, or, alternatively, 4(13)-nummularine C/5(13)-zizyphine A-type structures containing phenylalanine at unit C [20,21,23]. Additional diagnostic fragments at *m*/*z* 255 and 227 indicated the presence of two isoleucine or leucine groups, while the fragment at *m*/*z* 114 further confirmed these amino acid residues. These spectral data support the identification of compound **23** as a putative previously undescribed cyclopeptide alkaloid.

**Figure 13:**
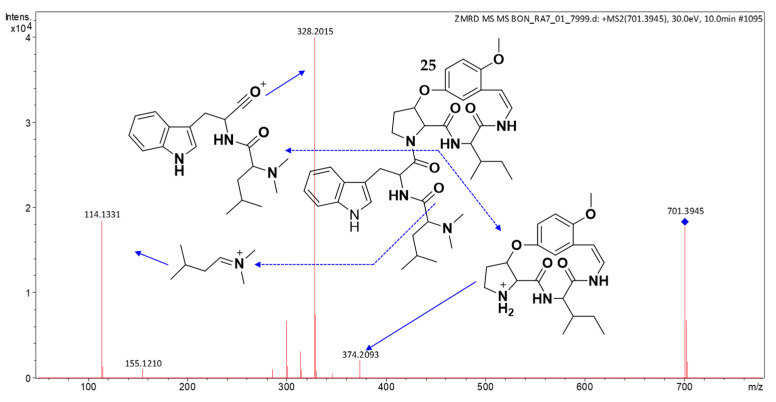
(+)-ESI MS/MS spectrum of **25**.

HRESIMS analysis of compound **25** (*t*_R_: 10.00 min) established the molecular formula, C_39_H_52_N_6_O_6_, and {[M + H]^+^ observed *m*/*z* 701.4023, calcd for C_39_H_53_N_6_O_6_^+^, 701.4021; Δ0.3 ppm}. The MS/MS spectrum (Figure 13) revealed a diagnostic fragment at *m*/*z* 374, characteristic of a type Ib CPA core with leucine/isoleucine at R^2^/unit C and methoxy substitution on the styrylamine unit D, or alternatively 4(13)-nummularine C/5(13)-zizyphine A-type structures [20,21,23]. The base peak at *m*/*z* 328 indicated the presence of tryptophan and *N*,*N*-dimethylleucine/isoleucine residues (R^1^), while *m*/*z* 114 confirmed *N*,*N*-dimethylleucine/isoleucine at unit A. A comparative analysis with mauritine M (**24**) (Figure S51) revealed an additional methyl group in compound **25**. These comprehensive spectral data support the identification of **25** as a putative novel cyclopeptide alkaloid derivative.

The structural elucidation was further reinforced by the distinct fragmentation pattern, which aligns with established cyclopeptide alkaloid frameworks while exhibiting unique features that distinguish it from known analogues. The mass accuracy of the molecular ion and the consistency of the fragment ions with proposed structural features provide compelling evidence for this novel assignment. The combination of high-resolution mass spectrometry and tandem MS fragmentation analysis enabled the confident characterization of these putative new natural products.

In view of their interesting chemical composition, the active fractions were further subjected to different chromatography methods.

### 2.4. Isolation and Characterization of Compounds

**Figure 14:**
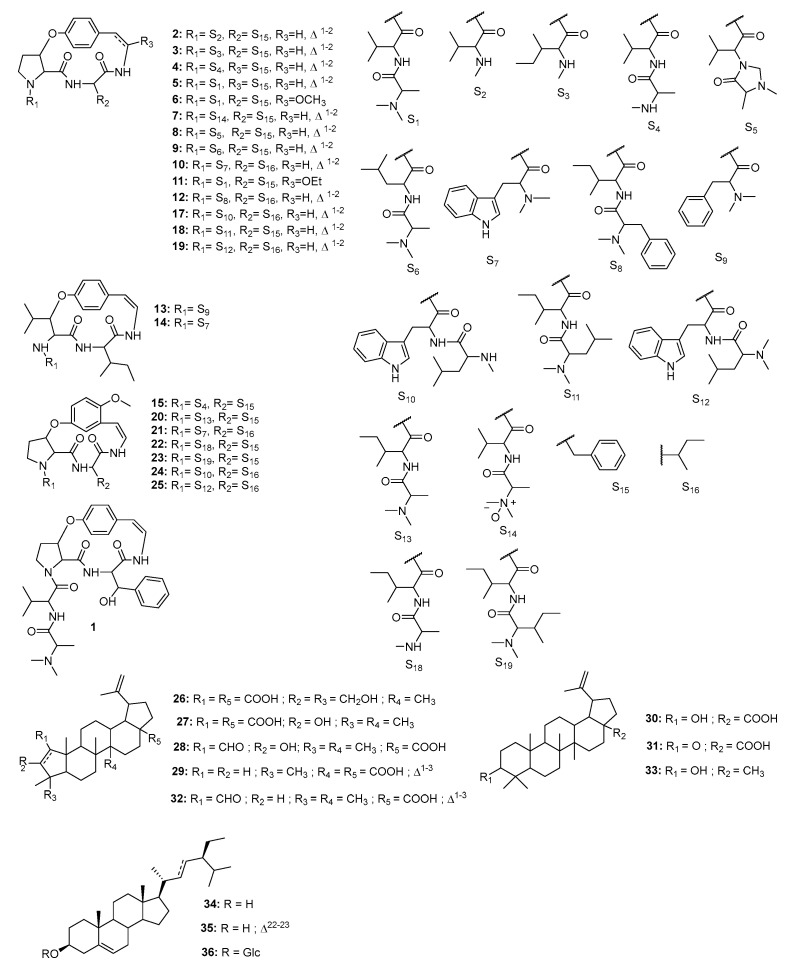
Chemical structures of putative/identified and isolated compounds (**1**–**36**) from *Ziziphus mauritiana* roots.

Given their promising antiplasmodial activity and complex chemical composition, the alkaloid and ethyl acetate-soluble fractions were subjected to extensive chromatographic purification. This led to the isolation and characterization of thirteen compounds, comprising seven triterpenoids {24-hydroxyceanothic acid (**26**) [9], ceanothic acid (**27**) [24], zizyberanalic acid (**28**), ceanothenic acid (**29**) [25], betulinic acid (**30**) [24], zizyberenalic acid (**32**) [25], and lupeol (**33**) [9]}, three steroids {*β*-sitosterol (**34**), stigmasterol (**35**) [26], and sitosterol 3-*O*-*β*-D-glucopyranoside (**36**) [27,28]}, and three cyclopeptide alkaloids (CPAs) {mauritine A (**5**) [29], 1-hydro-2*β*-methoxymauritine A (**6**), and amphibine A (14) [28]} (Figure 14).

### 2.5. Chemophenetic Significance

Compounds **1**–**10**, **12**–**19**, **21**, **24** and **26**–**32** were previously reported from the genus *Ziziphus*, with compounds **1**–**5**, **9**, **10**, **12**, **15**, **17**, **19**, **24**, and **25**–**32** specifically identified in *Z. mauritiana* [15,20,24,30]. Notably, ceanothic acid (**27**) and betulinic acid (**30**) were found to be the major constituents in the roots of *Z. mauritiana*, consistent with previous phytochemical studies on this species [24].

### 2.6. Antiplasmodial Activities of Compounds

The ethanol extract demonstrated moderate antiplasmodial activity against the *Pf*3D7 strain with an IC_50_ value of 32.70 μg/mL, while the ethyl acetate (EtOAc) extract was inactive (IC_50_ > 50 μg/mL). Further fractionation of the active ethanol extract yielded more potent fractions, with the EtOAc-soluble fraction showing an IC_50_ value of 11.35 μg/mL and the alkaloid-rich fraction exhibiting stronger activity (IC_50_ = 4.75 μg/mL). Among the isolated compounds, betulinic acid (**30**) displayed significant antiplasmodial activity (IC_50_ = 19.0 μM). Zizyberenalic acid (**32**) also showed notable activity against the 3D7 strain (IC_50_ = 20.45 μM), consistent with previous reports on its effectiveness against the *Pf*K1 strain (IC_50_ = 6.62 μM) [31]. Additional compounds with documented antiplasmodial activity against *Pf*K1 are summarized in Table 4.

These findings indicate that the antiplasmodial properties of *Z. mauritiana* roots are primarily attributable to specific triterpenoid and alkaloid constituents. The enhanced activity observed in fractions compared to the crude extracts suggested an increase in the concentration of active ingredients, potential synergistic effects or the presence of inhibitory compounds in the whole extract. The identification of these bioactive compounds provides a foundation for a further investigation of their mechanism of action and potential development as antimalarial agents.

The increase in antiplasmodial activity observed in the fractions and isolated compounds, compared to the ethanol extract, could be explained by the low concentration of active ingredients or the antagonistic interactions among compounds in the crude extract. The lack of activity displayed by mauritine A (**5**), 1-hydro-2*β*-methoxymauritine A (**6**), and amphibine A (**14**) supports the hypothesis proposed by Tuenter et al. (2017) [32], which suggested that a 13-membered macrocyclic ring is more favorable for activity than a 14-membered ring. The ceanothane triterpenoids and CPAs may be the bioactive classes responsible for the antiplasmodial activity of this plant material.

Given the antiplasmodial potency of these compounds, it is therefore very important to further evaluate their potential toxicity, pharmacokinetic properties, drug-likeness, and plausible biological targets.

### 2.7. In Silico Acute Toxicity Prediction of Bioactive Constituents

Compounds demonstrating promising antiplasmodial activity were further subjected to in silico toxicity profiling to assess their selectivity and confirm their potential as safe antiparasitic agents. Toxicity predictions were performed using ProTox 3.0 (accessed June 2024) [33], evaluating key endpoints including hepatotoxicity, nephrotoxicity, respiratory toxicity, and cardiotoxicity (Table 5). The results indicated that compounds **4**, **10**, **12**, **21**, **24**, and **32** were classified under toxicity class 4 (LD_50_ = 386–1000 mg/kg) for acute oral toxicity, while compound **15** fell under toxicity class 3 (LD_50_ = 200 mg/kg). Most compounds (**4**, **10**, **12**, **15**, **21**, and **32**) were predicted to be devoid of hepatotoxicity, nephrotoxicity, and cardiotoxicity. However, they exhibited potential respiratory toxicity, though compounds **12**, **21**, and **32** had probabilities below the average threshold (0.78). Notably, compound **24** was predicted to be devoid of hepatotoxicity and cardiotoxicity, but showed potential nephrotoxicity and respiratory toxicity.

### 2.8. Pharmacokinetic (ADMET) Profiling, Drug-Likeness, and Molecular Target Prediction of Bioactive Compounds

**Figure 15:**
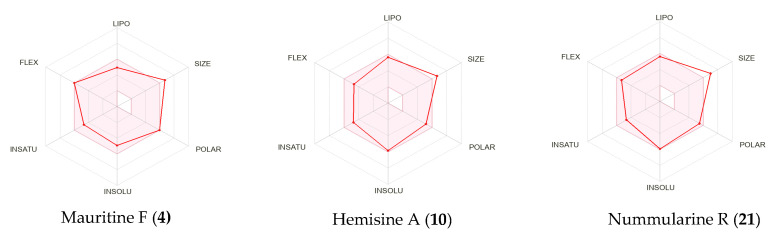
Radar chart bioavailability.Lipophilicity: XLOGP3 between −0.7 and +5.0, size: MW between 150 and 500 g/mol, polarity: TPSA between 20 and 130 Å^2^, solubility: logS not higher than −6, saturation: fraction of carbons in the *sp*^3^-hybridization not less than 0.25, flexibility: no more than nine rotatable bonds.

Compounds **4**, **10**, **12**, **15**, **21**, **24**, **32**, identified as the most promising active secondary metabolites, were subjected to the pharmacokinetic analysis, drug-likeness, and molecular target prediction using SwissADME [34]. As shown in Table 6, their physicochemical properties and lipophilicity data were generated and analyzed. Water solubility, a crucial factor in drug development, was assessed using the ESOL model, revealing that compounds **4**, **10**, **15**, and **21** possess moderate solubility with Log S values ranging from −5.94 to −4.95. The pharmacokinetic evaluation focused on several key parameters: gastrointestinal (GI) absorption, blood–brain barrier (BBB) permeability, P-glycoprotein substrate status, permeability, and cytochromes P450 (CYP) interactions. Compounds **4**, **10**, **12**, **15**, and **21** exhibited high GI absorption, indicating their potential for effective systemic distribution. Notably, none of the compounds showed BBB permeability, suggesting a minimal risk of central nervous system effects. The analysis revealed that compounds **4**, **10**, **12**, **15**, **21**, and **24** are likely substrates of P-glycoprotein, which influences their absorption, distribution, and excretion profiles. CYP450 interactions were particularly noteworthy, as these enzymes play a critical role in drug metabolism. While none of the compounds interacted with CYP1A2, CYP2C19, CYP2C9, or CYP2D6 isoforms, all showed potential interactions with CYP3A4. This interaction could lead to drug–drug interactions but might also be beneficial by potentially enhancing therapeutic efficacy through increased plasma levels of rapidly metabolized drugs. Drug-likeness was assessed using four rule-based filters (Lipinski, Ghose, Egan, Veber). The evaluation showed that compounds **4**, **10**, **12**, **21**, and **32** satisfied four of the five Lipinski’s rules and two criteria of Egan’s rules, while compounds **4**, **9**, **15**, **21**, and **32** met Veber’s criteria. None of the compounds complied with Ghose’s filter. Importantly, compounds **4**, **10**, **12**, and **21** demonstrated good bioavailability with a score of 0.55. The bioavailability radar (Figure 15) further supported these findings, showing that compounds **4**, **10**, and **21** possessed suitable physicochemical properties for oral bioavailability, with all parameters except molecular weight falling within the optimal range.

**Figure 16:**
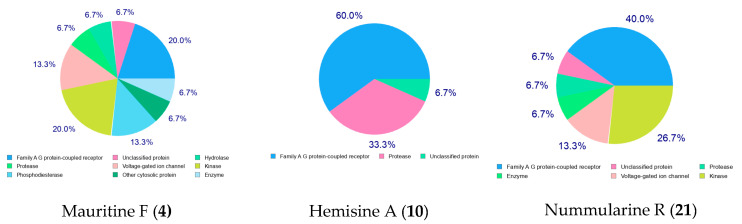
Pie chart of molecular targets.

Based on their solubility and pharmacokinetic profiles, compounds **4**, **10**, and **21** emerged as particularly promising candidates. Their predicted molecular targets, illustrated in Figure 16, revealed that family A G protein-coupled receptor represented 20%, 60%, and 40% of the targets for compounds **4**, **10**, and **21**, respectively, as well as artemisinin 6.7% and chloroquine 60% (Supporting Information Figures S84 and S85). Liu and collaborators [35] described the impact of the lateral basic amine chain of chloroquine in the specific interactions with MrgprX1, particularly via ionic and hydrophobic bonds. The presence of basic terminal (end) amino acid on compounds **4**, **10**, **21** could be a fundamental aspect able to support the prediction of this target. The interactions of functional groups such as heterocyclic and primary amines, an aromatic ring with parasitic protein kinases (*Pf*CK, *Pf*EK, *Pf*CDPK1, *Pf*PK6), have already been reported in several works, which could explain the ability of CPAs to be used as inhibitors of these proteins [36,37]. Protease enzymes accounted for 6.7%, 33.3%, and 6.7% of targets for these same compounds. Additionally, compound **21** showed potential activity against kinases (26.7%) and voltage-gated ion channels (13.3%), while compound **4** was predicted to interact with kinases (20%), voltage-gated ion channels (13.3%), hydrolases (6.7%), and phosphodiesterases (13.3%).

The predicted kinase targeting activity is particularly significant in the context of antimalarial drug discovery. Protein kinases (PKs) have emerged as promising targets due to their essential roles in parasite growth and development throughout the life cycle [38]. The phylogenetic divergence between *Plasmodium* and human kinases offers opportunities for selective inhibition [39,40]. *Plasmodium*-specific kinases, including those regulating transmission to mosquitoes, have been identified and validated as potential drug targets through reverse genetics approaches [39,41]. The extensive knowledge gained from cancer research on kinase inhibition strategies could be leveraged for antimalarial development [38]. Furthermore, targeting the host cell kinases required for parasite survival presents an additional therapeutic approach [39]. With increasing resistance to current antimalarial drugs, the development of novel kinase inhibitors represents a promising strategy to combat malaria [40].

### 2.9. Toxicity Assessment and Biological Reactivity Profiling of Bioactive Compounds

While therapeutic compounds often demonstrate beneficial pharmacological effects, their administration can potentially induce toxicity through interactions between electrophilic drugs (or their metabolites) and nucleophilic biological macromolecules. Such interactions may occur with critical cellular components including DNA, glutathione (GSH), cyanide, and proteins, underscoring the importance of comprehensive safety evaluation for drug candidates [42,43]. Of particular relevance are uridine diphosphate glucuronosyltransferases (UGTs), which mediate the metabolism of approximately 15% of FDA-approved drugs and play a vital role in drug clearance and detoxification [44].

**Figure 17:**
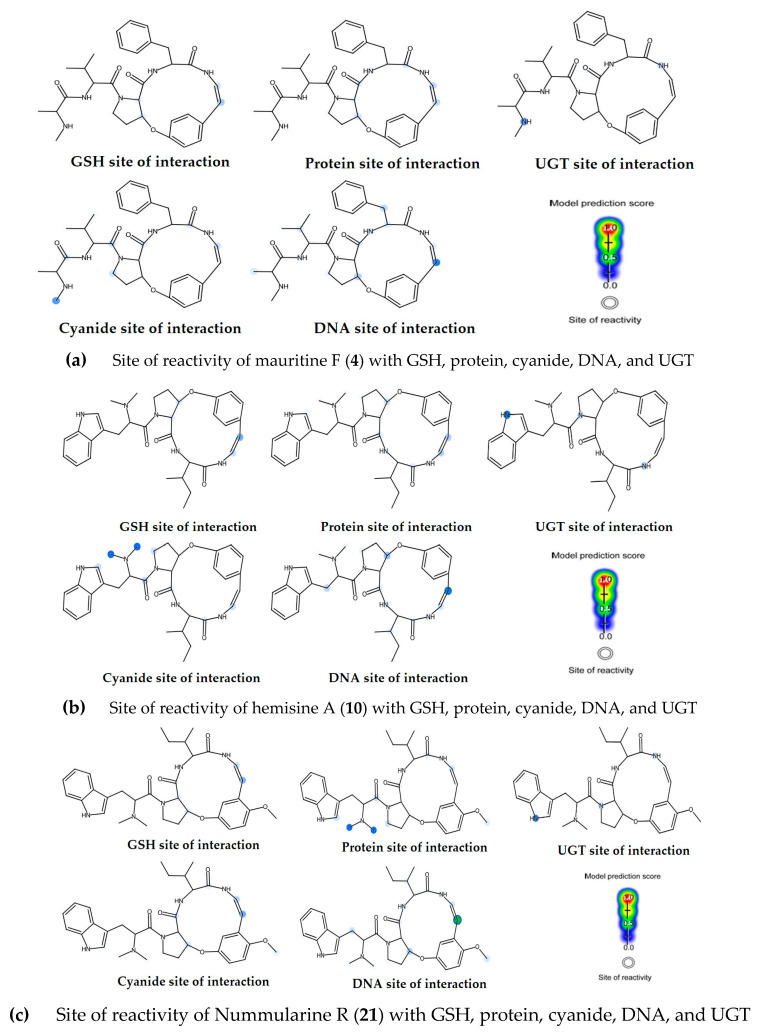
XenoSite reactivity model for compounds **4**, **10** and **21**, including GSH, protein, DNA and cyanide models. Red color indicates the highest scale for probability.

To assess the safety profiles of the most promising compounds (**4**, **10**, and **21**), their biological reactivity was evaluated using the XenoSite web tool (Figure 17) [42,43,44]. This analysis employed a colour-gradient visualization system where white circles marked known reactivity sites, with prediction scores ranging from 0 to 1 indicating the probability of atomic reactivity with specific biological targets. The reactivity profiling revealed favorable safety characteristics for these compounds. Both compounds **4** and **10** showed low probabilities of interaction with all four nucleophilic sites and UGT targets, with all scores remaining below 0.5. Compound **21** similarly demonstrated minimal reactivity potential with GSH, proteins, and cyanide. However, one carbon atom (C-1) in its double bond structure exhibited a moderate reactivity score of 0.5 for potential DNA interaction. These findings correlate well with the previously established bioavailability profiles of these compounds, providing additional support for their drug-like properties while highlighting specific structural features that may require consideration in further development.

### 2.10. Quantification of Bioactive Markers of Z. mauritiana Roots

The antiplasmodial evaluation revealed several bioactive constituents in *Z. mauritiana* roots. The EtOAc-soluble fraction contained zizyberenalic acid (**32**), which demonstrated significant biological activity. The alkaloid-rich fraction yielded six additional antiplasmodial compounds: mauritine F (**4**), mauritine M (**24**), nummularine B (**15**), nummularine R (**21**), hemisine A (**10**), and amphibine D (**12**). These compounds serve as crucial chemical markers for assessing the antiplasmodial potential of both the roots and other plant parts, enabling a correlation between phytochemical composition and medicinal activity.

**Figure 18:**
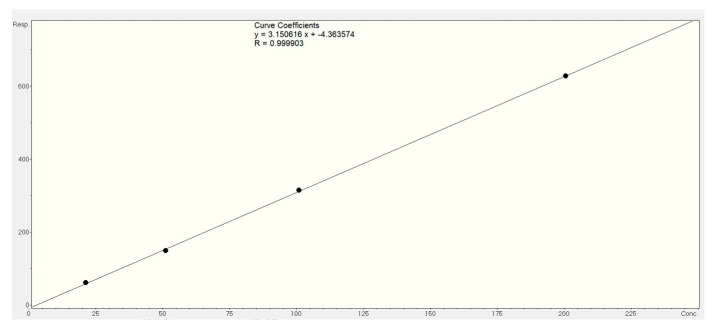
Calibration curve of zizyberenalic acid (**32**).

Absolute quantification was performed for zizyberenalic acid (**32**) and mauritine A (**5**) using authenticated reference standards and QuantAnalysis 4.3 software. The calibration curve for zizyberenalic acid (Figure 18) showed excellent linearity (y = 3.150616x − 4.363574; R = 0.999903), revealing a high concentration of 24.3 mg/g in the EtOAc fraction. This substantial quantity suggests that *Z. mauritiana* is a promising natural source of this bioactive compound.

Mauritine A (**5**) and amphibine A (**14**) quantifications (Figures S82–S84) produced the linear equation y_1_ = 189,021.2400x + 32,687,522 and y_2_ = 564,926.2857x + 161,164.86, respectively, with an exceptionally high concentration of 506.5 mg/g (mauritine A) and 11.4 mg/g (amphibine A) in the dichloromethane fraction. These values served as the basis for the relative quantification of other compounds in the sample. The complete quantitative profile of the analyzed compounds is presented in Table 7.

## 3. Materials and Methods

### 3.1. Equipment and General Experimental Procedure

Extracts were freed from solvent using rotatory evaporators (Büchi/Heidolph) under vacuum. UHPLC analyses were conducted using on a ThermoScientific Ultimate 3000 system (Waltham, MA, USA) equipped with a standard autosampler. Mass spectra were acquired using a Bruker Compact Q-TOF mass spectrometer (Billerica, MA, USA) with an ESI source. Sample dissolution was facilitated by an Emmi-H30 ultrasonicator (EMAG) (Emag, Salach, Germany) and ThermoScientific vortex mixer (Waltham, MA, USA). Fraction purification employed a Büchi Reveleris X2 MPLC system.

### 3.2. Plant Material

*Z. mauritiana* roots were collected in Katoual, Cameroon (10°31′0″ N, 14°12′0″ E), in June 2022 and authenticated by Mr. Tapsou (IRAD, Maroua). A voucher specimen (HEFG06825) was deposited at the Ecole de Faune Herbarium in Garoua.

### 3.3. Extraction, Fractionation, and Isolation

Air-dried roots (4.2 kg) were successively macerated with ethyl acetate (EtOAc) and ethanol (EtOH) (10 L each, 48 h × 3), yielding 127.2 g and 154.5 g of extracts, respectively. The ethanol extract exhibited antiplasmodial activity and was further fractionated, whereas the ethyl acetate extract was inactive. The ethanol extract was dissolved in dilute acid solution (pH, 3–4) and extracted with EtOAc to obtain an EtOAc-soluble fraction (FA, 70.2 g). The aqueous phase was then basified (pH ≈ 9) with 25% NH_4_OH and re-extracted with dichloromethane (CH_2_Cl_2_) to yield an alkaloid-rich fraction (FB, 4.6 g). Both fractions FA and FB were analyzed by LC-MS and subsequently purified using various chromatographic techniques. Meanwhile, the EtOAc extract was treated with *n*-hexane, leading to the formation of a precipitate (FZM1, 50.1 g) and a soluble fraction (FZM2, 70.3 g). Fraction FZM1 (2.5 g) was purified using medium-pressure liquid chromatography (MPLC) [column: Flashpure EcoFlex Silica 50 µm irregular (12 g); flow rate: 18 mL/min] with a gradient elution system: (a) *n*-hexane–acetone (23:2, *v*/*v*) (5 min), (b) *n*-hexane–acetone (23:2 → 22:3, *v*/*v*) (8 min), (c) *n*-hexane–acetone (22:3, *v*/*v*) (6 min), (d) *n*-hexane–acetone (22:3 → 3:1, *v*/*v*) (6 min), (e) *n*-hexane–acetone (3:1 → 1:1, *v*/*v*) (8 min). This purification yielded compounds **27** (500.7 mg), **30** (100.2 mg) and **26** (10.1 mg).

FZM2 and the EtOAc-soluble fraction (FA) from the ethanol extract were pooled together, based on their TLC profiles, and subjected to column chromatography (CC) over silica gel, eluting with a gradient of *n*-hexane–acetone (49:1 → 7:3, *v*/*v*), to afford five subfractions (FZA1–FZA5). Subfraction FZA1 was further purified by CC [*n*-hexane–acetone (49:1 → 9:1, *v*/*v*)] to yield compounds **32** (23.3 mg), **33** (15.6 mg), and a mixture of **33** and **34** (8.5 mg). Subfraction FZA2 was subjected to CC [*n*-hexane–acetone (9:1 → 17:3, *v*/*v*)] followed by MPLC [isocratic elution with *n*-hexane–acetone (23:2, *v*/*v*); column: Flashpure EcoFlex Silica 50 µm irregular (12 g); flow rate: 26 mL/min; duration: 22 min], yielding compounds **28** (20.6 mg) and **29** (26.0 mg). FZA4 was purified by CC [*n*-hexane–acetone (3:1 → 1:1, *v*/*v*)] to afford compound **36** (6.0 mg).

The alkaloid-rich fraction was subjected to CC over alumina using a gradient solvent system CH_2_Cl_2_/MeOH (23:2 → 4:1, *v*/*v*), yielding three subfractions (ZMDF1–ZMDF3). Subfraction ZMDF1 yielded compound **5** (25.4 mg) as a precipitate upon treatment with CH_2_Cl_2_/MeOH (23:2, *v*/*v*). Subfraction ZMDF2 was further purified over Sephadex LH-20 chromatography [eluent: CH_2_Cl_2_/MeOH (7:3, *v*/*v*)] to afford compound **6** (7.3 mg). Compound **14** (4.3 mg) was obtained from subfraction ZMDF3 using MPLC [Column: Flashpure Ecoflex 4 g (50 µm), flow rate: 10 mL/min, gradient elution: CH_2_Cl_2_ (3 min), CH_2_Cl_2_/MeOH (100:0 → 23:2, *v*/*v*) (6 min), CH_2_Cl_2_/MeOH (23:2, *v*/*v*) (4 min), CH_2_Cl_2_–MeOH (23:2 → 24:1, *v*/*v*) (3 min), CH_2_Cl_2_/MeOH (24:1 → 9:1, *v*/*v*) (5 min)].

### 3.4. UHPLC-DAD-ESI-QTOF MS/MS

High-resolution mass spectrometry analysis was performed using a Bruker QTOF spectrometer (Bremen, Germany) equipped with an electrospray ionization (ESI) source coupled to a Thermo Fisher Ultimate 3000 UHPLC system (Waltham, MA, USA). Mass spectra were acquired in both positive and negative ion modes (*m*/*z* 100–1500 range, 1.00 Hz scan rate) with automatic gain control, achieving mass accuracy within 0.40 ppm using sodium formate as the calibrant. The ESI source operated at spray voltages of +4.5 kV (positive mode) and −3.5 kV (negative mode), with a capillary temperature of 220 °C, using nitrogen as sheath gas (9 L/min). MS/MS analyses employed collision-induced dissociation (CID) with energies of 35 eV and 40 eV. Chromatographic separation was achieved using an Accucore C-18 reverse-phase column (50 × 2.1 mm, 2.6 μm, 150 Å) maintained at 35 °C, with a mobile phase consisting of water (0.1% formic acid, A) and acetonitrile (0.1% formic acid, B) at 0.4 mL/min. Two gradient elution methods were employed: Method 1 used 5% B (5 min isocratic), 5–60% B (7 min), 60% B (5 min isocratic), 60–95% B (4 min), and 95% B (2 min isocratic), followed by 1 min re-equilibration; Method 2 consisted of 5% B (10 min isocratic), 5–60% B (22 min), 60% B (3 min isocratic), 60–95% B (2 min), 95% B (1 min isocratic), and 95–5% B (1 min) with 1 min re-equilibration. Detection was performed using a diode array detector (DAD) scanning 190–600 nm with 10 μL injection volumes.

All samples were initially prepared at a concentration of 50 ppm for qualitative analysis. Molecular formula determination was performed using SmartFormula (DataAnalysis 4.3 software). LC-MS analyses of the fractions were carried out twice, and repeated more than a month later to check the accuracy of the results and the proper preservation of the samples.

For quantification analysis, calibration standards were prepared at concentrations of 200, 100, 50, and 20 ppm using authentic reference materials available in the laboratory. Quantification was achieved by generating calibration curves (peak area versus concentration) using QuantAnalysis software 4.3, with linear regression analysis applied to establish the relationship between the analyte concentration and detector response.

### 3.5. Advanced Mass Spectrometry Data Processing and Annotation

Mass spectrometry data analysis was performed using multiple computational approaches for comprehensive metabolite annotation. Initial LC-MS data processing was conducted using MZmine 4.3.0 [45]. For natural product annotation and spectral networking, SNAP-MS analysis was performed through the Natural Products Atlas online platform Natural Products Atlas|SNAP-MS (https://www.npatlas.org/) with the following parameters: mass error tolerance ≤ 10 ppm, GNPS cluster size range of 3–5000, minimum NP Atlas annotations cluster size of 3, maximum of 2000 nodes and 10,000 edges, using [M + H]^+^ as the target adduct. The COCONUT database [46] served as the reference for this analysis.

Feature-based molecular networking was executed on the GNPS2 platform [47,48] with precursor and fragment ion tolerances set at 0.5 Da, a minimum cosine score of 0.7, and at least three matched peaks required for spectral library matching. For in silico metabolite identification, three complementary GNPS workflows were employed: DEREPLICATOR+ [49] with strict mass tolerances of 0.01 Da for both precursor and fragment ions, a maximum charge state of 2, a minimum PSM score of 7, probabilistic fragmentation mode, and using the supercombined database; MolDiscovery [50] with similar parameters but a reduced minimum PSM score threshold of 6; and GNPS2 ChemWalker [51] focusing on [M + H]^+^ adducts with a mass tolerance of 15 ppm.

Additional structural elucidation was performed using SIRIUS software (version 5.8.6) for the in silico fragmentation and annotation of MS/MS data against multiple databases. All proposed structural annotations were further verified through a comparison with literature reports of known natural products [20,21,23].

### 3.6. Antiplasmodial Assay

The antiplasmodial activity was evaluated against the *P. falciparum* 3D7 strain (chloroquine-sensitive), obtained from BEI Resources (Manassas, VA, USA), using a modified Trager and Jensen culture method (2005) [52].

Parasites were maintained in fresh O^+^ human erythrocytes at 3% haematocrit in RPMI 1640 culture medium (Gibco, Paisley, UK) supplemented with 25 mM of HEPES, 1 × hypoxanthine (Gibco, Grand Island, NY, USA), 20 μg/mL gentamicin (Gibco, Shanghai, China), and 0.5% Albumax II (Gibco, Grand Island, NY, USA). Synchronization at the ring stage was achieved through sorbitol treatment, with parasites cultured for one complete cycle prior to experimentation.

Test compounds were prepared as 10 mM stock solutions in DMSO and serially diluted in incomplete RPMI 1640 medium. The assay was performed in 96-well plates containing parasite cultures (1% parasitemia, 1.5% hematocrit) exposed to compound concentrations ranging from 0.078 to 10 μM, with a final DMSO concentration of 0.1% (*v*/*v*). Chloroquine and artemisinin (0.0078 μM each) served as positive growth inhibition controls, while 0.1% DMSO-treated cultures functioned as negative controls. Following 72 h of incubation at 37 °C in a 5% CO_2_ atmosphere, parasite growth was quantified using a SYBR green I-based fluorescence assay. Briefly, a 3× concentrated SYBR Green lysis buffer was added to each well, followed by 30 min incubation in darkness. Fluorescence (excitation 485 nm, emission 538 nm) was acquired using a Fluoroskan Ascent microplate reader. IC_50_ values were determined via a nonlinear regression analysis of dose–response curves (log drug concentration vs. percent growth inhibition) using GraphPad Prism v8.0 (variable slope sigmoidal model). The antiplasmodial assay was performed in triplicate.

### 3.7. In Silico Toxicity, Pharmacokinetic Profiling, Drug-Likeness and Target Prediction

Computational ADMET (Absorption, Distribution, Metabolism, Excretion, and Toxicity) profiling was conducted using established bioinformatics platforms. Toxicity predictions were generated through ProTox-3.0 (https://tox.charite.de, accessed on 25 June 2024), which employs machine learning algorithms to estimate various toxicity endpoints. Pharmacokinetic properties and drug-likeness parameters were evaluated using the SwissADME web tool (http://www.swissadme.ch), which provides comprehensive predictions of key molecular characteristics, including Lipinski’s rule of five compliance, bioavailability, and membrane permeability.

Molecular target identification was performed using SwissTargetPrediction (http://www.swisstargetprediction.ch), which combines both 2D and 3D molecular similarity measures against known bioactive ligands to predict probable protein targets. Additionally, potential sites of metabolic reactivity were analyzed using the Xenosite web server (http://xenosite.org), which predicts susceptible molecular regions for phase I and II metabolic transformations.

## 4. Conclusions

This study systematically evaluated the antiplasmodial properties of *Z. mauritiana* roots using an integrated analytical approach combining UHPLC-MS/MS molecular networking, bioactivity-guided fractionation, and computational ADMET prediction. The ethanol extract and its derived fractions (ethyl acetate-soluble and alkaloid-rich) demonstrated potent activity against the chloroquine-sensitive *P. falciparum* 3D7 strain, with IC_50_ values of 32.70, 4.75, and 11.35 µg/mL, respectively. Comprehensive metabolite profiling identified 32 bioactive compounds, comprising 25 cyclopeptide alkaloids (including five previously putative undescribed structures) and seven ceanothane-type triterpenoids. Among these, betulinic acid (IC_50_ = 19.0 µM) and zizyberenalic acid (IC_50_ = 20.45 µM) emerged as the most potent antiplasmodial agents. In silico pharmacokinetic evaluation highlighted mauritine F, hemisine A, and nummularine R as particularly promising lead compounds, exhibiting favorable drug-like properties, good bioavailability, and low toxicity profiles. Their predicted interactions with family A G protein-coupled receptors and the protein kinases taking advantage of significant phylogenetic differences between *Plasmodium* and human kinases suggests a potentially selective mechanism of antimalarial action. Quantitative analysis revealed remarkably high concentrations of key bioactive constituents, with zizyberenalic acid (24.3 mg/g) and mauritine A (506.5 mg/g) being particularly abundant. These findings establish *Z. mauritiana* as an exceptionally rich source of novel antiplasmodial compounds that warrant further investigation as potential therapeutic candidates against drug-resistant malaria.

## Figures and Tables

**Table 1:** Tentatively identified compounds from the alkaloid-rich fraction of the roots of *Z. mauritiana*.

Peak N°	RT (mins)	*m*/*z* (Experimental)	*m*/*z* (Theoretical)	Adduct	Molecular Formula	MS/MS Fragments	Potential Compounds	GNPS Dereplicator+	GNPS Moldiscovery	GNPS2 ChemWalker	Sirius	FBMN (MS/MS Spectral Database)	Literature Review Data
1	7.74	592.3135	592.3130	[M + H]^+^	C_32_H_41_N_5_O_6_	394, 366, 199, 171	Mauritine E (**1**)	×	✓	✓	✓	×	✓
2	7.97	491.2651	491.2653	[M + H]^+^	C_28_H_34_N_4_O_4_	378, 203, 186, 86	Mauritine C (**2**)	✓	✓	✓	✓	×	✓
3	8.03	505.2804	505.2809	[M + H]^+^	C_29_H_36_N_4_O_4_	378, 203, 100	Amphibine F (**3**)	✓	✓	✓	✓	×	✓
4	8.08	562.3020	562.3024	[M + H]^+^	C_31_H_39_N_5_O_5_	378, 203, 185, 157	Mauritine F (**4**)	✓	✓	✓	✓	×	✓
5	8.14	576.3180	576.3180	[M + H]^+^	C_32_H_41_N_5_O_5_	378, 199, 185, 17 1	Mauritine A (**5**)	✓	✓	✓	✓	×	✓
6	8.15	608.3448	608.3443	[M + H]^+^	C_33_H_46_N_5_O_6_	576, 378, 199, 171	1,2-dihydro-2*β*-methoxymauritine A (**6**)	✓	×	×	×	×	✓
7	8.33	592.3135	592.3133	[M + H]^+^	C_32_H_41_N_5_O_6_	531, 378, 215, 116, 72	Mauritine A *N*-oxide (**7**)	✓	✓	×	✓	×	✓
8	8.45	574.3018	574.3024	[M + H]^+^	C_32_H_39_N_5_O_5_	378, 197, 169, 141	Apetaline B (**8**)	✓	✓	✓	✓	×	✓
9	8.47	590.3326	590.3337	[M + H]^+^	C_33_H_43_N_5_O_5_	378, 350, 213, 199, 185, 171	Mauritine H (**9**)	✓	✓	✓	✓	×	✓
10	8.49	558.3073	558.3075	[M + H]^+^	C_32_H_39_N_5_O_4_	513, 427, 187, 170	Hemisine A/Amphibine G (**10**)	✓	✓	✓	✓	×	✓
11	8.58	622.3603	622.3599	[M + H]^+^	C_33_H_43_N_5_O_7_	576, 424 378, 199, 171	(**11**)	×	×	×	×	×	×
12	8.83	632.3804	632.3806	[M + H]^+^	C_36_H_49_N_5_O_5_	344, 289, 148, 114	Amphibine D (**12**)	✓	×	✓	✓	×	✓
13	8.89	535.3277	535.3279	[M + H]^+^	C_31_H_42_N_4_O_4_	287, 148	Lotusanine A (**13**)	×	×	✓	✓	×	✓
14	8.98	574.3377	574.3388	[M + H]^+^	C_33_H_43_N_5_O_4_	529, 443, 187, 170	Discarine A/Amphibine A (**14**)	✓	✓	✓	✓	×	✓
15	9.01	592.3120	592.3130	[M + H]^+^	C_32_H_41_N_5_O_4_	408, 185, 157	Nummularine B (**15**)	✓	✓	✓	✓	×	✓
16	9.03	606.3277	606.3286	[M + H]^+^	C_33_H_43_N_5_O_6_	408, 199, 171, 72	Amphibine H (**16**)	✓	✓	✓	✓	×	✓
17	9.10	657.3757	657.3759	[M + H]^+^	C_37_H_48_N_6_O_5_	513, 344, 314, 286, 155, 100	Mauritine J (**17**)	✓	×	✓	✓	×	✓
18	9.11	632.3802	632.3806	[M + H]^+^	C_36_H_49_N_5_O_5_	378, 255, 227, 155	Amphibine C (**18**)	✓	✓	✓	✓	×	✓
19	9.20	671.3915	671.3915	[M + H]^+^	C_38_H_50_N_6_O_5_	344, 328, 203 114	Amphibine E (**19**)	✓	✓	✓	✓	×	✓
20	9.32	620.3446	620.3443	[M + H]^+^	C_34_H_45_N_5_O_6_	408, 213, 199, 185, 171	(**20**)	×	×	×	×	×	×
21	9.52	588.3189	588.3180	[M + H]^+^	C_33_H_41_N_5_O_5_	543, 457, 199, 187, 170	Nummularine R (**21**)	✓	×	✓	✓	×	✓
22	9.74	606.3278	606.3286	[M + H]^+^	C_33_H_41_N_5_O_5_	408, 380, 199, 171	(**22**)	×	×	×	×	×	×
23	9.82	662.3902	662.3912	[M + H]^+^	C_37_H_51_N_5_O_6_	408, 289, 255, 227, 148, 114	(**23**)	×	×	×	×	×	×
24	9.91	687.3859	687.3865	[M + H]^+^	C_38_H_50_N_6_O_6_	543, 374, 314, 155, 286, 100	Mauritine M (**24**)	✓	✓	×	✓	×	✓
25	10.00	701.4023	701.4021	[M + H]^+^	C_39_H_52_N_6_O_6_	374, 328, 300, 155, 114	(**25**)	×	×	×	×	×	×

✓: annotated ×: not annotated.

**Table 2:** Tentatively identified compounds from the EtOAc soluble fraction of the roots of *Z. mauritiana*.

Peak N°	RT (mins)	*m*/*z* (Experimental)	*m*/*z* (Theoretical)	Adduct	Molecular Formula	MS/MS Fragments	Potential Compounds
1	3.9	329.2186	/	/	/	/	Not identified
2	4.3	501.3226	501.3222	[M − H]^−^	C_30_H_46_O_6_	485, 453, 439, 427, 423, 409	24-hydroxyceanothic acid (**26**)
3	4.7	485.3275	485.3272	[M − H]^−^	C_30_H_46_O_5_	423	Ceanothic acid (**27**)
4	4.9	485.3276	/	/	/	423	Not identified
5	5.0	471.3387	/	/	/	/	Not identified
6	5.1	295.2401	/	/	/	/	Not identified
7	5.3	469.3332	469.3330	[M − H]^−^	C_30_H_46_O_4_	/	Zizyberanalic acid (**28**)
8	5.4	485.3276	/	/	/	423	Not identified
9	5.6	469.3323	/	/	/	/	Not identified
10	5.9	453.3002	453.3010	[M − H]^−^	C_29_H_42_O_4_	/	Ceanothenic acid (**29**)
11	6.5	455.3531	455.3531	[M − H]^−^	C_30_H_48_O_3_	/	Betulinic acid (**30**)
12	6.8	439.3877	/	/	/	/	Not identified
13	6.9	279.2492	/	/	/	/	Not identified
14	7.0	453.3378	453.3374	[M − H]^−^	C_30_H_46_O_3_	/	Betulonic acid (**31**)
15	7.1	451.3212	451.3218	[M − H]^−^	C_30_H_44_O_3_	/	Zizyberenalic acid (**32)**
16	7.3	281.2625	/	/	/	/	Not identified

**Table 3:** Classification of CPAs type I core.

	CPAs Type I Core				
	Ia			Ib	Ic
	Ia1	Ia2	Ia3	/	/
Number of atoms in the ring	14	14	14	13	15
C unit	leucine	phenylalanine	proline	proline	/

**Table 4:** Antiplasmodial activity of extracts, fractions and compounds.

Extracts and Fractions	IC_50_ *Pf*3D7 (µg/mL)	IC_50_ *Pf*K1 (µg/mL)	References
ZMRE	32.70	NT	/
ZMRA	>50	NT	/
ZMRD	4.75	NT	/
ZMRA II	11.35	NT	/
**Compounds**	(µM)	(µM)	/
Zizyberenalic acid (**32**)	20.45	6.62 *	[31]
Betulinic acid (**30**)	19.0	/	
Mauritine F (**4**)	NT	34.2 *	[32]
Mauritine M (**24**)	NT	3.7 *	[15]
Nummularine B (**15**)	NT	3.6 *	[32]
Nummularine R (**21**)	NT	3.2 *	[32]
Hemisine A (**10**)	NT	7.3 *	[15]
Amphibine D (**12**)	NT	8.9 *	[32]
**Reference drugs**	(nM)	/	/
Chloroquine	29.9	/	/
Artemisinin	26.3	/	/

ZMRE: EtOH extract, ZMRA: EtOAc extract, ZMRD: Alkaloid-rich fraction, ZMRA II: EtOAc fraction, * activity values from the literature, NT: not tested.

**Table 5:** In silico acute toxicity of bioactive compounds.

	Targets	Hepatotoxicity (SP)	Nephrotoxicity (SP)	Cardiotoxicity (SP)	Respiratory toxicity (SP)	LD_50_ (mg/kg)
Compounds						
Mauritine F (**4**)		Inactive (0.65)	Inactive (0.57)	Inactive (0.79)	Active (0.82)	550
Hemisine A (**10**)		Inactive (0.63)	Inactive (0.58)	Inactive (0.83)	Active (0.83)	550
Amphibine D (**12**)		Inactive (0.57)	Inactive (0.60)	Inactive (0.83)	Active (0.77)	550
Nummularine B (**15**)		Inactive (0.64)	Inactive (0.52)	Inactive (0.77)	Active (0.79)	200
Nummularine R (**21**)		Inactive (0.61)	Inactive (0.51)	Inactive (0.81)	Active (0.77)	386
Mauritine M (**24**)		Inactive (0.64)	Active (0.51)	Inactive (80)	Active (0.72)	386
Zizyberenalic acid (**32**)		Inactive (0.59)	Inactive (0.63)	Inactive (0.60)	Active (0.72)	1000
Artemisinin		Inactive (0.72)	Inactive (0.56)	Inactive (0.57)	Inactive (0.59)	4228
Chloroquine		Inactive (0.90)	Inactive (0.81)	Inactive (0.96)	Inactive (0.91)	750

SP: Score of the prediction. Average for active compounds: Hepatotoxicity: 0.82; Nephrotoxicity: 0.75; Cardiotoxicity: 0.86; Respiratory toxicity: 0.78. Class I: fatal if swallowed (LD50 ≤ 5 mg/kg); Class II: fatal if swallowed (5 mg/kg < LD50 ≤ 50 mg/kg); Class III: toxic if swallowed (50 mg/kg < LD50 ≤ 300 mg/kg); Class IV: harmful if swallowed (300 mg/kg < LD50 ≤ 2000 mg/kg); Class V: may be harmful if swallowed (2000 mg/kg < LD50 ≤ 5000 mg/kg); Class VI: non-toxic (LD50 > 5000 mg/kg).

**Table 6:** In silico pharmacokinetic parameters and drug-likeness of some compounds.

Compounds	Mauritine F (4)	Hemisine A (10)	Amphibine D (12)	Nummularine B (15)	Nummularine R (21)	Mauritine M (24)	Zizyberenalic acid (32)
Physicochemical Parameters							
Molecular weight	561.67	557.68	631.80	591.70	587.71	686.84	452.67
Number of rotatable bonds	9	15	12	10	8	13	3
Number of heavy atoms	41	41	46	43	43	50	33
Fraction C(sp3)	0.42	0.41	0.50	0.44	0.42	0.47	0.80
Molar Refractivity	166.84	170.99	190.97	173.34	177.48	204.42	135.48
TPSA	128.87 Å^2^	106.77 Å^2^	120.08 Å^2^	138.10 Å^2^	116.00 Å^2^	153.89 Å^2^	54.37 Å^2^
Lipophilicity							
log *P*_o/w_ (MLOGP)	0.73	1.48	1.65	0.42	1.16	0.88	5.63
log *P*_o/w_ (XLOGP3)	3.15	4.36	5.30	3.12	4.33	4.93	8.04
Water solubility							
log *S* (ESOL)	−4.95	−5.85	−6.50	−5.02	−5.94	6.57	−7.51
Water solubility class	Moderately soluble	Moderately soluble	Poorly soluble	Moderately soluble	Moderately soluble	Poorly soluble	Poorly soluble
Pharmacokinetic							
GI absorption	High	High	High	High	High	Low	Low
BBB permeant	No	No	No	No	No	No	No
P-gp substrate	Yes	Yes	Yes	Yes	Yes	Yes	No
CYP1A2 inhibitor	No	No	No	No	No	No	No
CYP2C19 inhibitor	No	No	No	No	No	No	No
CYP2C9 inhibitor	No	No	No	No	No	No	Yes
CYP2D6 inhibitor	No	No	No	No	No	No	No
CYP3A4 inhibitor	Yes	Yes	Yes	Yes	Yes	Yes	No
log *K*_p_ (skin permeation)	−7.49 cm/s	−6.61 cm/s	−6.39 cm/s	−7.69 cm/s	−6.81 cm/s	−6.99 cm/s	−3.35 cm/s
Drug likeness							
Lipinski	Yes; 1 violation	Yes; 1 violation	Yes; 1 violation	No; 2 violations	Yes; 1 violation	No; 2 violations	Yes; 1 violation
Ghose	No; 3 violations	No; 3 violations	No; 3 violations	No; 3 violations	No; 3 violations	No; 3 violations	No; 3 violations
Veber	Yes	Yes	No; 1 violation	Yes	Yes	No; 2 violations	Yes; 1 violation
Egan	Yes	Yes	Yes	No; 1 violation	Yes	No; 1 violation	Yes; 1 violation
Bioavailability Score	0.55	0.55	0.55	0.17	0.55	0.17	0.85

MW: Molecular weight of the compounds; TPSA: Topological Polar Surface Area; BBB: Blood–brain barrier; P-pg: P-glycoprotein; Water solubility (log S. Scale: Insoluble < −10 < Poorly < −6 < moderately < −4 < soluble < −2 < Very < 0 < Highly 5; Lipinski’s rule: MW ≤ 500, MLOGP ≤ 4.15, N or O ≤ 10, NH or OH ≤ 5, Ghose’s rule: 160 ≤ MW ≤ 480, −0.4 ≤ MLOGP ≤ 5, 40 ≤ Molar refractivity ≤ 130, 20 ≤ Number of heavy atoms ≤ 70; Veber’s rule: Rotatable bonds ≤ 10, TPSA ≤ 140; Egan’s rules: WLOGP ≤ 5.88, TPSA ≤ 131.6 CYP2D6: Cytochrome P450 family 2 subfamily D member 6; CYP1A2: Cytochrome P450 family 1 subfamily A member 2; CYP2C19: Cytochrome P450 family 2 subfamily C member 19, CYP2C9: Cytochrome P450 family 2 subfamily C member 9; CYP3A4: Cytochrome P450 family 3 subfamily A member 4.

**Table 7:** Relative quantification of compounds from dichloromethane fraction.

Compounds	Area (In 0.2 mg of ZMRD)	RMCY1 (mg/g of ZMRD)	RMCY2 (mg/g of ZMRD)	Mean ± SD (mg/g)
Mauritine F (**4**)	3,934,027	38.44	31.23	34.83 ± 3.60
Mauritine A (**5**)	51,835,504	506.5	411.51	459.00 ± 47.49
Hemisine A (**10**)	765,130	7.47	6.07	6.67 ± 0.70
Amphibine D (**12**)	416,025	4.06	3.30	3.68 ± 0.38
Nummularine B (**15**)	570,694	5.57	4.53	5.05 ± 0.52
Nummularine R (**21**)	87,149	0.85	0.69	0.77 ± 0.08
Mauritine M (**24**)	513,715	5.01	4.07	4.54 ± 0.47
Amphibine A (**14**)	1,435,994	14.03	11.4	12.71 ± 1.35

RMCY1: relative mass concentration using linear equation y_1_; RMCY2: relative mass concentration using linear equation y_2_, ZMRD: alkaloid-rich fraction.

## Data Availability

The original contributions presented in this study are included in the article/Supplementary Materials. Further inquiries can be directed to the corresponding authors (Y.S.F.F. and B.N.L).

## Supplementary Materials

The following supporting information can be downloaded at: https://www.mdpi.com/article/10.3390/molecules30142958/s1, Figure S1: (+) HRESIMS spectrum of compound **1** (*m*/*z* 592); Figure S2: (+) ESI MS/MS spectrum of compound **1** (*m*/*z* 592); Figure S3: (+) HR-ESI-MS spectrum of compound **2** (*m*/*z* 491); Figure S4: (+) HR-ESI-MS/MS spectrum of compound **2** (*m*/*z* 491); Figure S5: (+) HRESIMS spectrum of compound **3** (*m*/*z* 505); Figure S6: (+) ESI MS/MS spectrum of compound **3** (*m*/*z* 505); Figure S7: (+) HRESIMS spectrum of compound **4** (*m*/*z* 562); Figure S8: (+) ESI MS/MS spectrum of compound **4** (*m*/*z* 562); Figure S9: (+) HR-ESI-MS spectrum of compound **5** (*m*/*z* 576); Figure S10: (+) HR-ESI-MS/MS spectrum of compound **5** (*m*/*z* 576); Figure S11: (+) HR-ESI-MS spectrum of compound **6** (*m*/*z* 608); Figure S12: (+) HR-ESI-MS/MS spectrum of compound **6** (*m*/*z* 608); Figure S13: (+) HRESIMS of compound **7** (*m*/*z* 592); Figure S14: (+) HRESIMS/MS of compound **7** (*m*/*z* 592); Figure S15: (+) HRESIMS spectrum of compound **8** (*m*/*z* 574); Figure S16: (+) HRESI MS/MS spectrum of compound **8** (*m*/*z* 574); Figure S17: (+) HRESIMS/MS 9 (*m*/*z* 590); Figure S18: (+) ESIMS/MS 9 (*m*/*z* 590); Figure S19: (+) HRESIMS spectrum of compound **10** (*m*/*z* 558); Figure S20: (+) ESI MS/MS spectrum of compound **10** (*m*/*z* 558); Figure S21: (+) HRESIMS spectrum of compound **11** (*m*/*z* 622); Figure S22: (+) ESI MS/MS spectrum of compound **11** (*m*/*z* 622); Figure S23: (+) ESI MS/MS spectrum of compound **12** (*m*/*z* 632); Figure S24: (+) ESI MS/MS spectrum of compound **12** (*m*/*z* 632); Figure S25: (+) HRESIMS/MS of compound **13** (*m*/*z* 535); Figure S26: (+) HRESIMS/MS compound **13** (*m*/*z* 535); Figure S27: (+) HRESIMS/MS of compound **14** (*m*/*z* 574); Figure S28: (+) HRESIMS/MS of compound **14** (*m*/*z* 574); Figure S29: (+) ESI MS/MS spectrum of compound **15** (*m*/*z* 592); Figure S30: (+) ESI MS/MS spectrum of compound **15** (*m*/*z* 592); Figure S31: (+) HRESIMS of compound **16** (*m*/*z* 606); Figure S32: (+) HRESIMS/MS of compound **16** (*m*/*z* 606); Figure S33: (+) HRESIMS spectrum of compound **17** (*m*/*z* 657); Figure S34: (+) ESI MS/MS spectrum of compound **17** (*m*/*z* 657); Figure S35: (+) HRESIMS spectrum of compound **18** (*m*/*z* 632); Figure S36: (+) ESI MS/MS spectrum of compound **18** (*m*/*z* 632); Figure S37: (+) HRESIMS of compound **19** (*m*/*z* 671); Figure S38: (+) HRESIMS/MS of compound **19** (*m*/*z* 671); Figure S39: (+) HRESIMS spectrum of compound **20** (*m*/*z* 620); Figure S40: (+) ESI MS/MS spectrum of compound **20** (*m*/*z* 620); Figure S41: (+) HRESIMS of compound **21** (*m*/*z* 588); Figure S42: (+) HRESIMS/MS of compound **21** (*m*/*z* 588); Figure S43: (+) HRESIMS/MS of compound **22** (*m*/*z* 606); Figure S44: (+) HRESIMS/MS of compound **22** (*m*/*z* 606); Figure S45: (+) HRESIMS spectrum of compound **23** (*m*/*z* 662); Figure S46: (+) ESI MS/MS spectrum of compound **23** (*m*/*z* 662); Figure S47: (+) HRESIMS of compound **24** (*m*/*z* 687); Figure S48: (+) HRESIMS/MS of compound **24** (*m*/*z* 687); Figure S49: (+) HRESIMS spectrum of compound **25** (*m*/*z* 701); Figure S50: (+) ESI MS/MS spectrum of compound **25** (*m*/*z* 701); Figure S51: (+) HRESIMS spectrum of compound **26** (*m*/*z* 501); Figure S52: (+) HRESIMS spectrum of compound **27** (*m*/*z* 485); Figure S53: (+) HRESIMS spectrum of compound **28** (*m*/*z* 469); Figure S54: (+) HRESIMS spectrum of compound **29** (*m*/*z* 453); Figure S55: (+) HRESIMS spectrum of compound **30** (*m*/*z* 455); Figure S56: (+) HRESIMS spectrum of compound **31** (*m*/*z* 453); Figure S57: (+) HRESIMS spectrum of compound **32** (*m*/*z* 451); Figure S58: ^1^HNMR spectrum (CDCl_3_, 600 MHz) of compound **5**; Figure S59: ^13^CNMR spectrum (CDCl_3_, 150 MHz) of compound **5**; Figure S60: ^1^HNMR spectrum (CDCl_3_+ CD_3_OD, 600 MHz) of compound **6**; Figure S61: ^13^CNMR spectrum (CDCl_3_ + CD_3_OD, 150 MHz) of compound **6**; Figure S62: ^1^HNMR spectrum (CDCl_3_+ CD_3_OD, 600 MHz) of compound **14**; Figure S63: ^13^CNMR spectrum (CDCl_3_+ CD_3_OD, 150 MHz) of compound **14**; Figure S64: ^1^H NMR spectrum (CD_3_OD, 600 MHz) of compound **26**; Figure S65: ^13^CNMR spectrum (CD_3_OD, 150 MHz) of compound **26**; Figure S66: ^1^H NMR spectrum (CDCl_3_/CD_3_OD, 600 MHz) of compound **27**; Figure S67: ^13^C NMR spectrum (CDCl_3_/CD_3_OD, 150 MHz) of compound **27**; Figure S68: ^1^HNMR spectrum (CD_3_OD, 600 MHz) of compound **28**; Figure S69: ^13^CNMR spectrum (CD_3_OD, 600 MHz) of compound **28**; Figure S70: ^1^HNMR spectrum (DMSO-*d_6_*, 600 MHz) of compound **29**; Figure S71: ^13^CNMR spectrum of (DMSO-*d_6_*, 150 MHz) of compound **29**; Figure S72: ^1^H NMR spectrum (CDCl_3_/CD_3_OD, 600 MHz) of **30**; Figure S73: ^13^C NMR spectrum (CDCl_3_/CD_3_OD, 150 MHz) of compound **30**; Figure S74: ^1^H NMR spectrum (CDCl_3_, 600 MHz) of compound **32**; Figure S75: ^13^C NMR spectrum (CDCl_3_, 150 MHz) of compound **32**; Figure S76: ^1^H NMR spectrum (CDCl_3_, 500 MHz) of compound **33**; Figure S77: ^13^C NMR spectrum (CDCl_3_, 125 MHz) of compound **33**; Figure S78: ^1^H NMR spectrum (CDCl_3_, 600 MHz) of compound **34** and **35**; Figure S79: ^13^C NMR spectrum (CDCl_3_, 600 MHz) of compound **34** and **35**; Figure S80: ^1^HNMR spectrum (DMSO-*d*_6_, 600 MHz) of compound **36**; Figure S81: ^13^CNMR spectrum (DMSO-*d*_6_, 600 MHz) of compound **36**; Figure S82: Calibration curve of mauritine A (5) using BPC; Figure S83: Calibration curve of mauritine A (5) using UV (λ = 254 nM). Figure S84: Pie chart of molecular targets of Artemisinin; Figure S85: Pie chart of molecular targets of Chloroquine; Figure S86: Calibration curve of amphibine A (**14**) using EIC.

## References

[1] World Malaria Report 2024. https://www.who.int/teams/global-malaria-programme/reports/world-malaria-report-2024.

[2] Asua V., Conrad M.D., Aydemir O., Duvalsaint M., Legac J., Duarte E., Tumwebaze P., Chin D., Cooper R., Yeka A. (2021). Changing Prevalence of Potential Mediators of Aminoquinoline, Antifolate, and Artemisinin Resistance Across Uganda. J. Infect. Dis..

[3] Rosenthal M.R., Ng C.L. (2021). A proteasome mutation sensitizes *P. falciparum* Cam3.II K13C580Y Parasites to DHA and OZ439. ACS Infect. Dis..

[4] Jha D., Hangargekar P., Akbar M., Parihar A.S., Kashyap S., Joshi A., Rahman M.A. (2024). *Ziziphus mauritiana*: An in-depth Review of its Medicinal Attributes and Pharmacological Activities. Intell. Pharm..

[5] Mishra T., Bhatia A. (2014). Antiplasmodial effects of the aqueous ethanolic seed extract of *Ziziphus mauritiana* against *Plasmodium berghei* in Swiss albino mice. Int. J. Pharmacol. Res..

[6] Goyal M., Sasmal D., Nagori B. (2012). Review on ethnomedicinal uses, Pharmacological activity and phytochemical constituents of *Ziziphus mauritiana* & *jujuba* Lam., non Mill). J. Complement. Med. Drug Discov..

[7] Orwa C., Mutua A., Kindt R., Jamnadass R., Anthony S. (2009). Agroforestree Database: A Tree Reference and Selection Guide Version 4.0. https://apps.worldagroforestry.org/treedb/AFTPDFS/Ziziphus_mauritiana.pdf.

[8] Prakash O., Usmani S., Singh R., Singh N., Gupta A., Ved A. (2021). A panoramic view on phytochemical, nutritional, and therapeutic attributes of *Ziziphus mauritiana* Lam.: A comprehensive review. Phytother. Res..

[9] Panseeta P., Suksamrarn S. (2010). Triterpenes from the root of the *Ziziphus mauritiana*. JST.

[10] Nilofar N., Sinan K.I., Dall’Acqua S., Sut S., Uba A.I., Etienne O.K., Ferrante C., Ahmad J., Zengin G. (2024). *Ziziphus mauritiana* Lam. Bark and Leaves: Extraction, Phytochemical Composition, In Vitro Bioassays and In Silico Studies. Plants.

[11] Ramar M.K., Chidambaram K., Chandrasekaran B., Kandasamy R. (2022). Standardization, in-silico and in-vivo safety assessment of methanol extract of *Ziziphus mauritiana* Lam leaves. Regul. Toxicol. Pharmacol..

[12] Khanam A., Ijaz Hussain A., Mohammed E.H., Nahar L., Rathore H.A. (2025). Phenolic Profile of Seedless *Ziziphus mauritiana* Fruits and Leaves Extracts with In Vivo Antioxidant and Anti-Inflammatory Activities: Influence on Pro-Inflammatory Mediators. Chem. Biodivers..

[13] Guo S., Duan J.-A., Li Y., Wang R., Yan H., Qian D., Tang Y., Su S. (2017). Comparison of the Bioactive Components in Two Seeds of Ziziphus Species by Different Analytical Approaches Combined with Chemometrics. Front. Pharmacol..

[14] Soraya S., Sukara E., Sinaga E. (2022). Identification of Chemical Compounds in *Ziziphus mauritiana* Fruit Juice by GC-MS and LC-MS/MS Analysis. Int. J. Biol. Phys. Chem. Stud..

[15] Panseeta P., Lomchoey K., Prabpai S., Kongsaeree P., Suksamrarn A., Ruchirawat S., Suksamrarn S. (2011). Antiplasmodial and antimycobacterial cyclopeptide alkaloids from the root of *Ziziphus mauritiana*. Phytochemistry.

[16] Batool M., Afzal S., Afzal K., Ahmed B., Abbas K., Muhammad S.A., Qadir M.I. (2019). Anticancer activity of *Ziziphus mauritiana* roots against human breast cancer cell line. Pak. J. Pharm. Sci..

[17] Shady N.H., Soltane R., Maher S.A., Saber E.A., Elrehany M.A., Mostafa Y.A., Sayed A.M., Abdelmohsen U.R. (2022). Wound Healing and Antioxidant Capabilities of *Zizyphus mauritiana* Fruits: In-Vitro, In-Vivo, and Molecular Modeling Study. Plants.

[18] Dos Santos C.H.C., De Carvalho M.G., Laub A., Franke K., Wessjohann L. (2021). UHPLC-ESI-Orbitrap-HR-MS Analysis of Cyclopeptide Alkaloids from *Ziziphus joazeiro*. Nat. Prod. Commun..

[19] Kang K.B., Jang D.S., Kim J., Sung S.H. (2016). UHPLC-ESI-qTOF-MS analysis of cyclopeptide alkaloids in the seeds of *Ziziphus jujuba* var. spinosa. Mass Spectrom. Lett..

[20] Collett L.A., Davies-Coleman M.T., Gournelis D.C., Laskaris G.G., Rivett D.E.A., Verpoorte R. (1998). Fortschritte der Chemie Organischer Naturstoffe/Progress in the Chemistry of Organic Natural Products.

[21] Tschesche R., Kaubmann E.U., Manske R.H.F. (1975). The cyclopeptide alkaloids. The Alkaloids: Chemistry and Physiology.

[22] Blaženović I., Kind T., Ji J., Fiehn O. (2018). Software tools and approaches for compound identification of LC-MS/MS data in metabolomics. Metabolites.

[23] Tan N.-H., Zhou J. (2006). Plant cyclopeptides. Chem. Rev..

[24] Ji C.-J., Zeng G.-Z., Han J., He W.-J., Zhang Y.-M., Tan N.-H. (2012). Zizimauritic acids A–C, three novel nortriterpenes from *Ziziphus mauritiana*. Bioorg. Med. Chem. Lett..

[25] Muñoz-Nuñez E., Quiroz-Carreño S., Pastene-Navarrete E., Seigler D.S., Céspedes-Acuña C., Martínez Valenzuela I., Oppliger Muñoz M., Salas-Burgos A., Alarcón-Enos J. (2022). Assessments of Ceanothanes Triterpenes as Cholinesterase Inhibitors: An Investigation of Potential Agents with Novel Inspiration for Drug Treatment of Neurodegenerative Diseases. Metabolites.

[26] Pierre L.L., Moses M.N. (2015). Isolation and characterisation of stigmasterol and *β*-sitosterol from *Odontonema Strictum* (Acanthaceae). J. Innov. Pharm. Biol. Sci..

[27] Peshin T., Kar H. (2017). Isolation and characterization of *β*-sitosterol-3-O-*β*-D-glucoside from the extract of the flowers of *Viola odorata*. Br. J. Pharm. Res..

[28] Adam I.A., Irshad R., Wahab A.T., Omoboyowa D.A., Choudhary M.I., Wang Y. (2022). Two new 5 (14)-membered type cyclopeptide alkaloids from root bark of *Ziziphus spina*-*christi* (L.) Desf. Nat. Prod. Res..

[29] Han J., Ji C.-J., He W.-J., Shen Y., Leng Y., Xu W.-Y., Fan J.-T., Zeng G.-Z., Kong L.-D., Tan N.-H. (2011). Cyclopeptide alkaloids from *Ziziphus apetala*. J. Nat. Prod..

[30] Karim M.A., Hossain M.K., Al-Mansur M.A., Shajib M.S., Rashid M.A. (2019). Isolation of zizyberenalic acid and biological studies of *Ziziphus mauritiana* Lam. growing in Bangladesh. Bangladesh J. Bot..

[31] Suksamrarn S., Panseeta P., Kunchanawatta S., Distaporn T., Ruktasing S. (2006). Ceanothane- and lupane-type triterpenes with antiplasmodial and antimycobacterial activities from *Ziziphus cambodiana*. Chem. Pharm. Bull..

[32] Tuenter E., Segers K., Kang K., Viaene J., Sung S., Cos P., Maes L., Heyden Y., Pieters L. (2017). Antiplasmodial activity, cytotoxicity and Structure-Activity Relationship Study of Cyclopeptide Alkaloids. Molecules.

[33] Banerjee P., Kemmler E., Dunkel M., Preissner R. ProTox 3.0: A Webserver for the Prediction of Toxicity of Chemicals. Nucleic Acids Res (Web Server Issue 2024). https://tox.charite.de/protox3.

[34] Daina A., Michielin O., Zoete V. (2017). SwissADME: A free web tool to evaluate pharmacokinetics, drug-likeness and medicinal chemistry friendliness of small molecules. Sci. Rep..

[35] Liu Q., Tang Z., Surdenikova L., Kim S., Patel K.N., Kim A., Ru F., Guan Y., Weng H.-J., Geng Y. (2009). Sensory Neuron-Specific GPCR Mrgprs Are Itch Receptors Mediating Chloroquine-Induced Pruritus. Cell.

[36] Larson E.T., Ojo K.K., Murphy R.C., Johnson S.M., Zhang Z., Kim J.E., Leibly D.J., Fox A.M.W., Reid M.C., Dale E.J. (2012). Multiple Determinants for Selective Inhibition of Apicomplexan Calcium-Dependent Protein Kinase CDPK1. J. Med. Chem..

[37] Alberge B., Gannoun-Zaki L., Bascunana C., Tran van Ba C., Vial H., Cerdan R. (2009). Comparison of the cellular and biochemical properties of *Plasmodium falciparum* choline and ethanolamine kinases. Biochem. J..

[38] Zhang M.V., Chavchich M., Waters N.C. (2012). Targeting Protein Kinases in the Malaria Parasite: Update of an Antimalarial Drug Target. Curr. Top. Med. Chem..

[39] Doerig C., Abdi A., Bland N., Eschenlauer S., Dorin-Semblat D., Fennell C., Halbert J., Holland Z., Nivez M.-P., Semblat J.-P. (2010). Malaria: Targeting parasite and host cell kinomes. Biochim. Biophys. Acta.

[40] Cassiano G.C., Tavella T.A., Nascimento M.N., Rodrigues D.A., Cravo P.V.L., Andrade C.H., Costa F.T.M. (2021). Targeting malaria protein kinases. Advances in Protein Chemistry and Structural Biology.

[41] Doerig C., Meijer L. (2007). Antimalarial drug discovery: Targeting protein kinases. Expert Opin. Ther. Targets.

[42] Hughes T.B., Dang N.L., Miller G.P., Swamidass S.J. (2016). Modeling Reactivity to Biological Macromolecules with a Deep Multitask Network. ACS Cent. Sci..

[43] Hughes T.B., Miller G.P., Swamidass S.J. (2015). Site of reactivity models predict molecular reactivity of diverse chemicals with glutathione. Chem. Res. Toxicol..

[44] Le Dang N., Hughes T.B., Krishnamurthy V., Swamidass S.J. (2016). A Simple Model Predicts UGT-Mediated Metabolism. Bioinformatics.

[45] Schmid R., Heuckeroth S., Korf A. (2023). Integrative analysis of multimodal mass spectrometry data in MZmine 3. Nat Biotechnol..

[46] Morehouse N.J., Clark T.N., McMann E., van Santen J.A., Haeckl J.F.P., Gray C.A., Linington R.G. (2023). Annotations of Natural Product Compound Families Using Molecular Networking Topology and Structural Similarity Fingerprinting. Nat. Commun..

[47] Nothias L.-F., Petras D., Schmid R., Dührkop K., Rainer J., Sarvepalli A., Protsyuk I., Ernst M., Tsugawa H., Fleischauer M. (2020). Feature-based molecular networking in the GNPS analysis environment. Nat. Methods.

[48] Wang M., Carver J.J., Phelan V.V., Sanchez L.M., Garg N., Peng Y., Nguyen D.D., Watrous J., Kapono C.A., Luzzatto-Knaan T. (2016). Sharing and community curation of mass spectrometry data with Global Natural Products Social Molecular Networking. Nat. Biotechnol..

[49] Mohimani H., Gurevich A., Shlemov A., Mikheenko A., Korobeynikov A., Cao L., Shcherbin E., Nothias L.-F., Dorrestein P.C., Pevzner P.A. (2018). Dereplication of microbial metabolites through database search of mass spectra. Nat. Commun..

[50] Cao L., Guler M., Tagirdzhanov A., Lee Y.-Y., Gurevich A., Mohimani H. (2021). MolDiscovery: Learning mass spectrometry fragmentation of small molecules. Nat. Commun..

[51] Borelli T.C., Arini G.S., Feitosa L.G.P., Dorrestein P.C., Lopes N.P., Da Silva R.R. (2023). Improving annotation propagation on molecular networks through random walks: Introducing ChemWalker. Bioinformatics.

[52] Trager W., Jensen J.B. (2005). Human Malaria Parasites in Continuous Culture. J. Parasitol..

