# Supplementary material for: Bioactive Cyclopeptide Alkaloids and Ceanothane Triterpenoids from Ziziphus mauritiana Roots: Antiplasmodial Activity, UHPLC-MS/MS Molecular Networking, ADMET Profiling, and Target Prediction
Source: Molecules. 2025 Jul 14;30(14):2958. doi: 10.3390/molecules30142958 (PMC12299143; doi:10.3390/molecules30142958)
Supplement: Supplementary file 1 [file molecules-30-02958-s001.zip › molecules-3687167-supplementary.pdf]

Supporting information

Bioactive Cyclopeptide Alkaloids and Ceanothane Triterpenoids from *Ziziphus mauritiana* Roots: Antiplasmodial Activity, UHPLC-MS/MS Molecular Networking, ADMET Profiling, and Target Prediction

Sylvestre Saidou Tsila [1,2], Mc Jesus Kinyok [2], Joseph Eric Mbasso Tameko [2], Bel Youssouf G. Mountessou [2], Kevine Johanne Jumeta Dongmo [1,2], Jean Koffi Garba [3], Noella Molisa Efange [4,5], Lawrence Ayong [5], Yannick Stéphane Fotsing Fongang [6,*], Jean Jules Kezetas Bankeu [2], Norbert Sewald [7] and Bruno Ndjakou Lenta [2,7,*]

[1] Department of Organic Chemistry, Faculty of Science, University of Yaoundé I, Yaoundé P.O. Box 812, Cameroon; saidoutsila@yahoo.fr (S.S.T.); jumetakevine@yahoo.fr (K.J.J.D.)
[2] Department of Chemistry, Higher Teacher Training College, University of Yaoundé I, Yaoundé P.O. Box 47, Cameroon; mcjesuskinyok@yahoo.fr (M.J.K.); tamekombasso@yahoo.fr (J.E.M.T.); mountessou@yahoo.com (B.Y.G.M.); bk_jeanjules@yahoo.fr (J.J.K.B.)
[3] Department of Basic Science Education, National Advanced School of Maritime and Ocean Science and Technology, University of Ebolowa, Kribi P.O. Box 292, Cameroon; garbakoffijean@yahoo.fr
[4] Department of Biochemistry and Molecular Biology, Faculty of Science, The University of Buea, Buea P.O. Box 63, Cameroon; noella.efange@yahoo.com
[5] Malaria Research Service, Centre Pasteur du Cameroun, Yaoundé P.O. Box 1274, Cameroon; ayong@pasteur-yaounde.org
[6] Department of Chemistry, Higher Teachers' Training College, The University of Maroua, Maroua P.O. Box 55, Cameroon
[7] Organic and Bioorganic Chemistry, Faculty of Chemistry, Bielefeld University, D-33501 Bielefeld, Germany; norbert.sewald@uni-bielefeld.de
* Correspondence: fongangfys@yahoo.fr (Y.S.F.F.); bruno.lenta_ndjakou@uni-bielefeld.de or lentabruno@yahoo.fr (B.N.L.)

Table of contents

Figure S1: (+) HRESIMS spectrum of compound **1** (*m/z* 592) 5

Figure S2: (+) ESI MS/MS spectrum of compound **1** (*m/z* 592) 5

Figure S3: (+) HR-ESI-MS spectrum of compound **2** (*m/z* 491) 5

Figure S4: (+) HR-ESI-MS/MS spectrum of compound **2** (*m/z* 491) 6

Figure S5: (+) HRESIMS spectrum of compound **3** (*m/z* 505) 6

Figure S6: (+) ESI MS/MS spectrum of compound **3** (*m/z* 505) 7

Figure S7: (+) HRESIMS spectrum of compound **4** (*m/z* 562) 7

Figure S8: (+) ESI MS/MS spectrum of compound **4** (*m/z* 562) 8

Figure S9: (+) HR-ESI-MS spectrum of compound **5** (*m/z* 576) 9
Figure S10: (+) HR-ESI-MS/MS spectrum of compound **5** (*m/z* 576) 9
Figure S11: (+) HR-ESI-MS spectrum of compound **6** (*m/z* 608) 10
Figure S12: (+) HR-ESI-MS/MS spectrum of compound **6** (*m/z* 608) 10
Figure S13: (+) HRESIMS of compound **7** (*m/z* 592) 11
Figure S14: (+) HRESIMS/MS of compound **7** (*m/z* 592) 11
Figure S15: (+) HRESIMS spectrum of compound **8** (*m/z* 574) 12
Figure S16: (+) HRESI MS/MS spectrum of compound **8** (*m/z* 574) 12
Figure S17: (+) HRESIMS/MS 9 (*m/z* 590) 13
Figure S18: (+) ESIMS/MS **9** (*m/z* 590) 13
Figure S19: (+) HRESIMS spectrum of compound **10** (*m/z* 558) 13
Figure S20: (+) ESI MS/MS spectrum of compound **10** (*m/z* 558) 14
Figure S21: (+) HRESIMS spectrum of compound **11** (*m/z* 622) 14
Figure S22: (+) ESI MS/MS spectrum of compound **11** (*m/z* 622) 15
Figure S23: (+) ESI MS/MS spectrum of compound **12** (*m/z* 632) 15
Figure S24: (+) ESI MS/MS spectrum of compound **12** (*m/z* 632) 16
Figure S25: (+) HRESIMS/MS of compound **13** (*m/z* 535) 16
Figure S26: (+) HRESIMS/MS compound **13** (*m/z* 535) 17
Figure S27: (+) HRESIMS/MS of compound **14** (*m/z* 574) 17
Figure S29: (+) ESI MS/MS spectrum of compound **15** (*m/z* 592) 18
Figure S30: (+) ESI MS/MS spectrum of compound **15** (*m/z* 592) 19
Figure S31: (+) HRESIMS of compound **16** (*m/z* 606) 20
Figure S32: (+) HRESIMS/MS of compound **16** (*m/z* 606) 20
Figure S33: (+) HRESIMS spectrum of compound **17** (*m/z* 657) 20
Figure S34: (+) ESI MS/MS spectrum of compound **17** (*m/z* 657) 21
Figure S35: (+) HRESIMS spectrum of compound **18** (*m/z* 632) 21
Figure S36: (+) ESI MS/MS spectrum of compound **18** (*m/z* 632) 22
Figure S37: (+) HRESIMS of compound **19** (*m/z* 671) 22
Figure S38: (+) HRESIMS/MS of compound **19** (*m/z* 671) 23
Figure S39: (+) HRESIMS spectrum of compound **20** (*m/z* 620) 23
Figure S40: (+) ESI MS/MS spectrum of compound **20** (*m/z* 620) 24
Figure S41: (+) HRESIMS of compound **21** (*m/z* 588) 24
Figure S42: (+) HRESIMS/MS of compound **21** (*m/z* 588) 25

Figure S43: (+) HRESIMS/MS of compound **22** (*m/z* 606) 25
Figure S44: (+) HRESIMS/MS of compound **22** (*m/z* 606) 26
Figure S45: (+) HRESIMS spectrum of compound **23** (*m/z* 662) 27
Figure S46: (+) ESI MS/MS spectrum of compound **23** (*m/z* 662) 27
Figure S47: (+) HRESIMS of compound **24** (*m/z* 687) 27
Figure S48: (+) HRESIMS/MS of compound **24** (*m/z* 687) 28
Figure S49: (+) HRESIMS spectrum of compound **25** (*m/z* 701) 28
Figure S50: (+) ESI MS/MS spectrum of compound **25** (*m/z* 701) 29
Figure S51: (+) HRESIMS spectrum of compound **26** (*m/z* 501) 30
Figure S52: (+) HRESIMS spectrum of compound **27** (*m/z* 485) 30
Figure S53: (+) HRESIMS spectrum of compound **28** (*m/z* 469) 31
Figure S54: (+) HRESIMS spectrum of compound **29** (*m/z* 453) 31
Figure S55: (+) HRESIMS spectrum of compound **30** (*m/z* 455) 32
Figure S56: (+) HRESIMS spectrum of compound **31** (*m/z* 453) 32
Figure S57: (+) HRESIMS spectrum of compound **32** (*m/z* 451) 32
Figure S58: ^{1}HNMR spectrum ($CDCl_3$, 600 MHz) of compound **5** 33
Figure S59: ^{13}CNMR spectrum ($CDCl_3$, 150 MHz) of compound **5** 34
Figure S60: ^{1}HNMR spectrum ($CDCl_3$+ CD_3OD, 600 MHz) of compound **6** 34
Figure S61: ^{13}CNMR spectrum ($CDCl_3$ + CD_3OD, 150 MHz) of compound **6** 35
Figure S62: ^{1}HNMR spectrum ($CDCl_3$+ CD_3OD, 600 MHz) of compound **14** 35
Figure S63: ^{13}CNMR spectrum ($CDCl_3$+ CD_3OD, 150 MHz) of compound **14** 35
Figure S64: ^{1}H NMR spectrum (CD_3OD, 600 MHz) of compound **26** 36
Figure S65: ^{13}CNMR spectrum (CD_3OD, 150 MHz) of compound **26** 36
Figure S66: ^{1}H NMR spectrum ($CDCl_3$ / CD_3OD, 600MHz) of compound **27** 37
Figure S67: ^{13}C NMR spectrum ($CDCl_3$ / CD_3OD, 150 MHz) of compound **27** 37
Figure S68: ^{1}HNMR spectrum (CD_3OD, 600 MHz) of compound **28** 38
Figure S69: ^{13}CNMR spectrum (CD_3OD, 600 MHz) of compound **28** 38
Figure S70: ^{1}HNMR spectrum (DMSO-d_6, 600 MHz) of compound **29** 39
Figure S71: ^{13}CNMR spectrum of (DMSO-d_6, 150 MHz) of compound **29** 39
Figure S72: ^{1}H NMR spectrum ($CDCl_3$ / CD_3OD, 600 MHz) of **30** 40
Figure S73: ^{13}C NMR spectrum ($CDCl_3$ / CD_3OD, 150 MHz) of compound **30** 40
Figure S74: ^{1}H NMR spectrum ($CDCl_3$, 600 MHz) of compound **32** 41
Figure S75: ^{13}C NMR spectrum ($CDCl_3$, 150 MHz) of compound **32** 41

Figure S76: ^{1}H NMR spectrum ($CDCl_3$, 500 MHz) of compound **33** 42

Figure S77: ^{13}C NMR spectrum ($CDCl_3$, 125 MHz) of compound **33**.................................... 42

Figure S78: ^{1}H NMR spectrum ($CDCl_3$, 600 MHz) of compound **34** and **35** 43

Figure S79: ^{13}C NMR spectrum ($CDCl_3$, 600 MHz) of compound **34** and **35** 43

Figure S80: ^{1}HNMR spectrum (DMSO-d_6, 600 MHz) of compound **36** 44

Figure S81: ^{13}CNMR spectrum (DMSO-d_6, 600 MHz) of compound **36** 44

Figure S82: Calibration curve of mauritine A (**5**) using BPC 45

Figure S83: Calibration curve of mauritine A (**5**) using UV (λ=254 *n*M)............................... 46

Figure S84: Pie chart of molecular targets of Artemisinin 46

Figure S85: Pie chart of molecular targets of Chloroquine 46

Figure S86: Calibration curve of amphibine A (**14**) using EIC 47

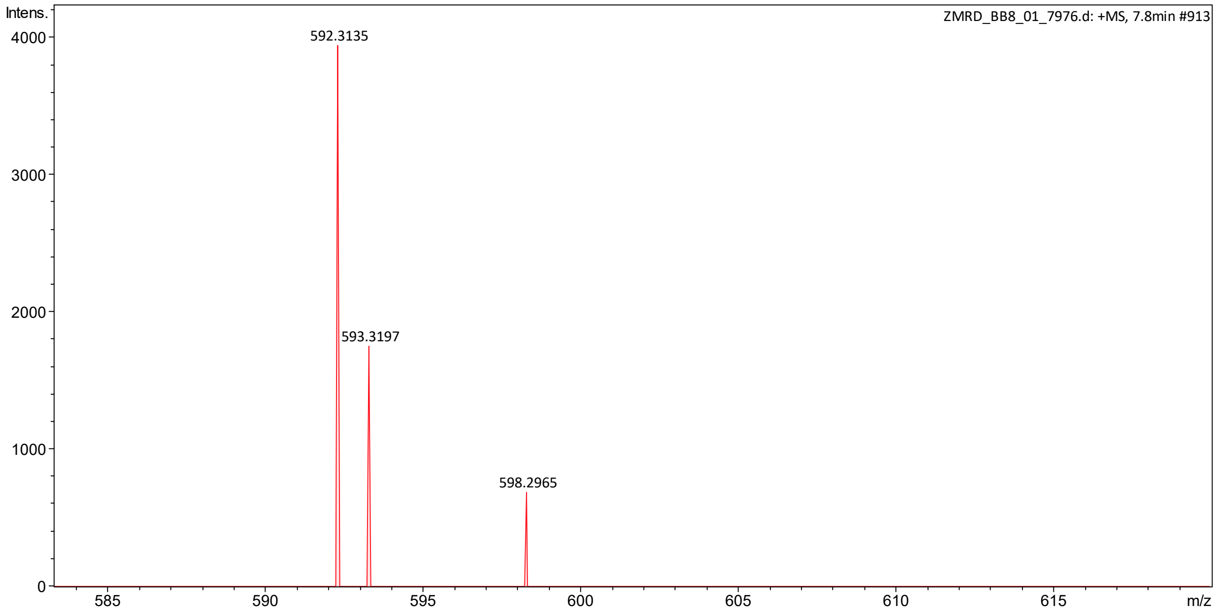

Figure S1: (+) HRESIMS spectrum of compound **1** (*m/z* 592)

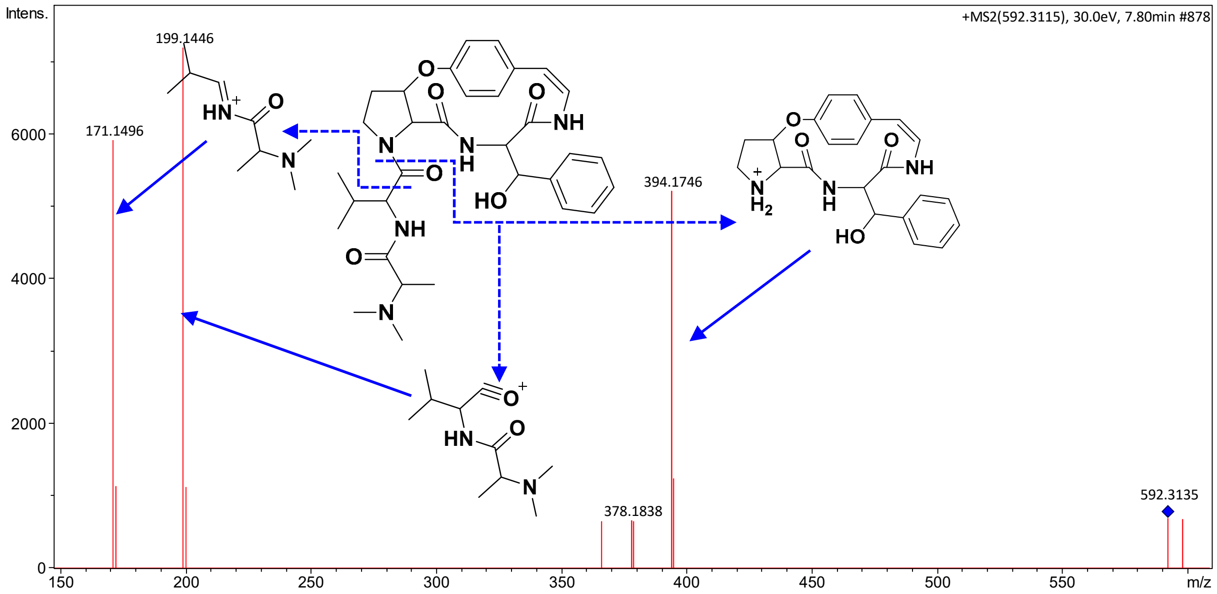

Figure S2: (+) ESI MS/MS spectrum of compound **1** (*m/z* 592)

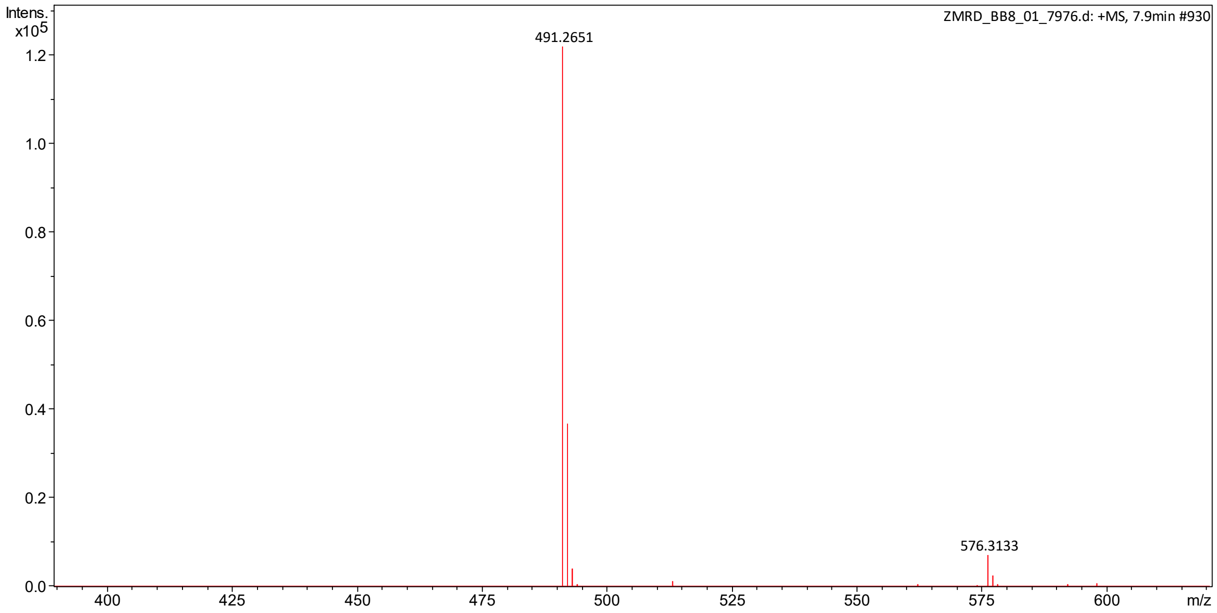

Figure S3: (+) HR-ESI-MS spectrum of compound **2** (*m/z* 491)

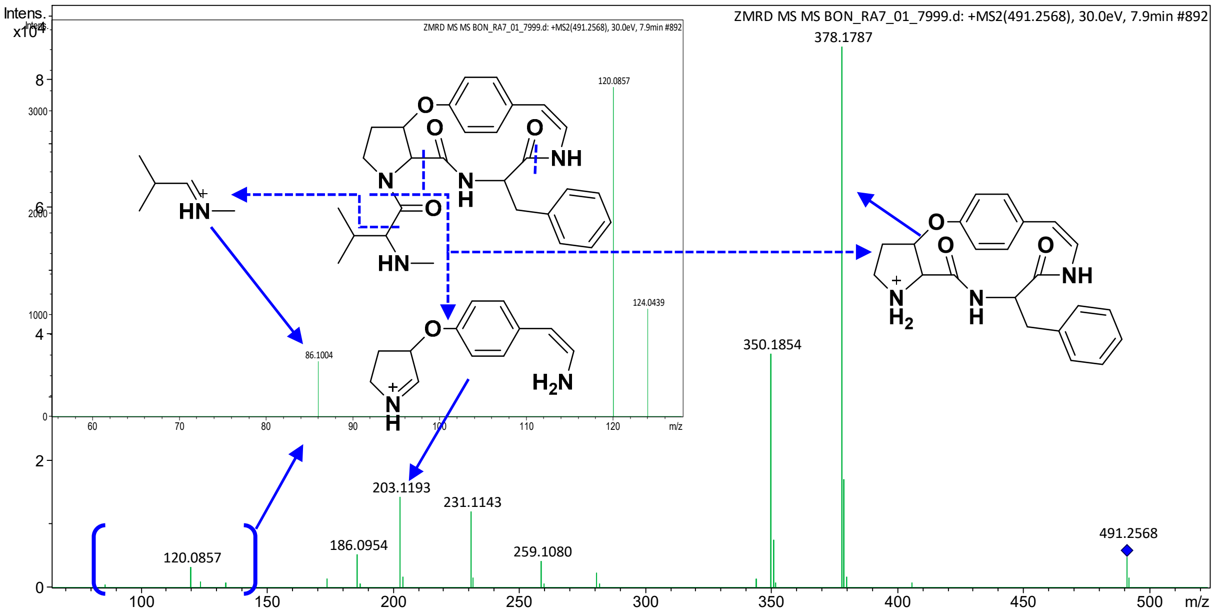

Figure S4: (+) HR-ESI-MS/MS spectrum of compound **2** (*m/z* 491)

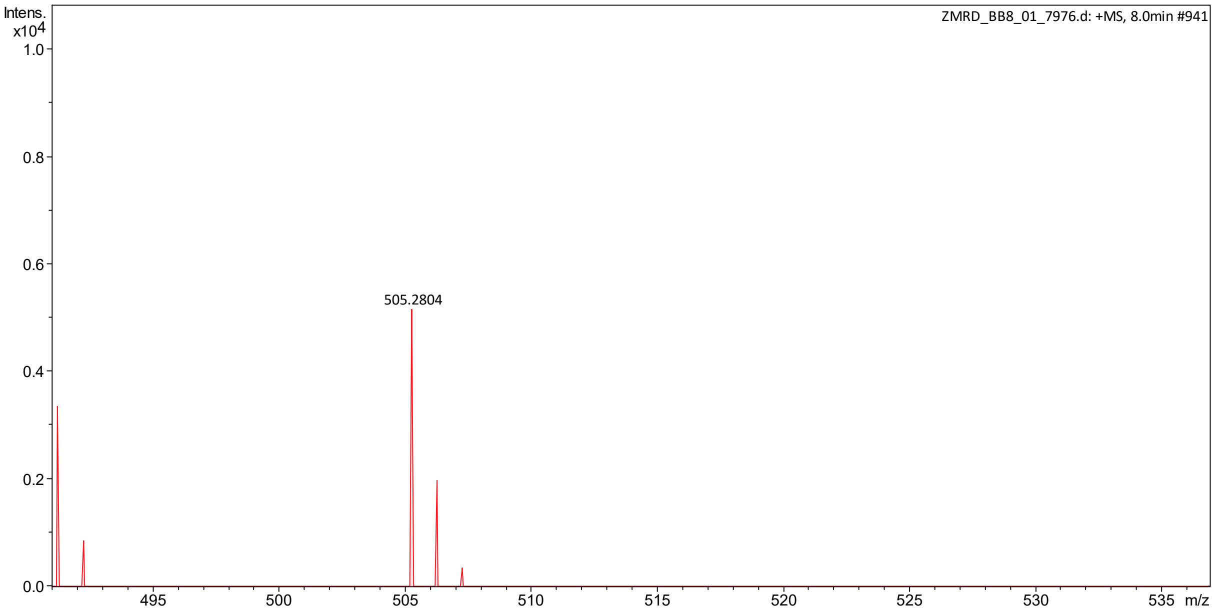

Figure S5: (+) HRESIMS spectrum of compound **3** (*m/z* 505)

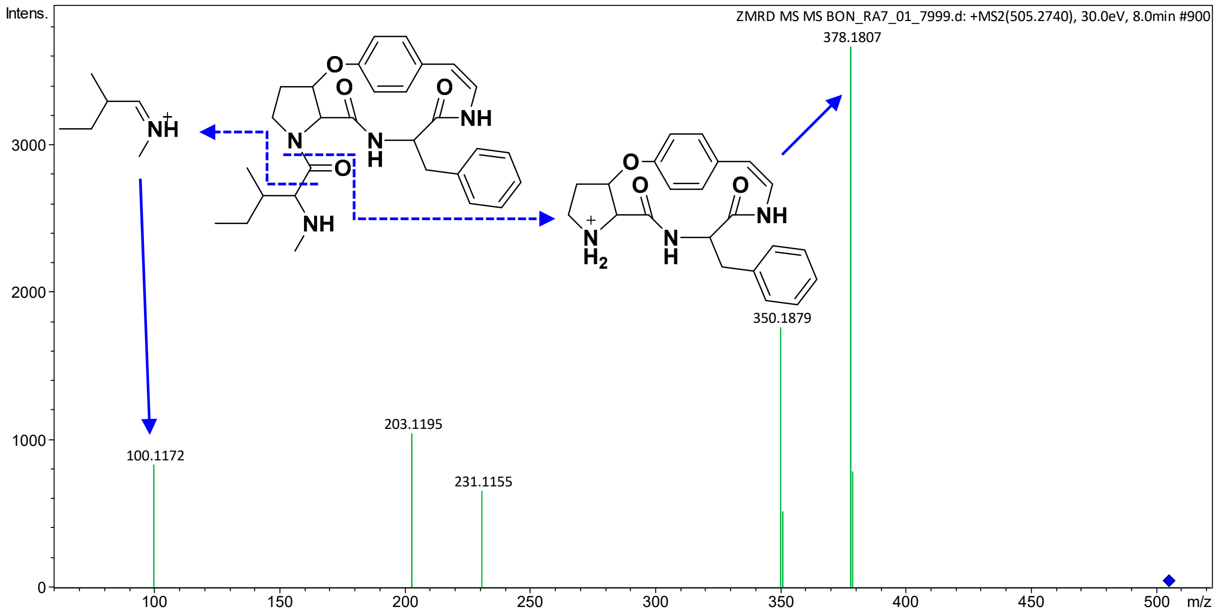

Figure S6: (+) ESI MS/MS spectrum of compound **3** (*m/z* 505)

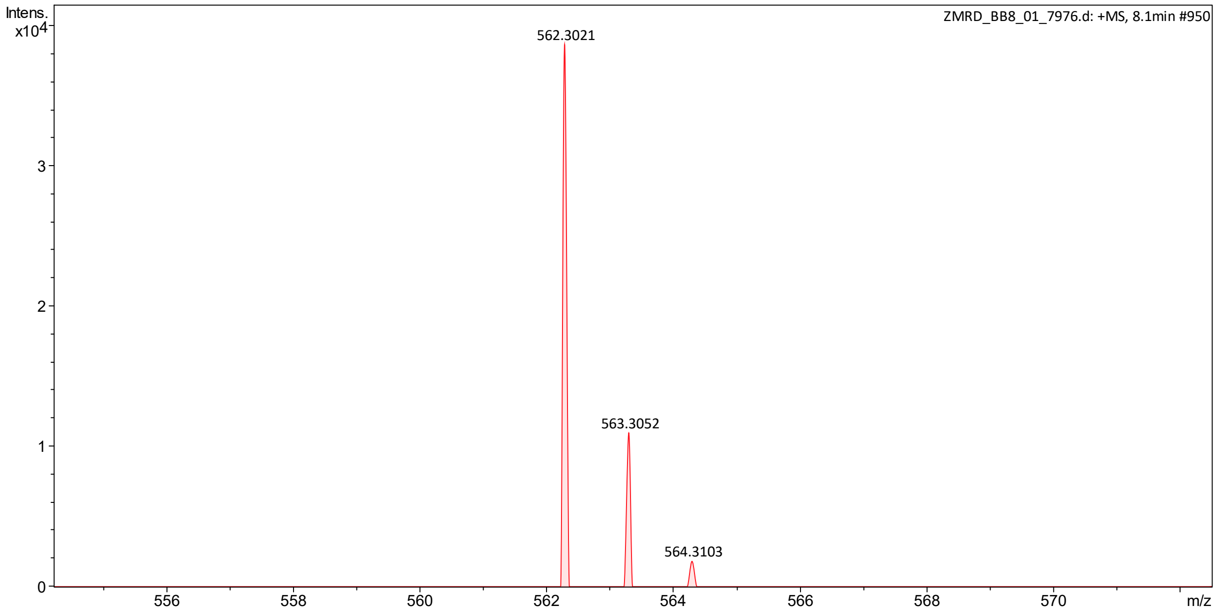

Figure S7: (+) HRESIMS spectrum of compound **4** (*m/z* 562)

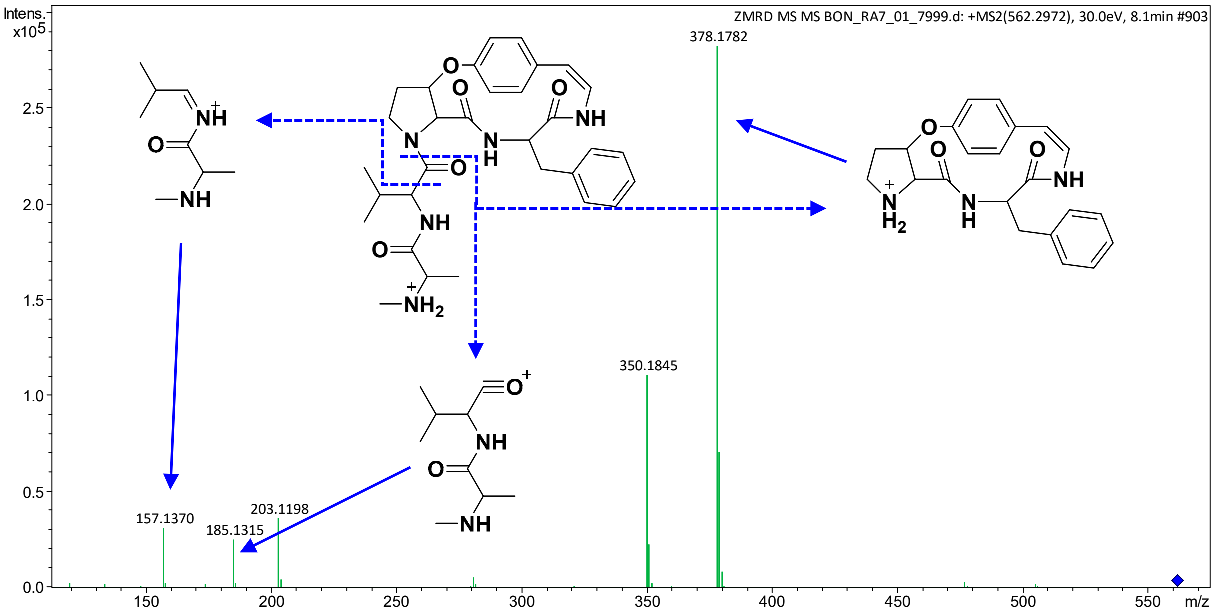

Figure S8: (+) ESI MS/MS spectrum of compound **4** (*m/z* 562)

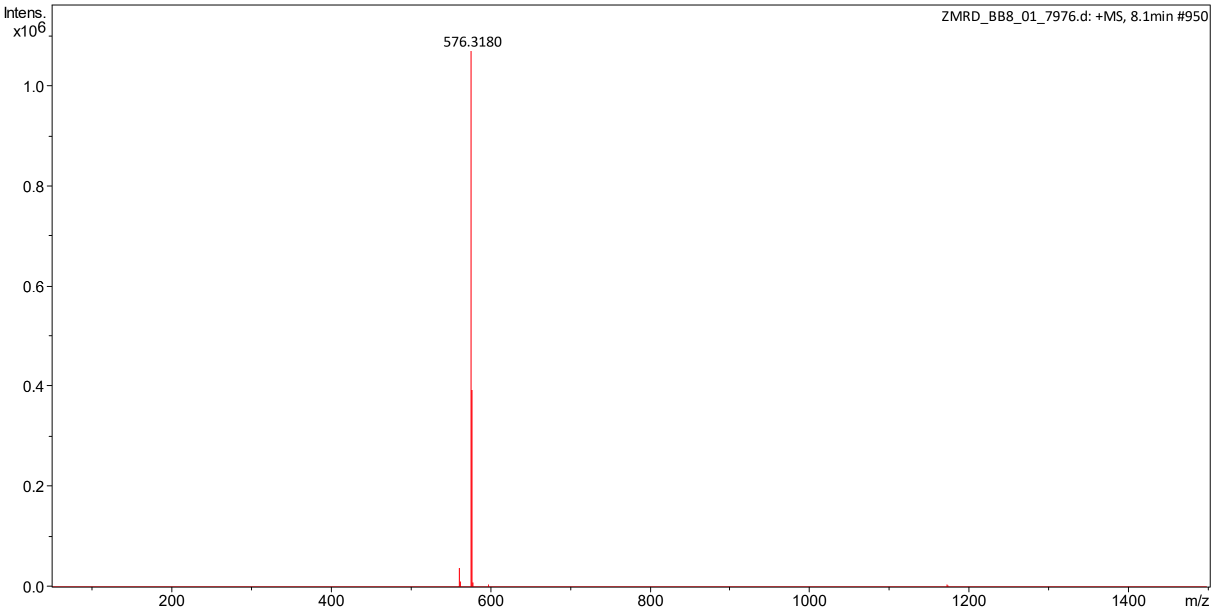

Figure S9: (+) HR-ESI-MS spectrum of compound **5** (*m/z* 576)

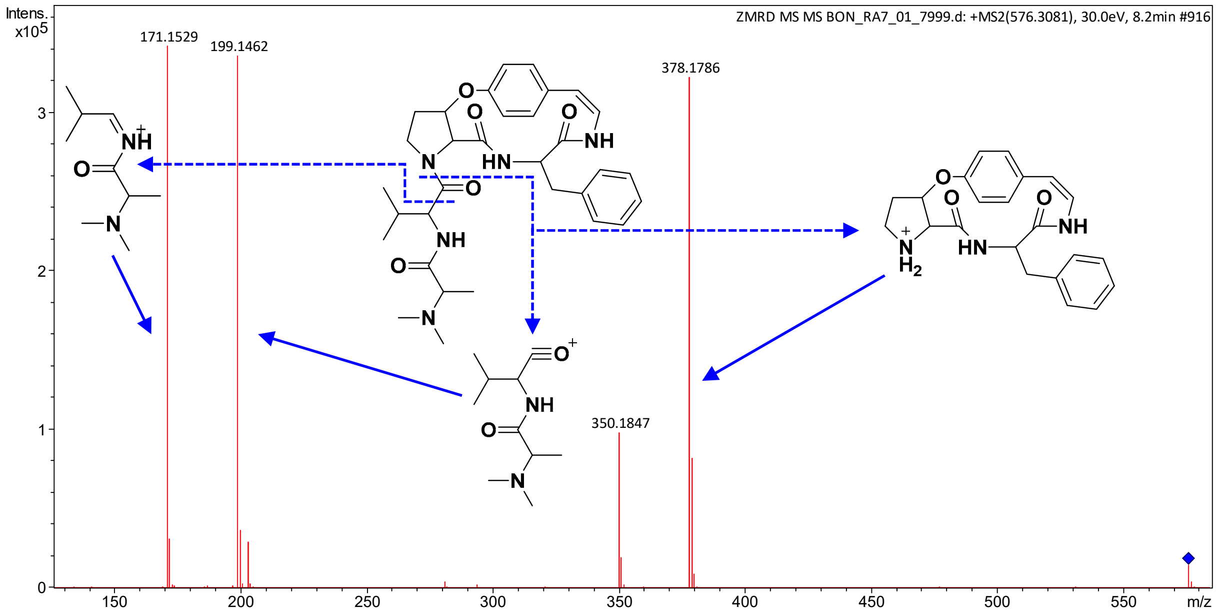

Figure S10: (+) HR-ESI-MS/MS spectrum of compound **5** (*m/z* 576)

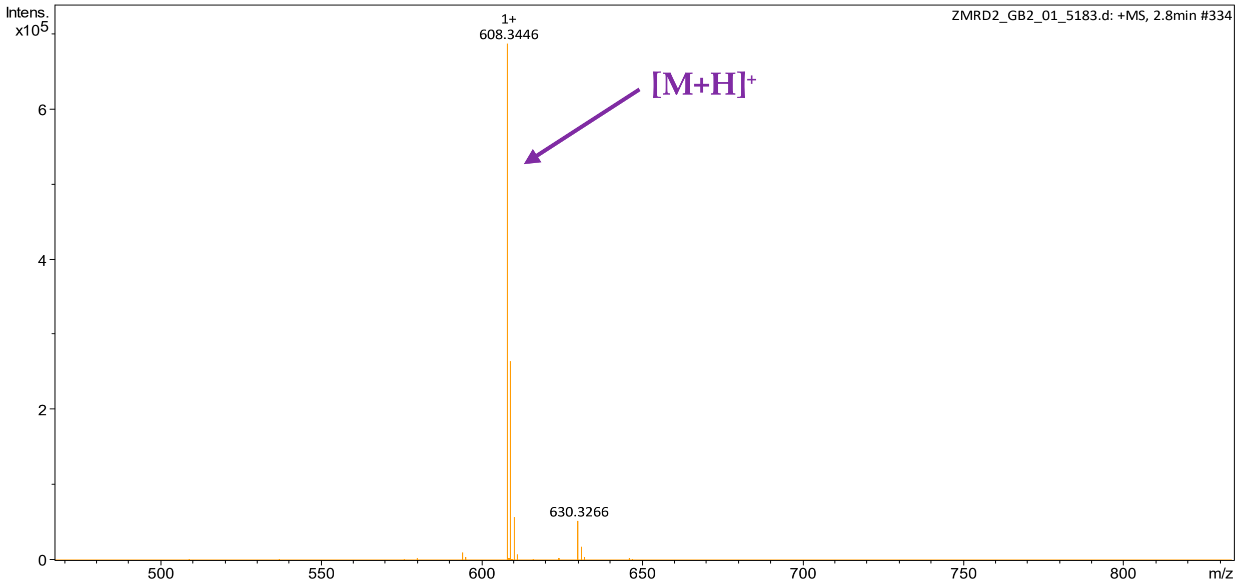

Figure S11: (+) HR-ESI-MS spectrum of compound **6** (*m/z* 608)

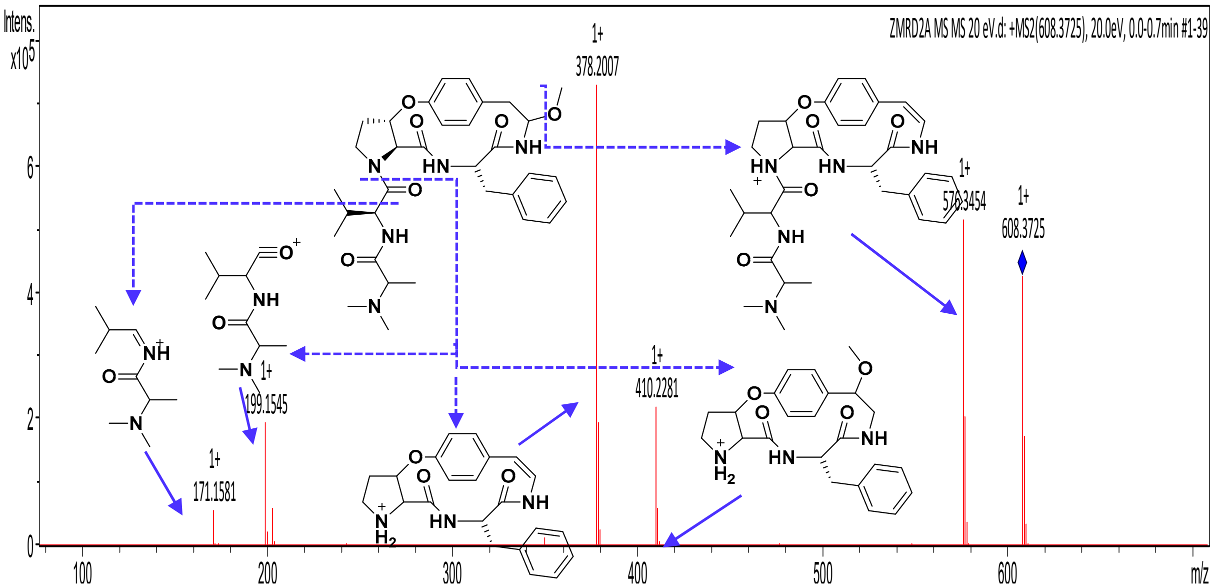

Figure S12: (+) HR-ESI-MS/MS spectrum of compound **6** (*m/z* 608)

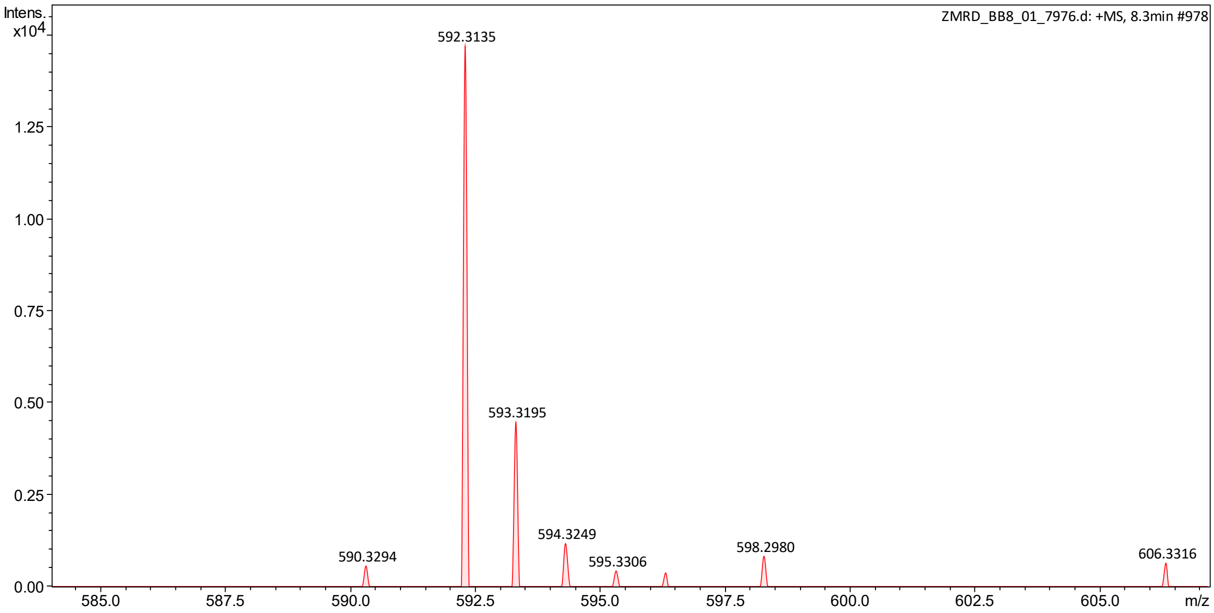

Figure S13: (+) HRESIMS of compound **7** (*m/z* 592)

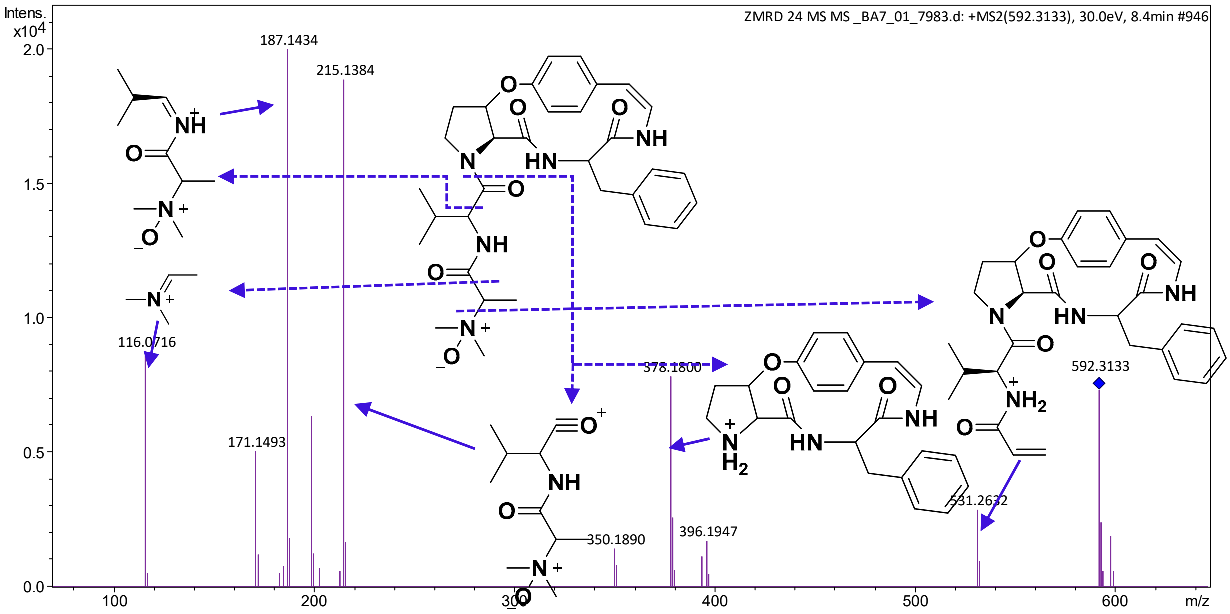

Figure S14: (+) HRESIMS/MS of compound **7** (*m/z* 592)

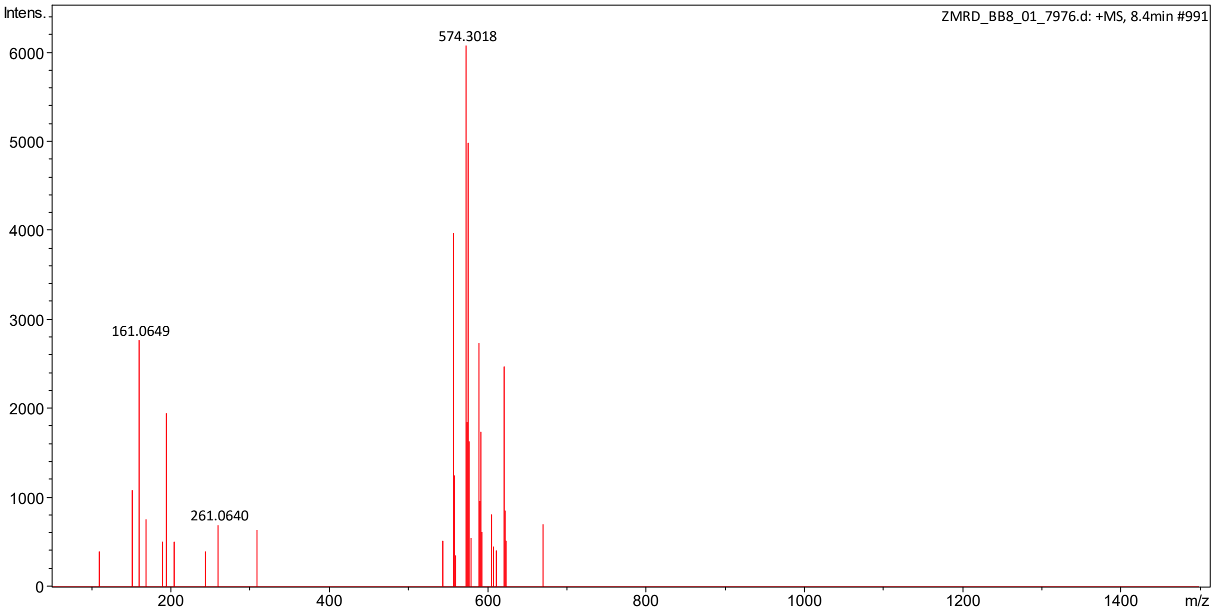

Figure S15: (+) HRESIMS spectrum of compound **8** (*m/z* 574)

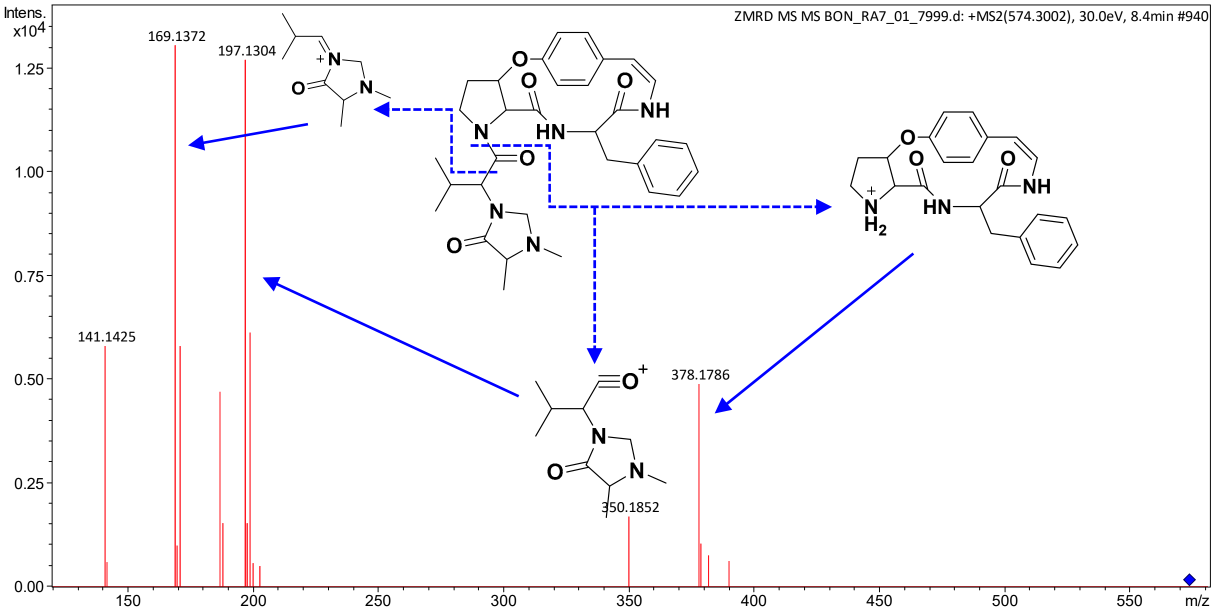

Figure S16: (+) HRESI MS/MS spectrum of compound **8** (*m/z* 574)

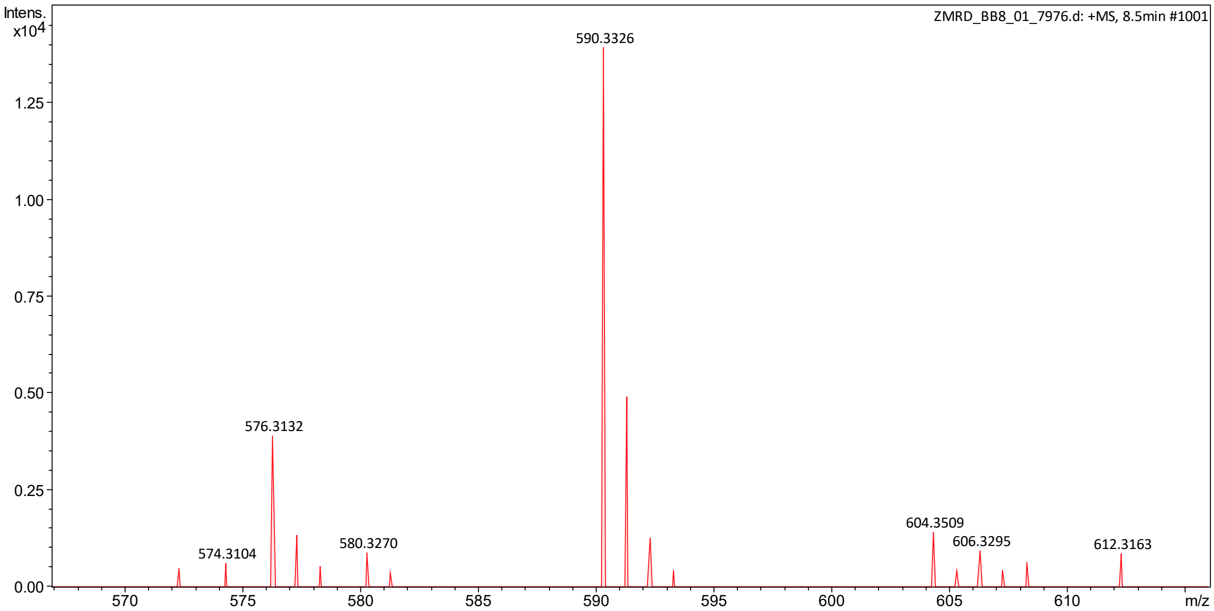

Figure S17: (+) HRESIMS/MS 9 (*m*/*z* 590)

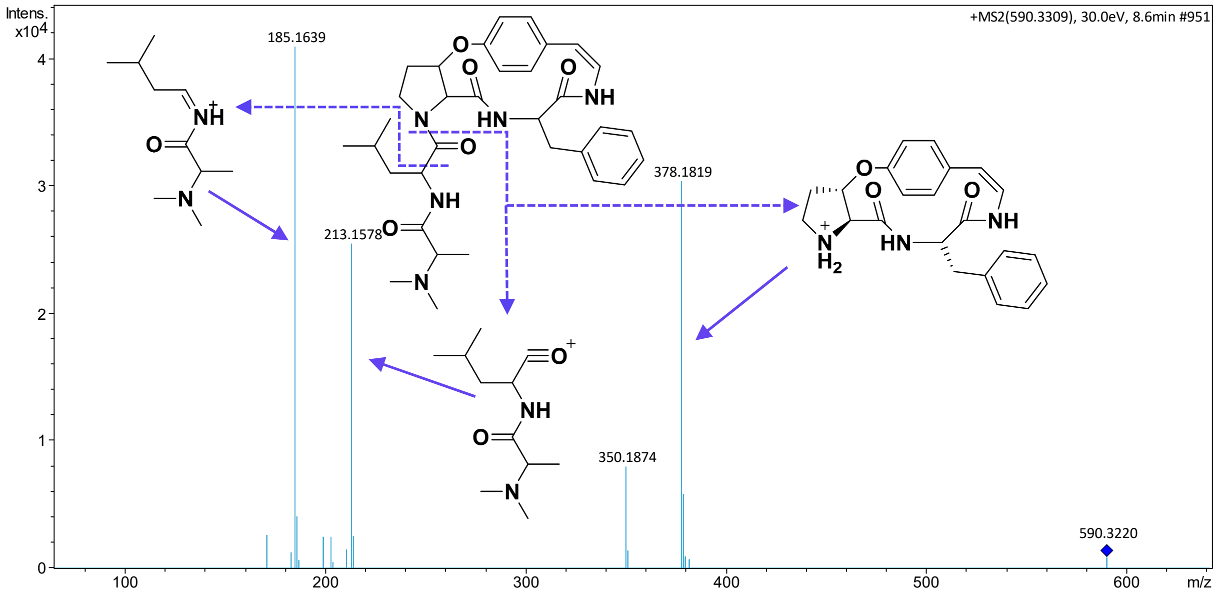

Figure S18: (+) ESIMS/MS **9** (*m*/*z* 590)

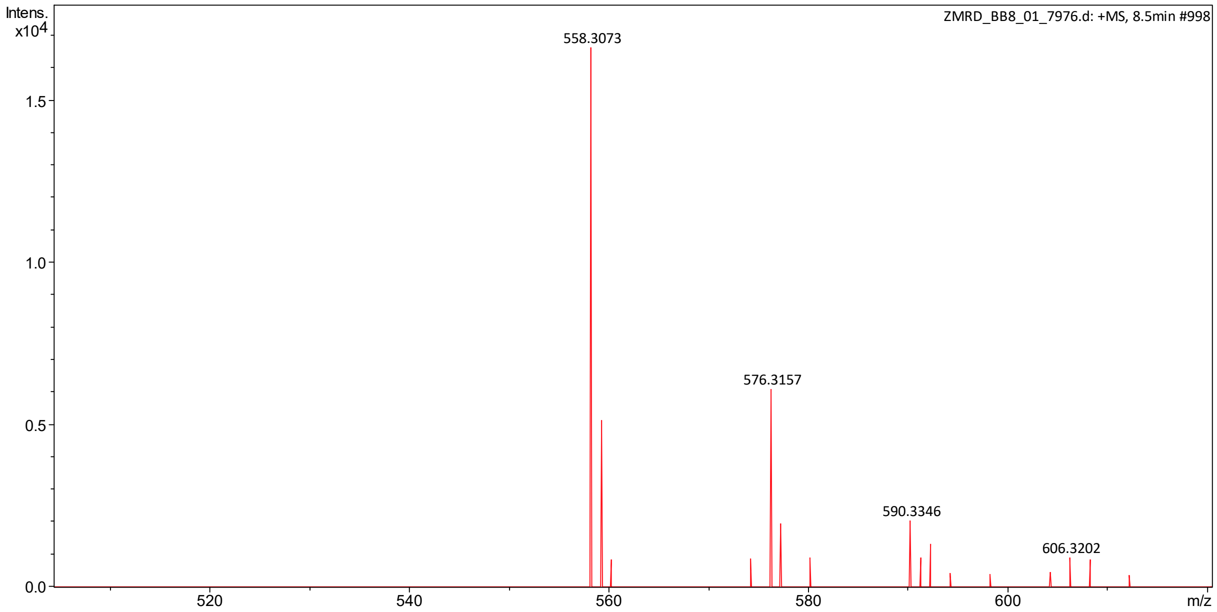

Figure S19: (+) HRESIMS spectrum of compound **10** (*m*/*z* 558)

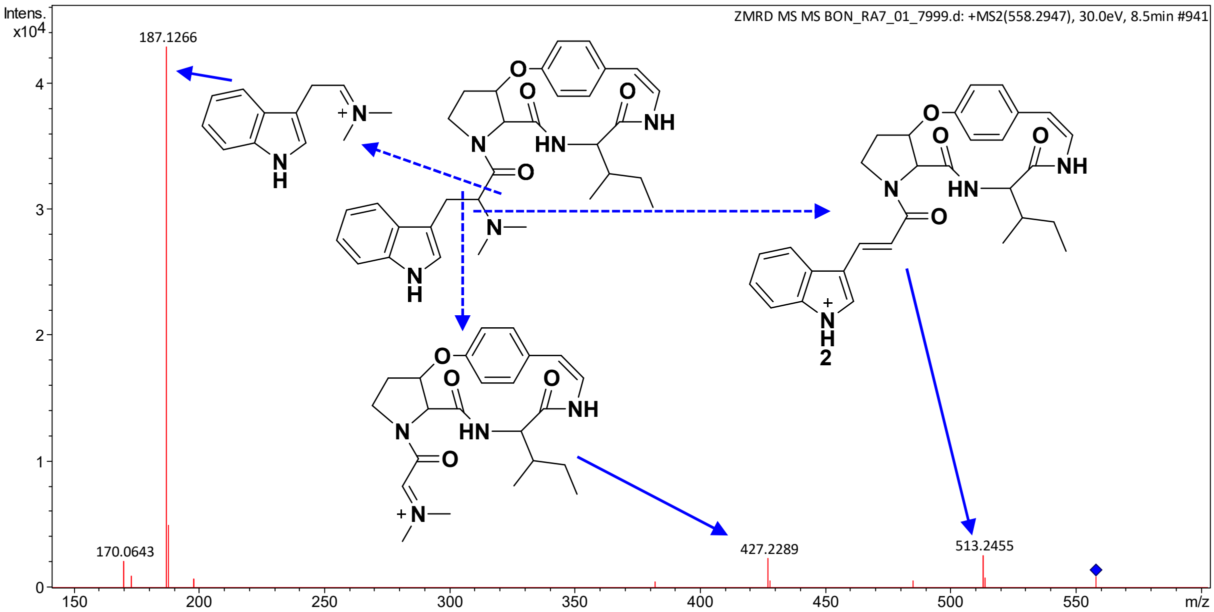

Figure S20: (+) ESI MS/MS spectrum of compound **10** (*m/z* 558)

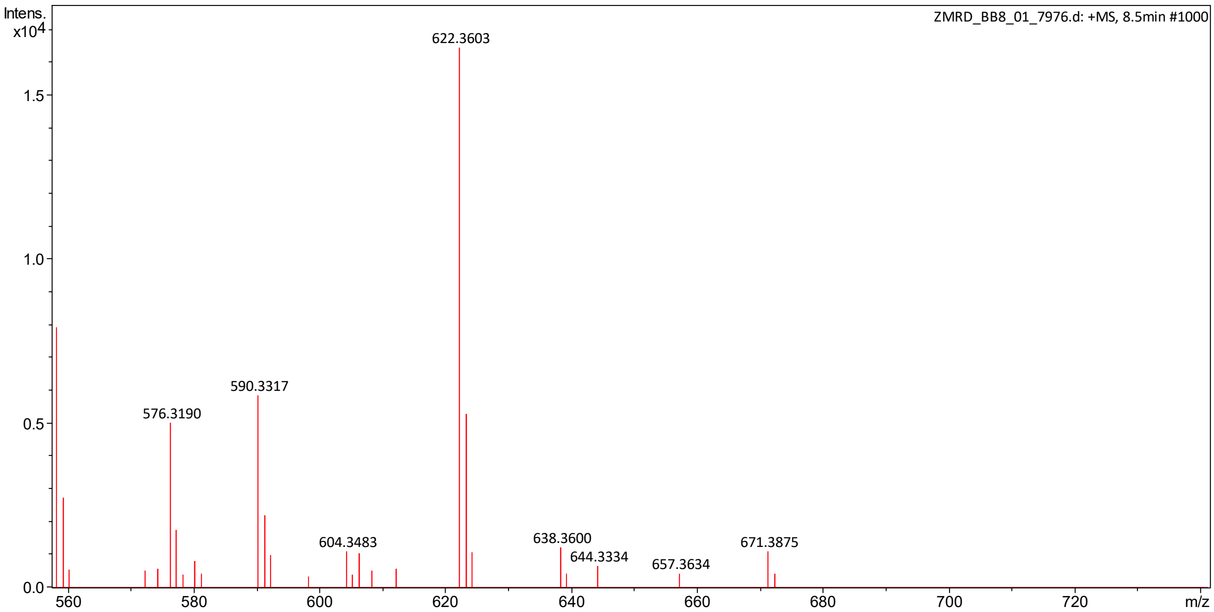

Figure S21: (+) HRESIMS spectrum of compound **11** (*m/z* 622)

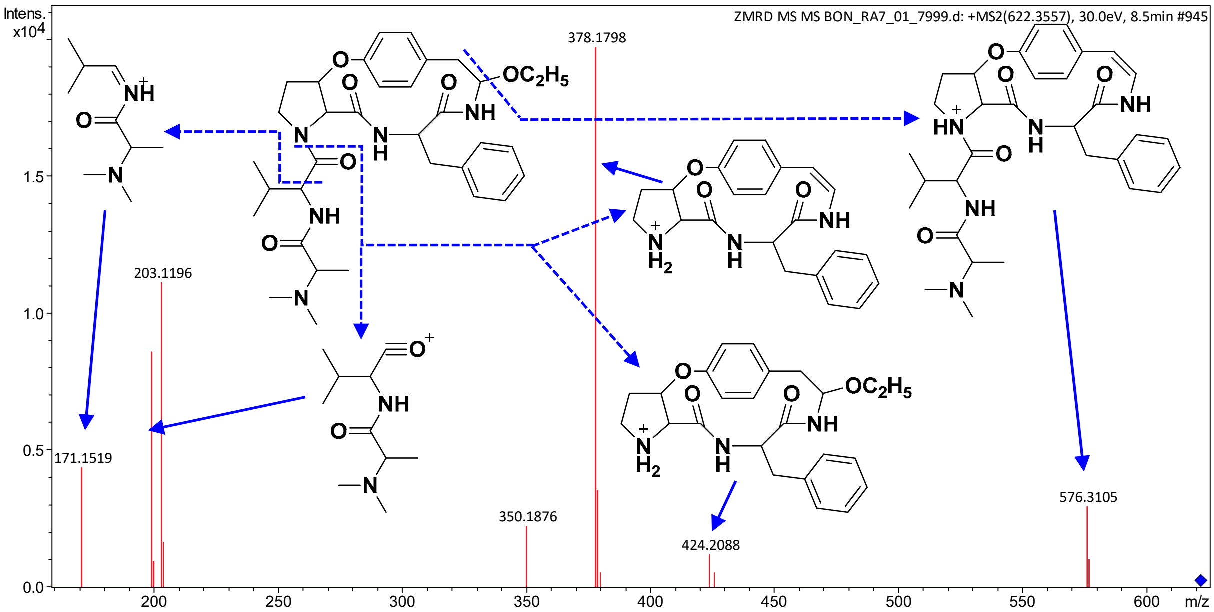

Figure S22: (+) ESI MS/MS spectrum of compound **11** (*m/z* 622)

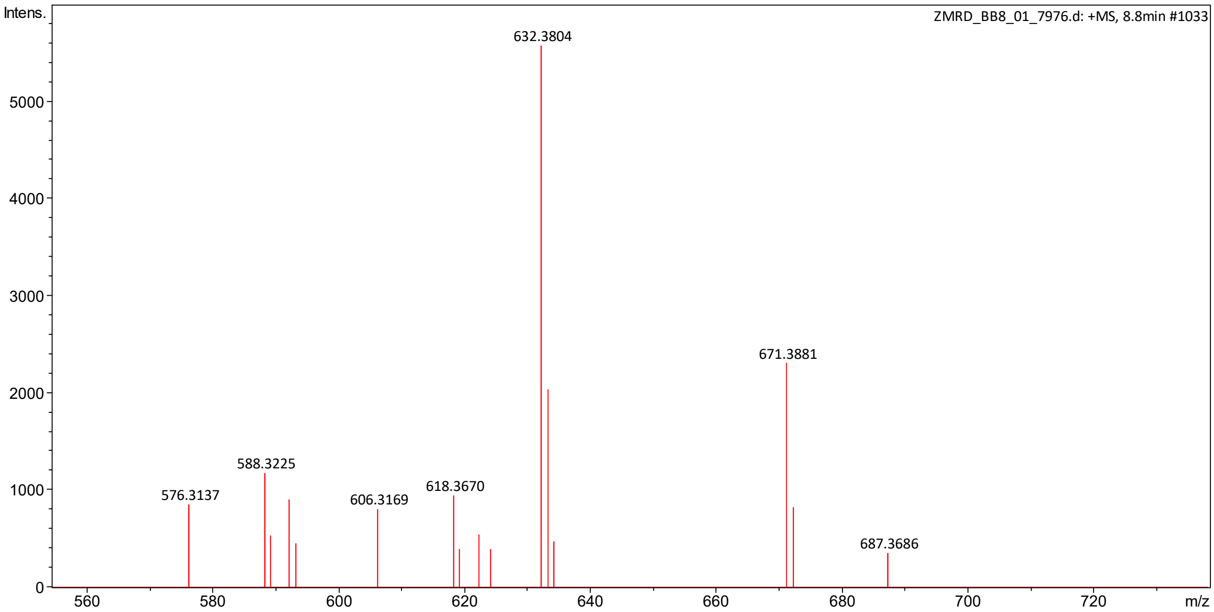

Figure S23: (+) ESI MS/MS spectrum of compound **12** (*m/z* 632)

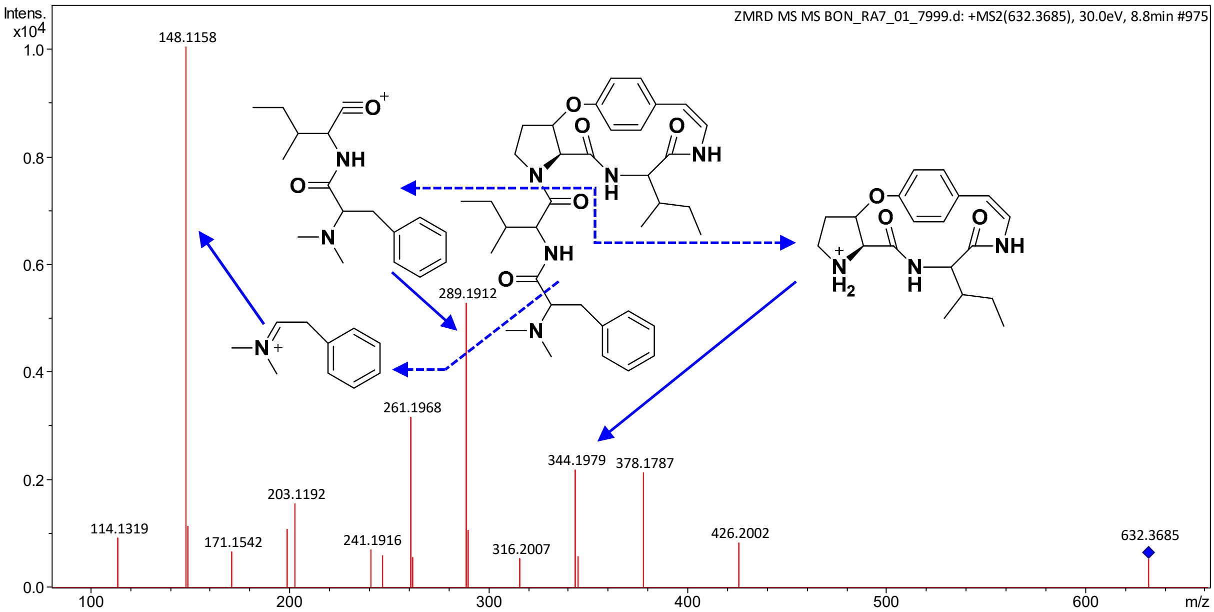

Figure S24: (+) ESI MS/MS spectrum of compound **12** (*m/z* 632)

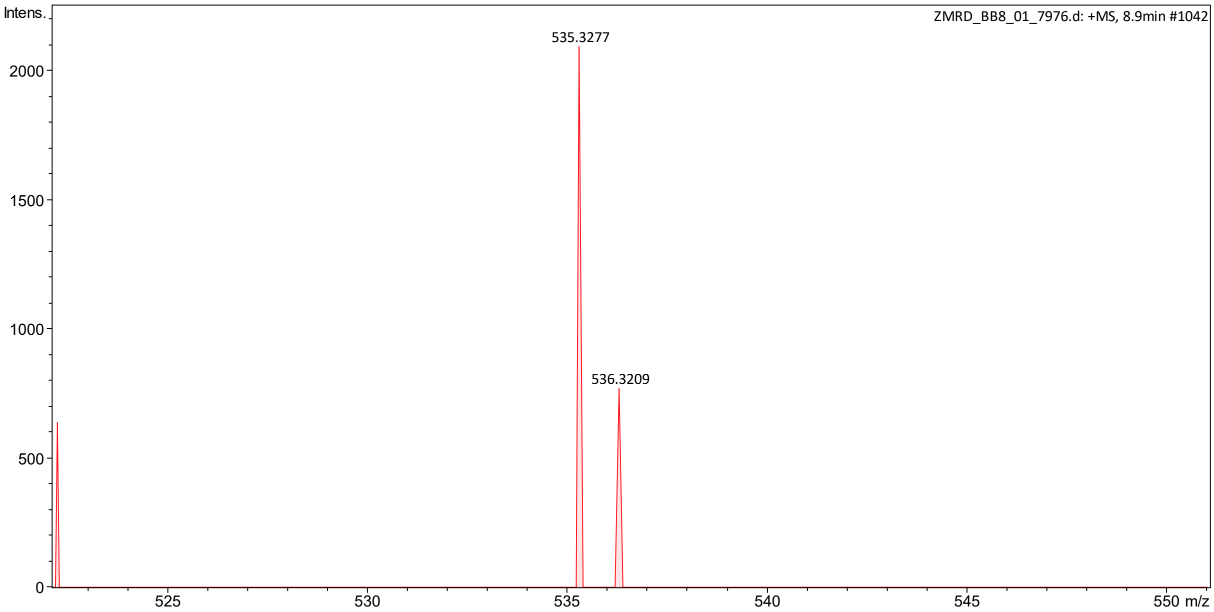

Figure S25: (+) HRESIMS/MS of compound **13** (*m/z* 535)

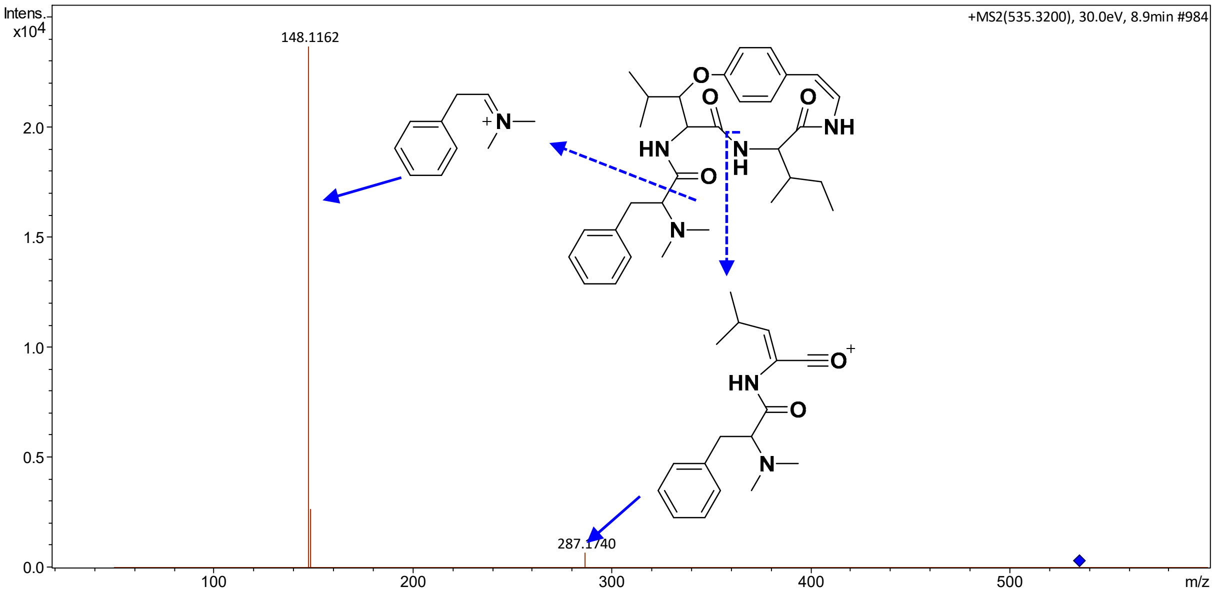

Figure S26: (+) HRESIMS/MS compound **13** (*m/z* 535)

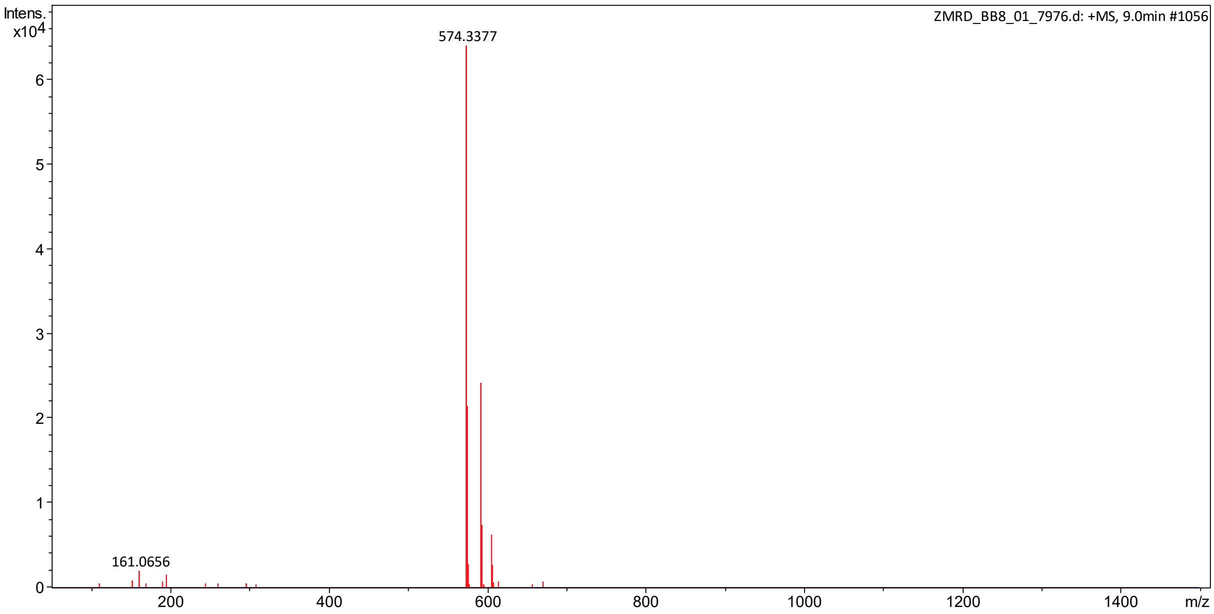

Figure S27: (+) HRESIMS/MS of compound **14** (*m/z* 574)

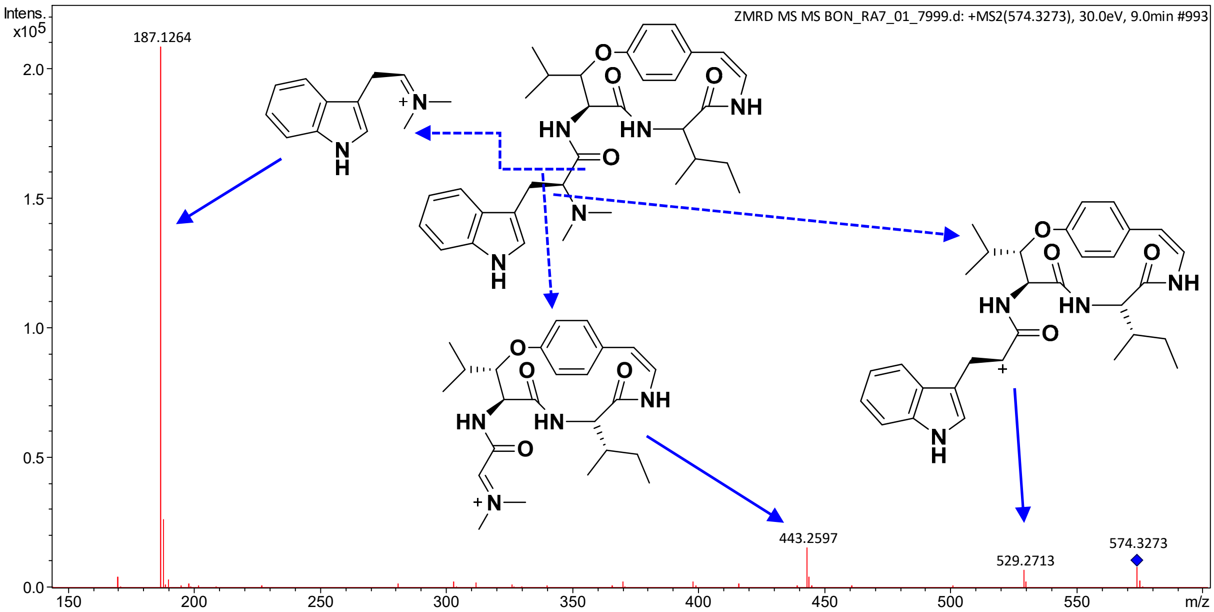

Figure S28: (+) HRESIMS/MS of compound **14** (*m/z* 574)

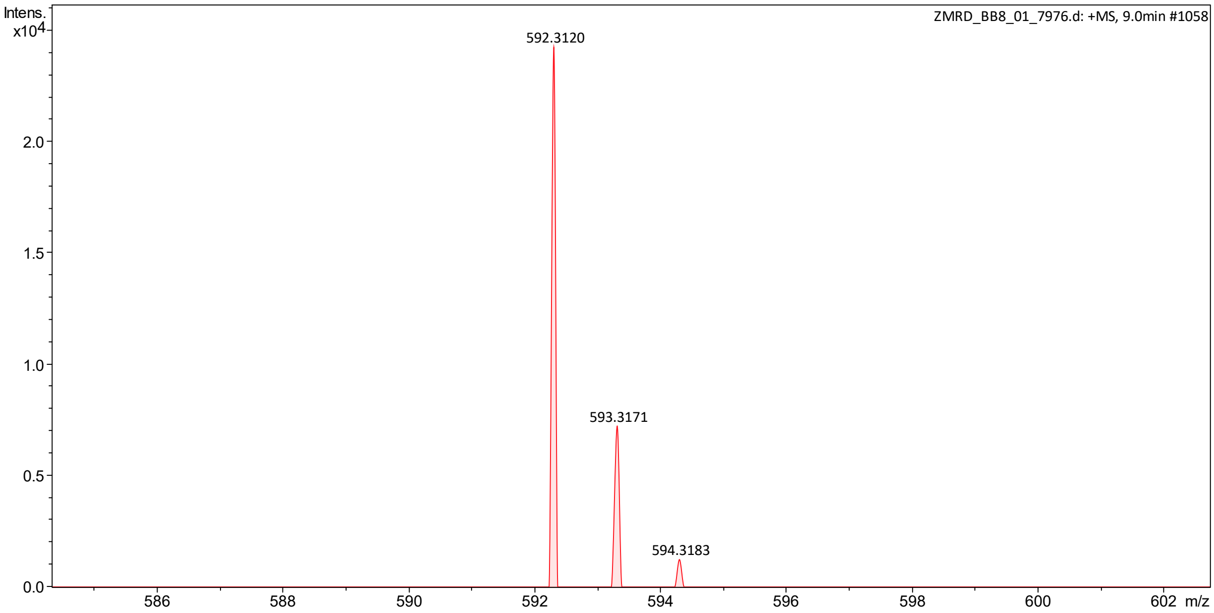

Figure S29: (+) ESI MS/MS spectrum of compound **15** (*m/z* 592)

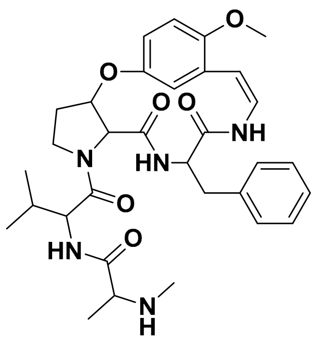

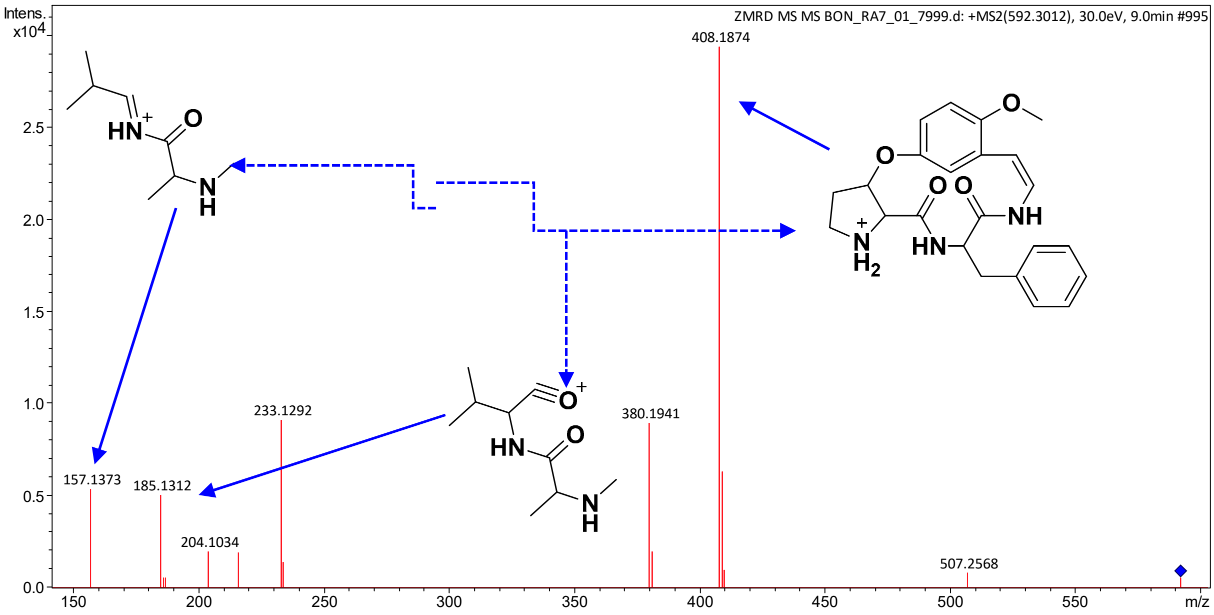

Figure S30: (+) ESI MS/MS spectrum of compound **15** (m/z 592)

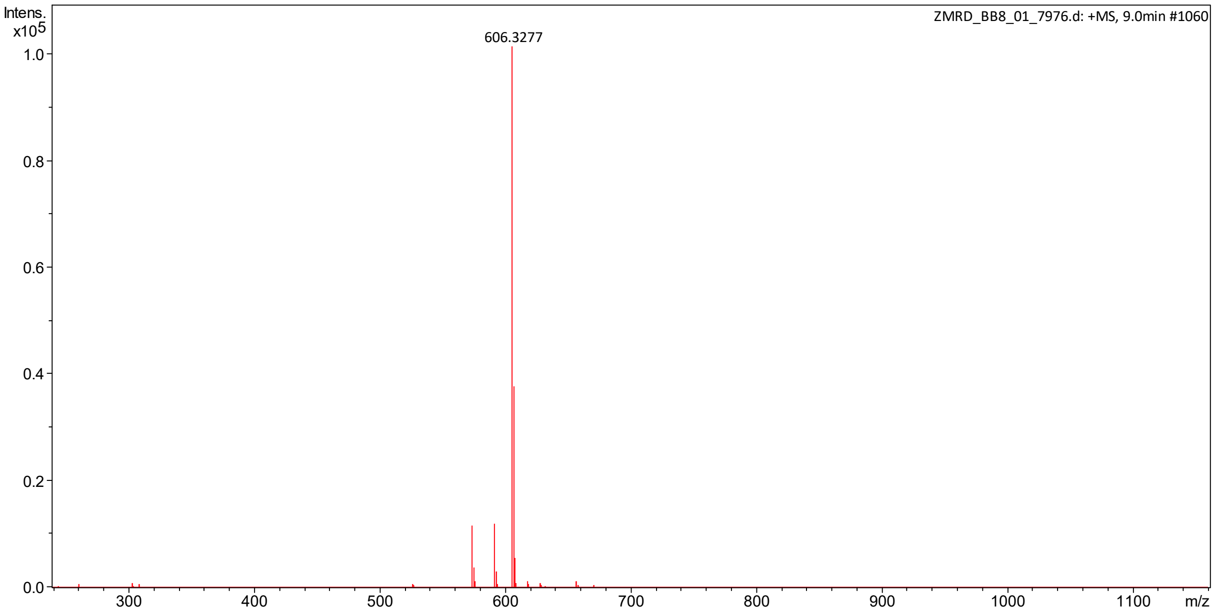

Figure S31: (+) HRESIMS of compound **16** (*m/z* 606)

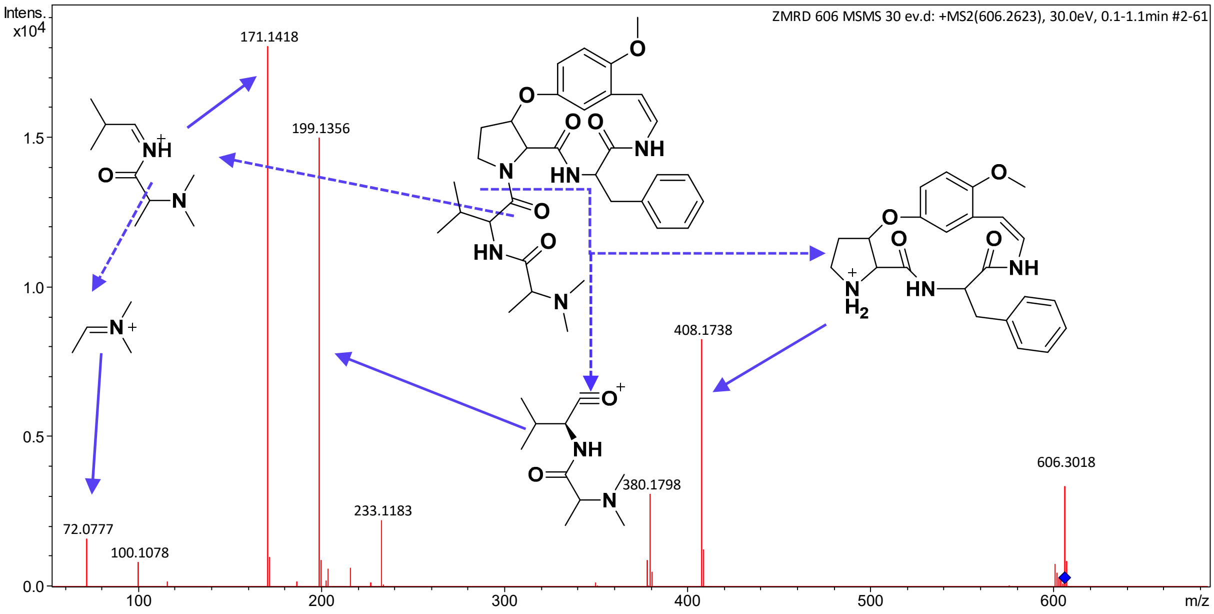

Figure S32: (+) HRESIMS/MS of compound **16** (*m/z* 606)

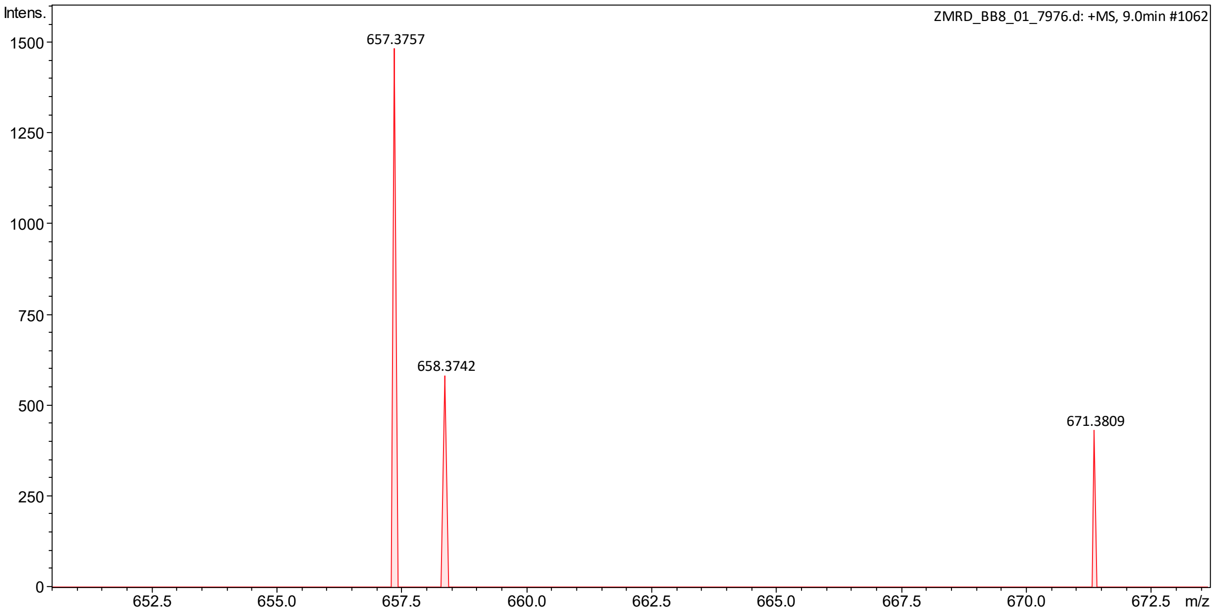

Figure S33: (+) HRESIMS spectrum of compound **17** (*m/z* 657)

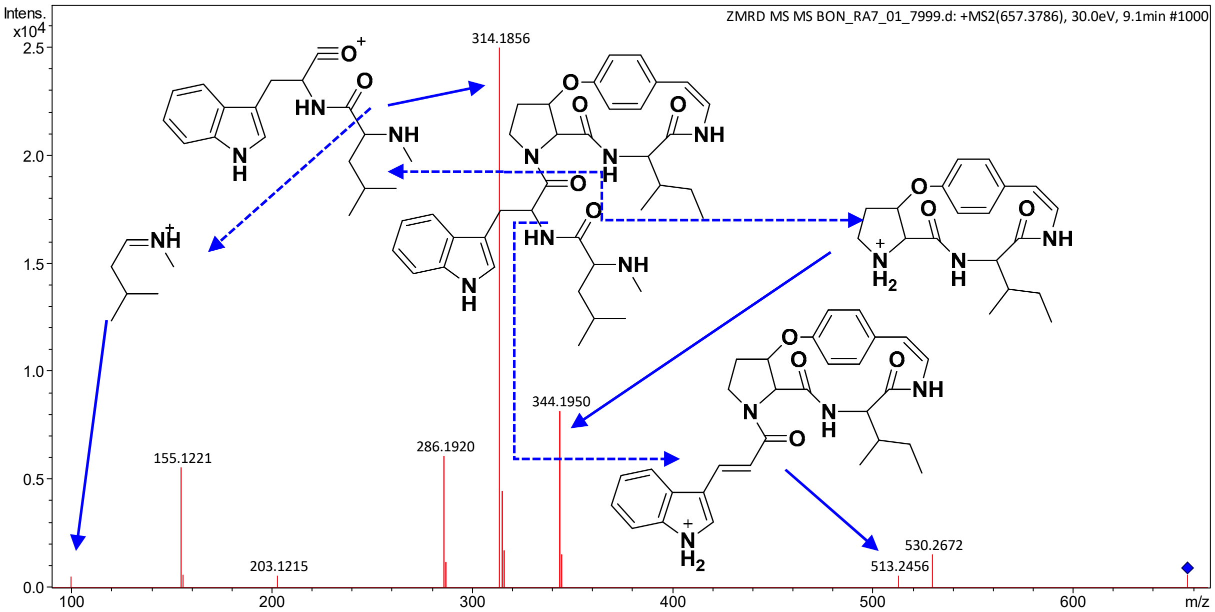

Figure S34: (+) ESI MS/MS spectrum of compound **17** (*m/z* 657)

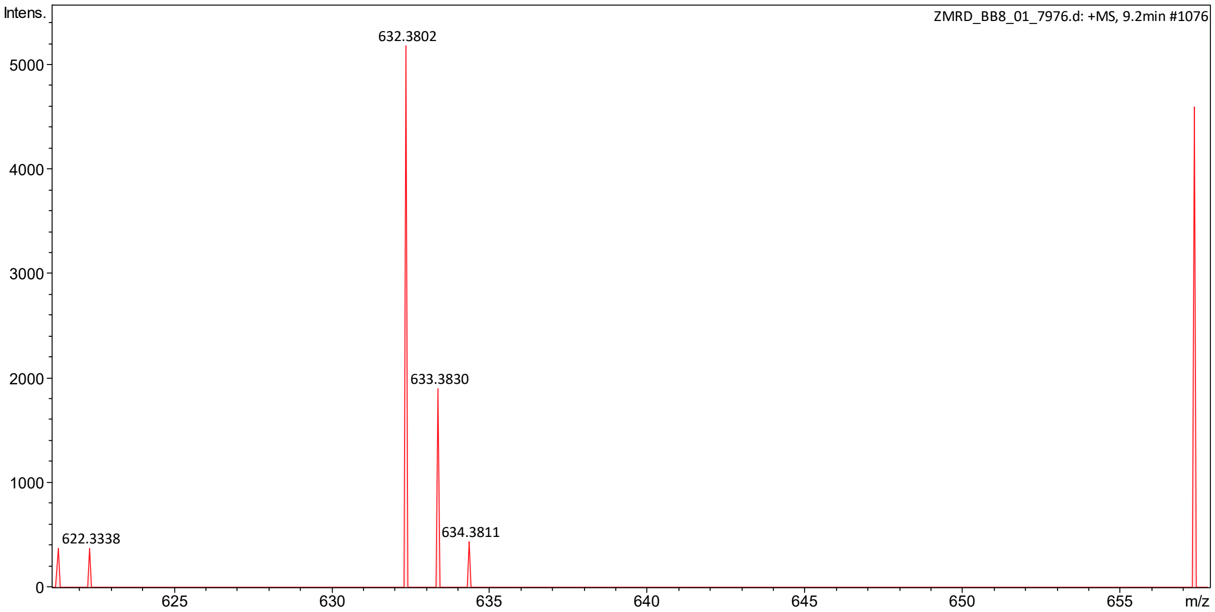

Figure S35: (+) HRESIMS spectrum of compound **18** (*m/z* 632)

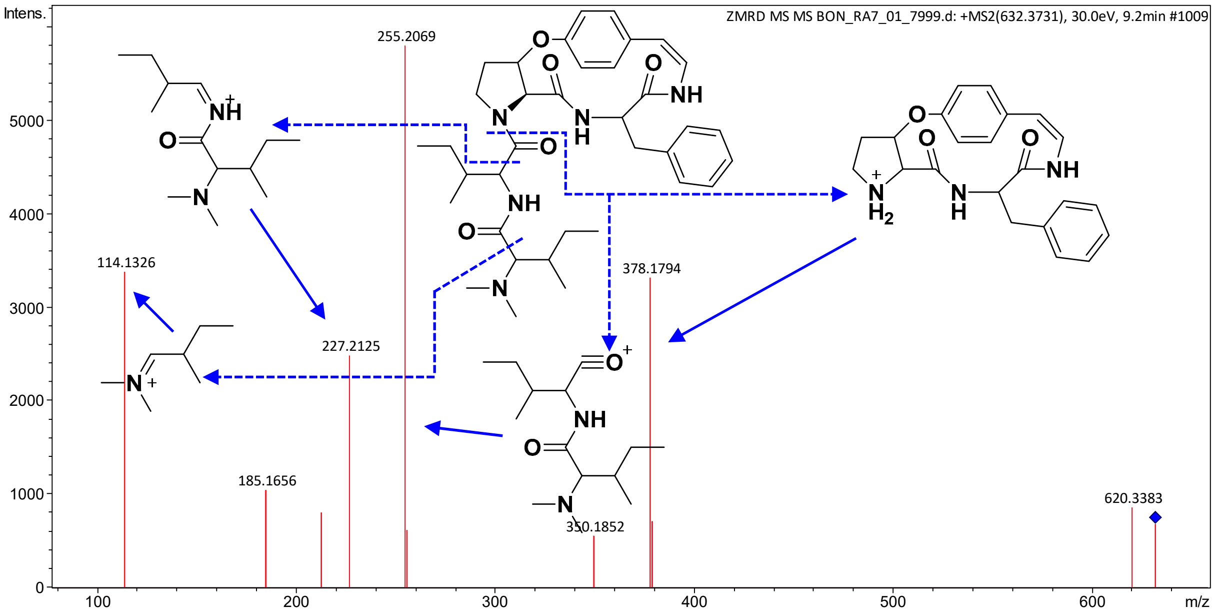

Figure S36: (+) ESI MS/MS spectrum of compound **18** (*m/z* 632)

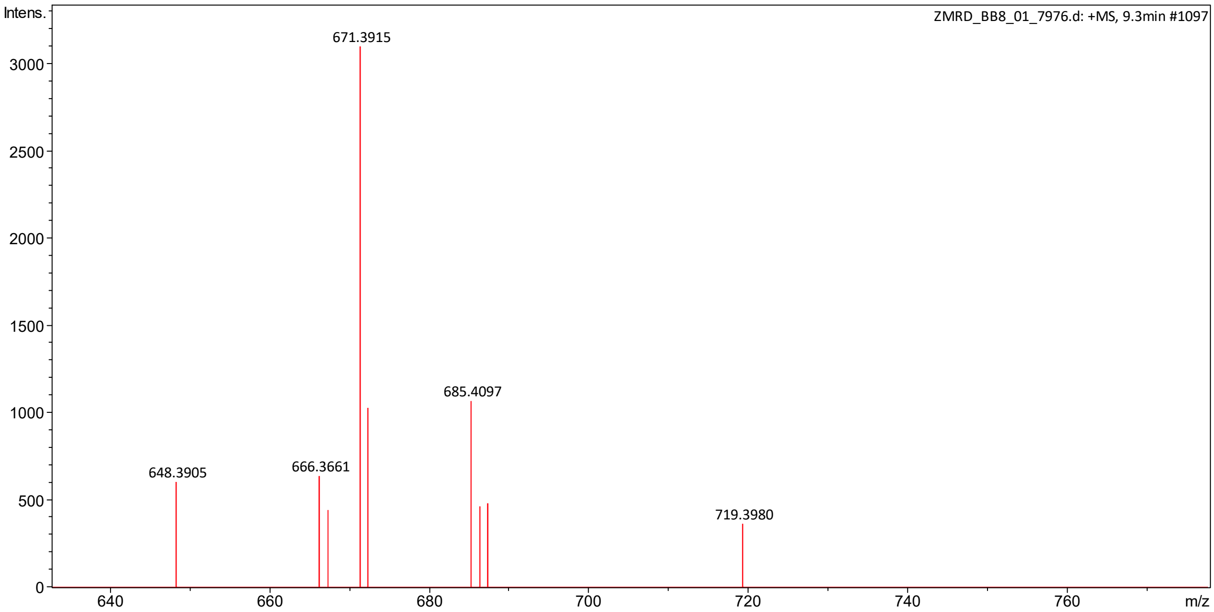

Figure S37: (+) HRESIMS of compound **19** (*m/z* 671)

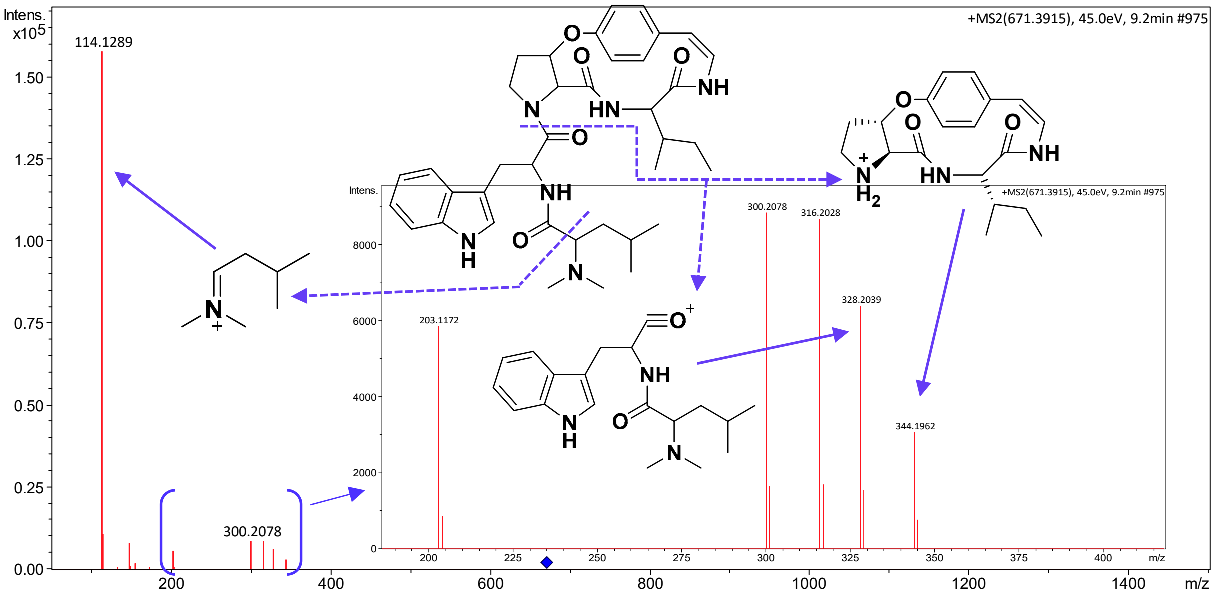

Figure S38: (+) HRESIMS/MS of compound **19** (*m/z* 671)

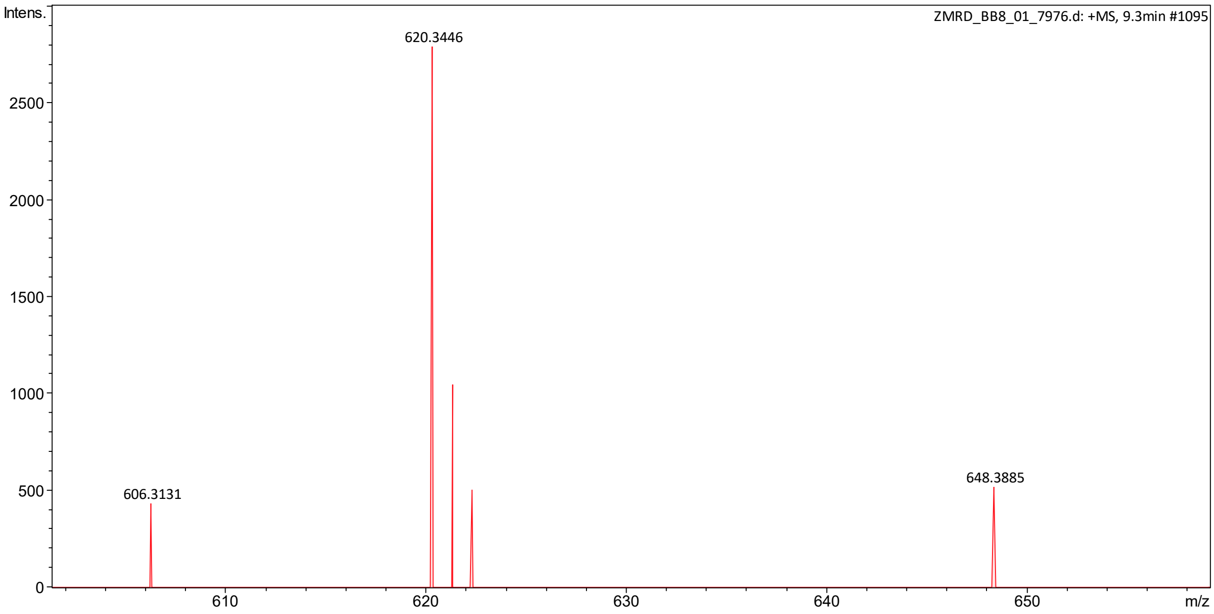

Figure S39: (+) HRESIMS spectrum of compound **20** (*m/z* 620)

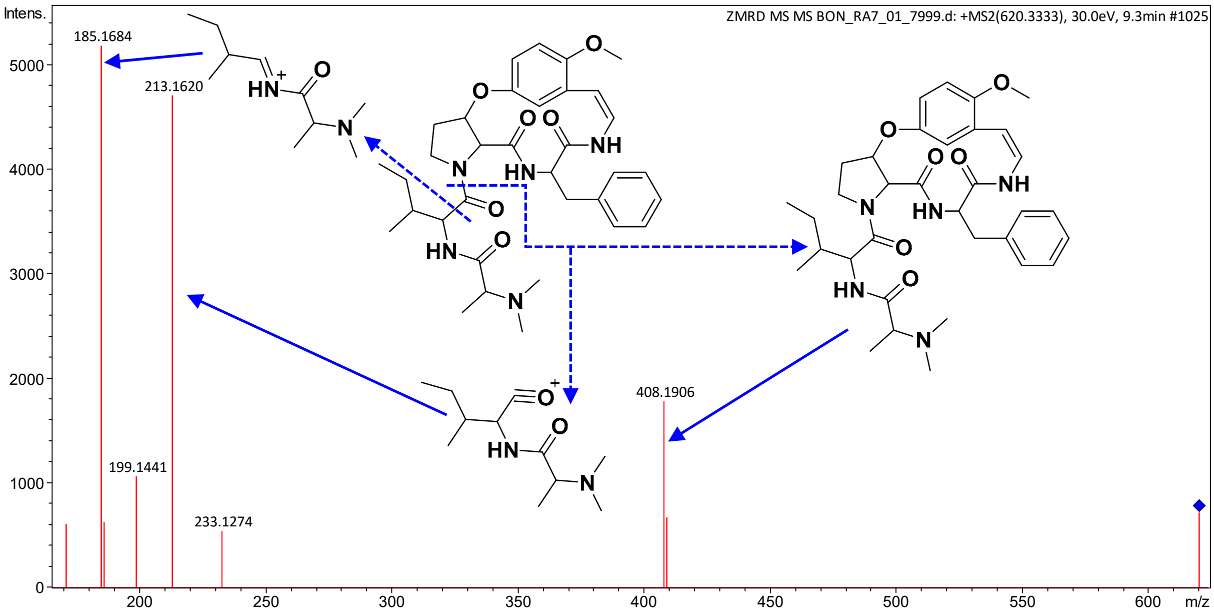

Figure S40: (+) ESI MS/MS spectrum of compound **20** (*m/z* 620)

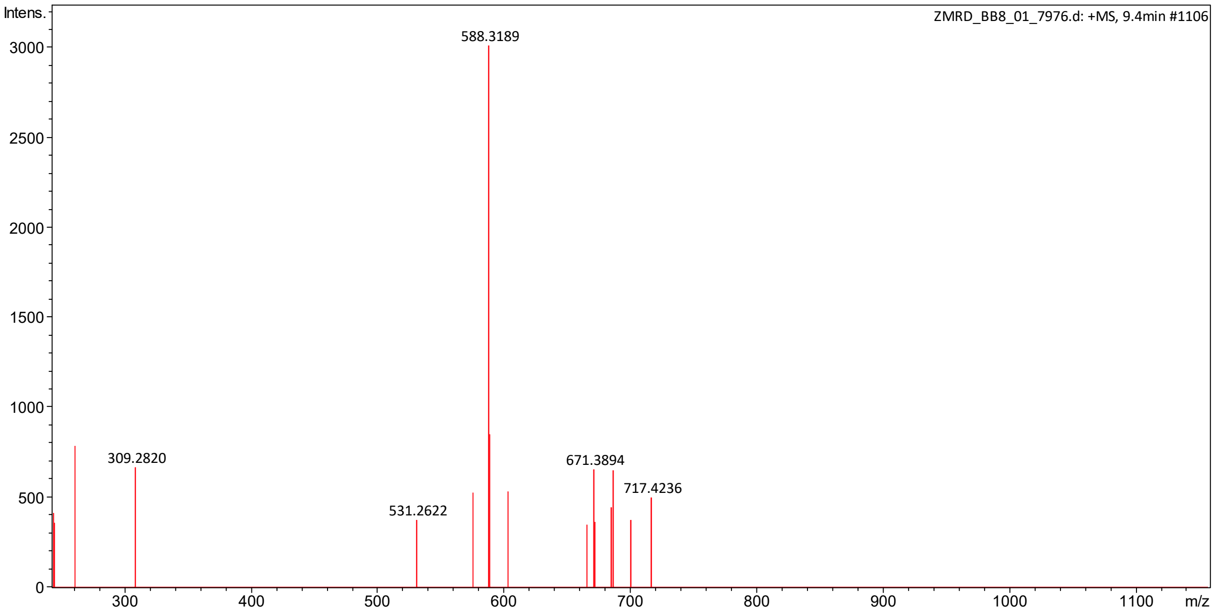

Figure S41: (+) HRESIMS of compound **21** (*m/z* 588)

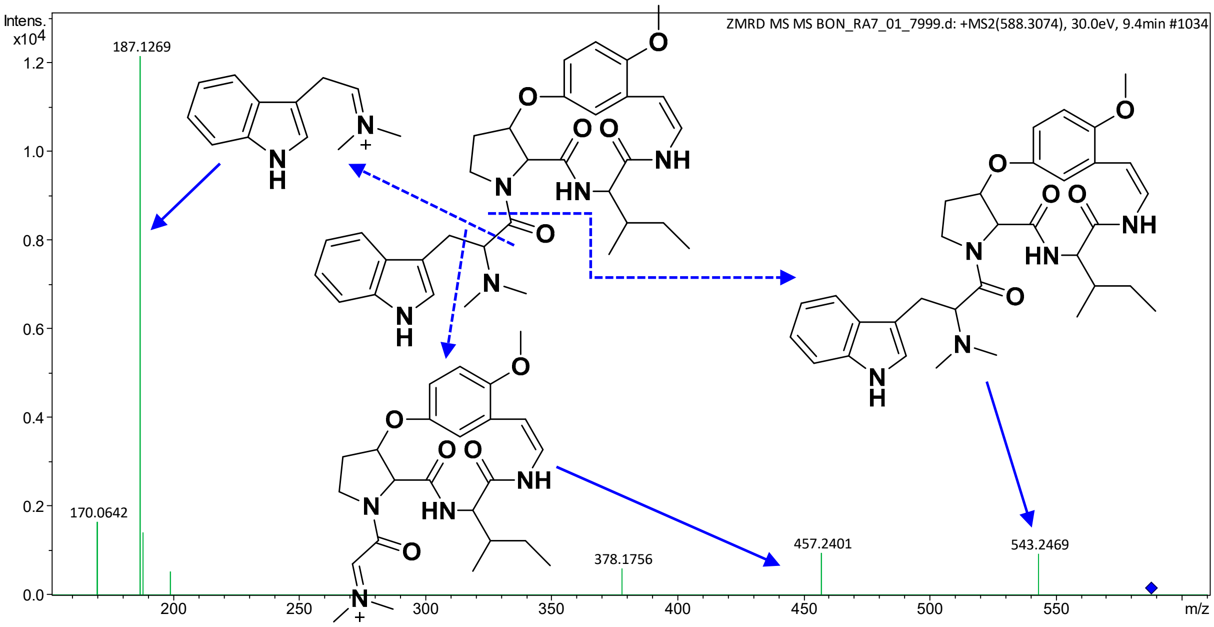

Figure S42: (+) HRESIMS/MS of compound **21** (*m/z* 588)

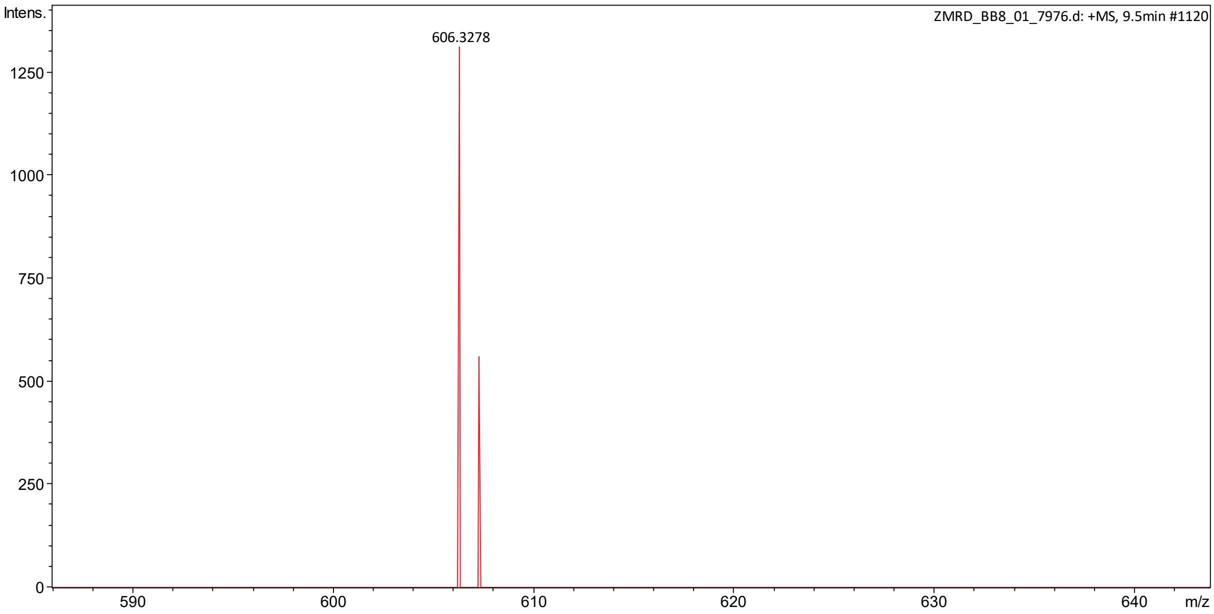

Figure S43: (+) HRESIMS/MS of compound **22** (*m/z* 606)

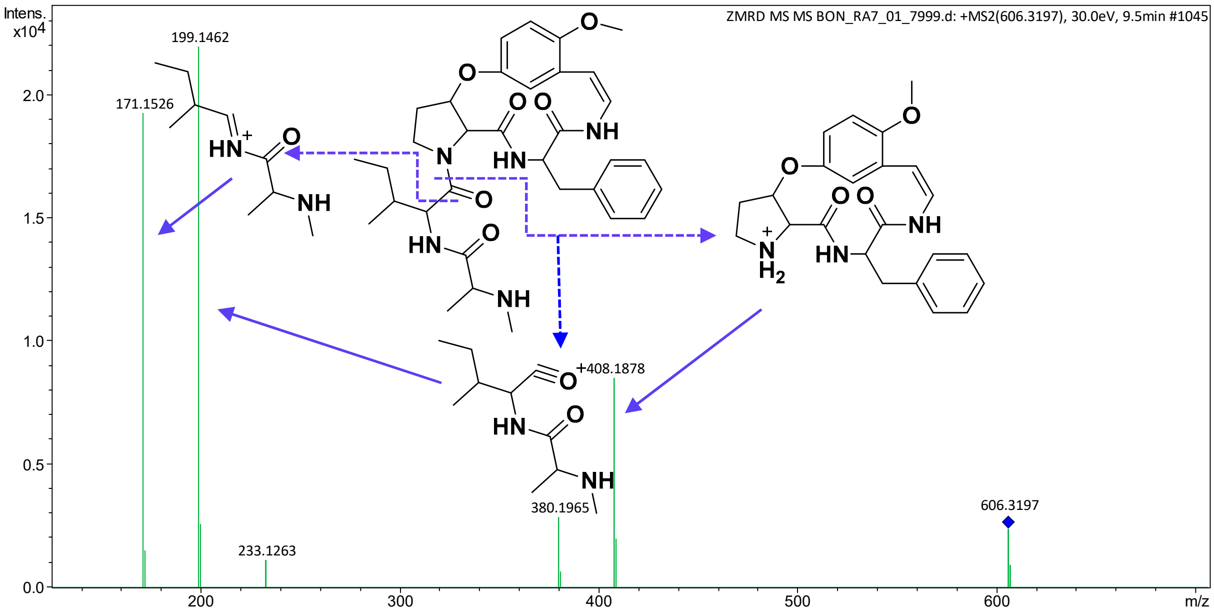

Figure S44: (+) HRESIMS/MS of compound **22** (*m*/*z* 606)

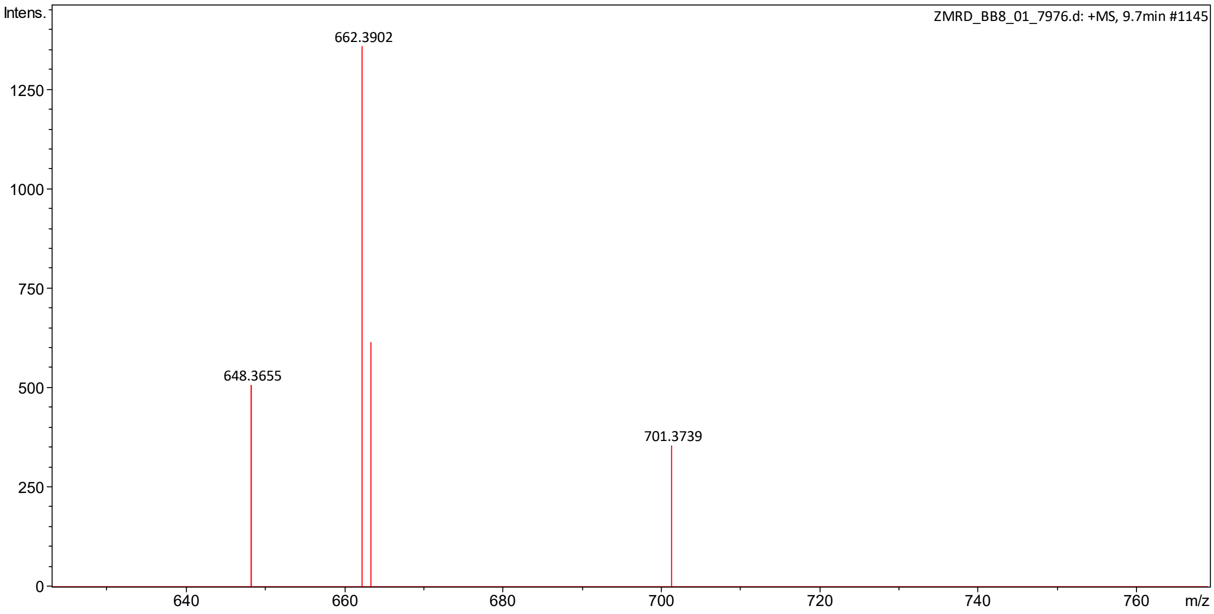

Figure S45: (+) HRESIMS spectrum of compound **23** (*m*/*z* 662)

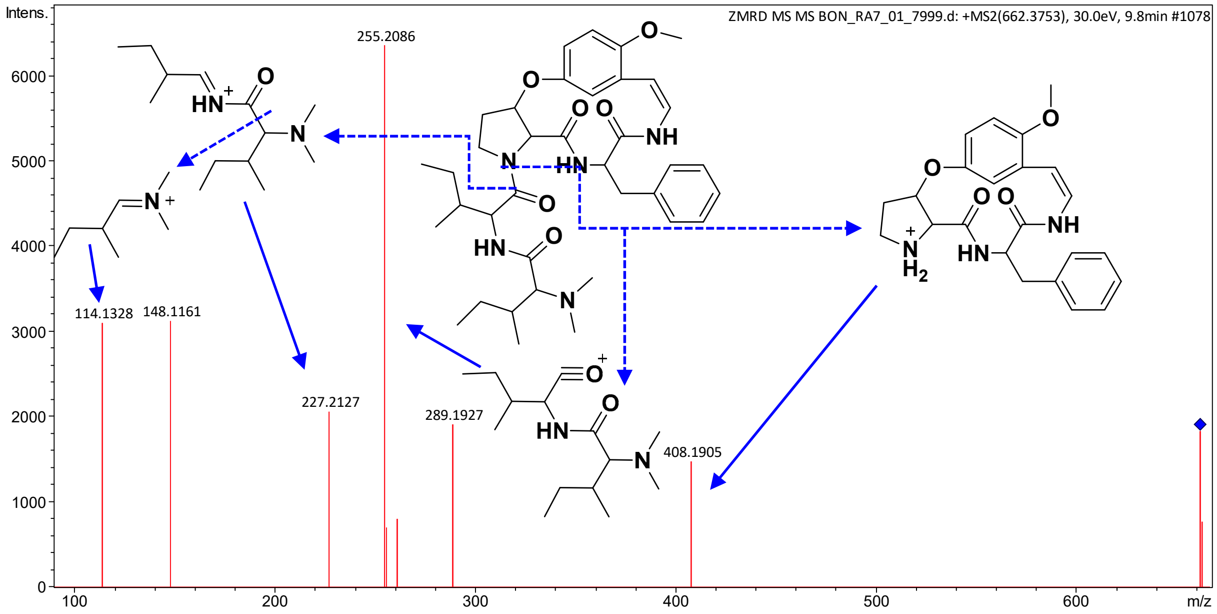

Figure S46: (+) ESI MS/MS spectrum of compound **23** (*m*/*z* 662)

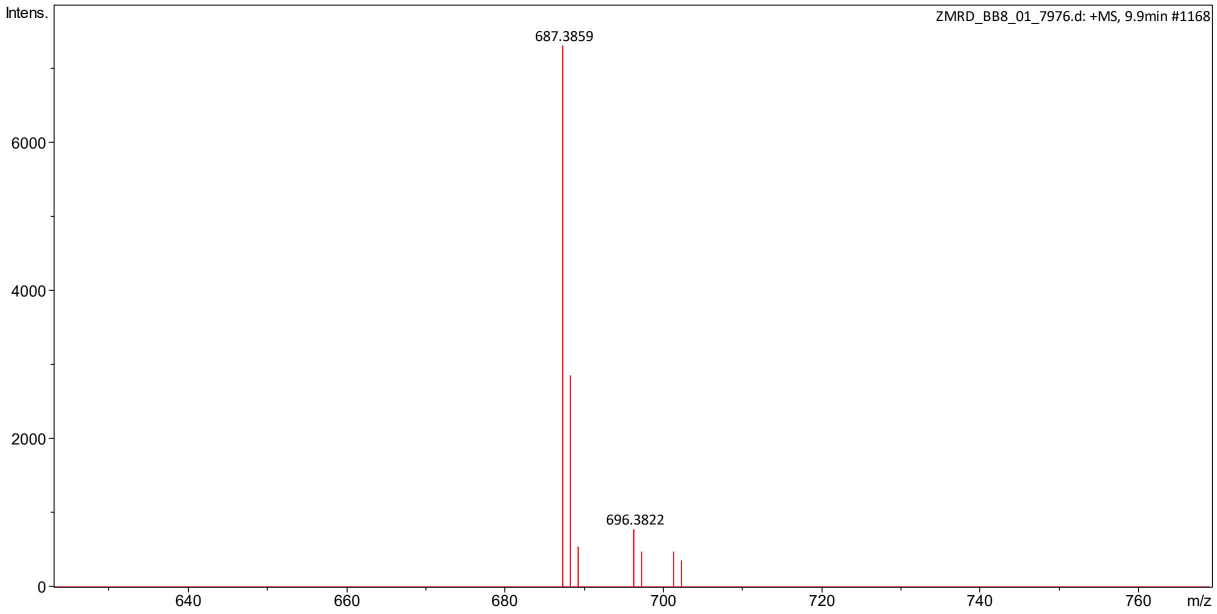

Figure S47: (+) HRESIMS of compound **24** (*m*/*z* 687)

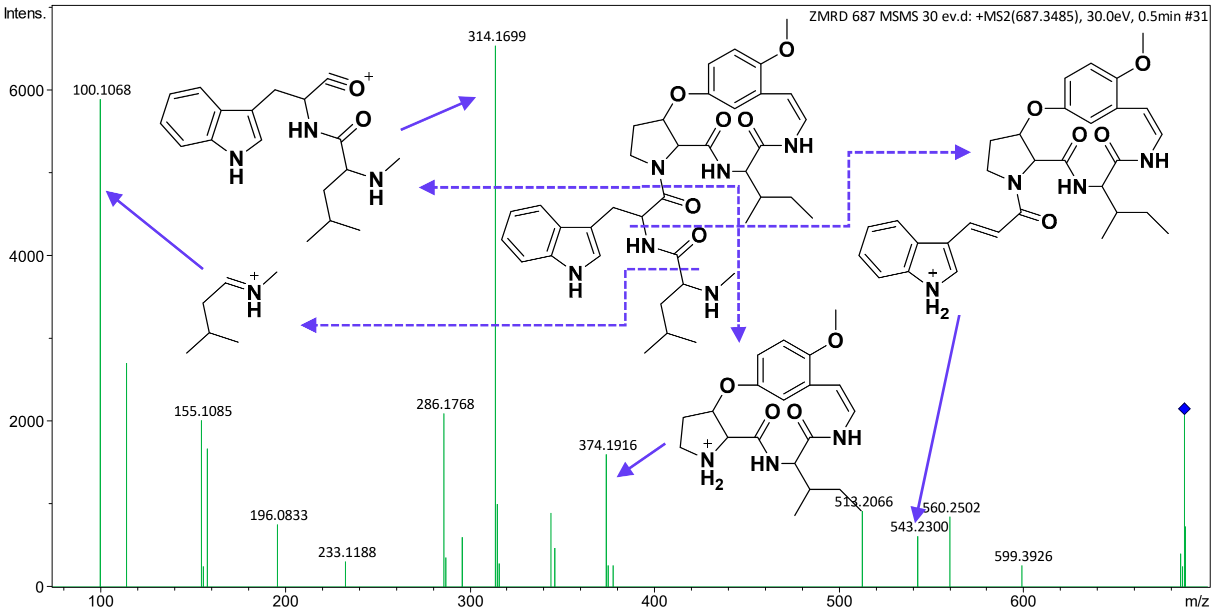

Figure S48: (+) HRESIMS/MS of compound **24** (*m/z* 687)

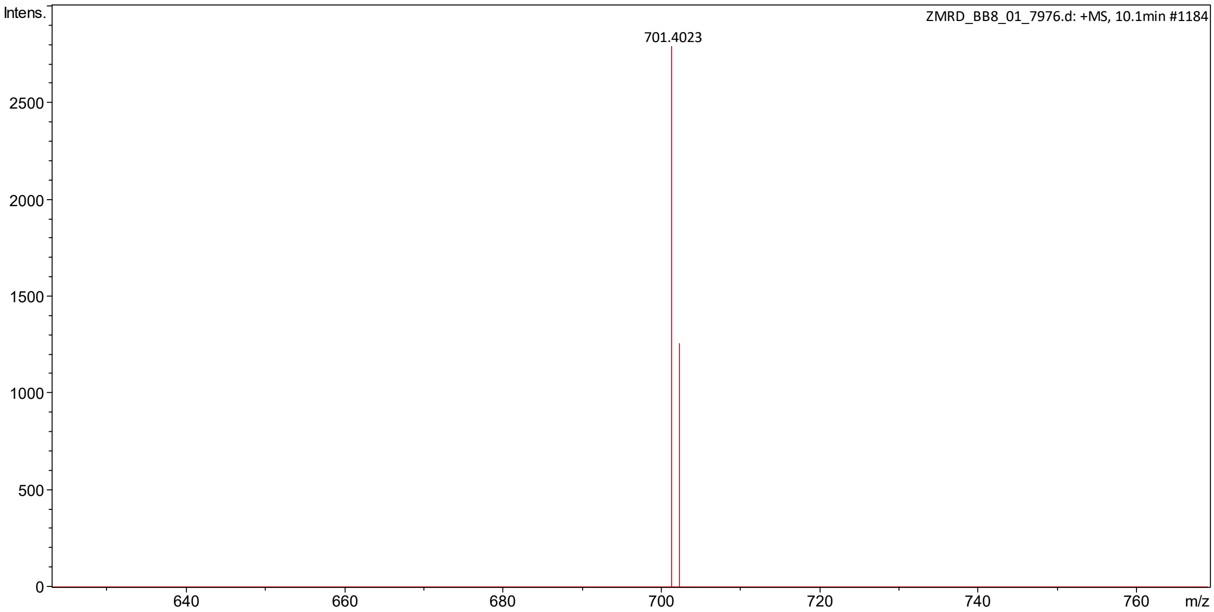

Figure S49: (+) HRESIMS spectrum of compound **25** (*m/z* 701)

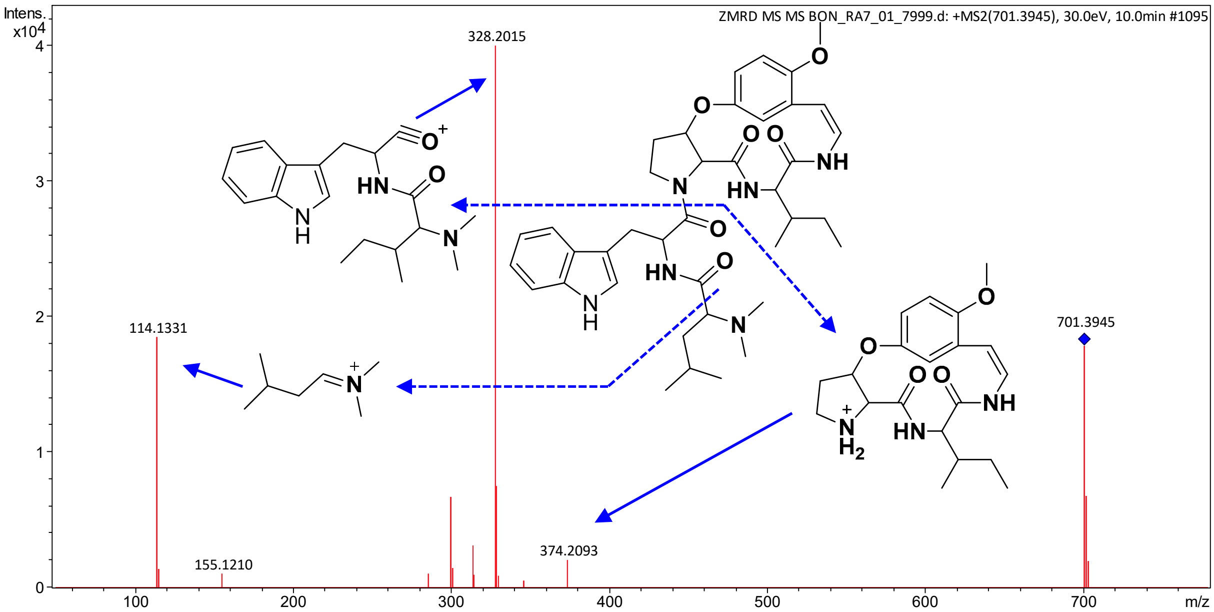

Figure S50: (+) ESI MS/MS spectrum of compound **25** (*m/z* 701)

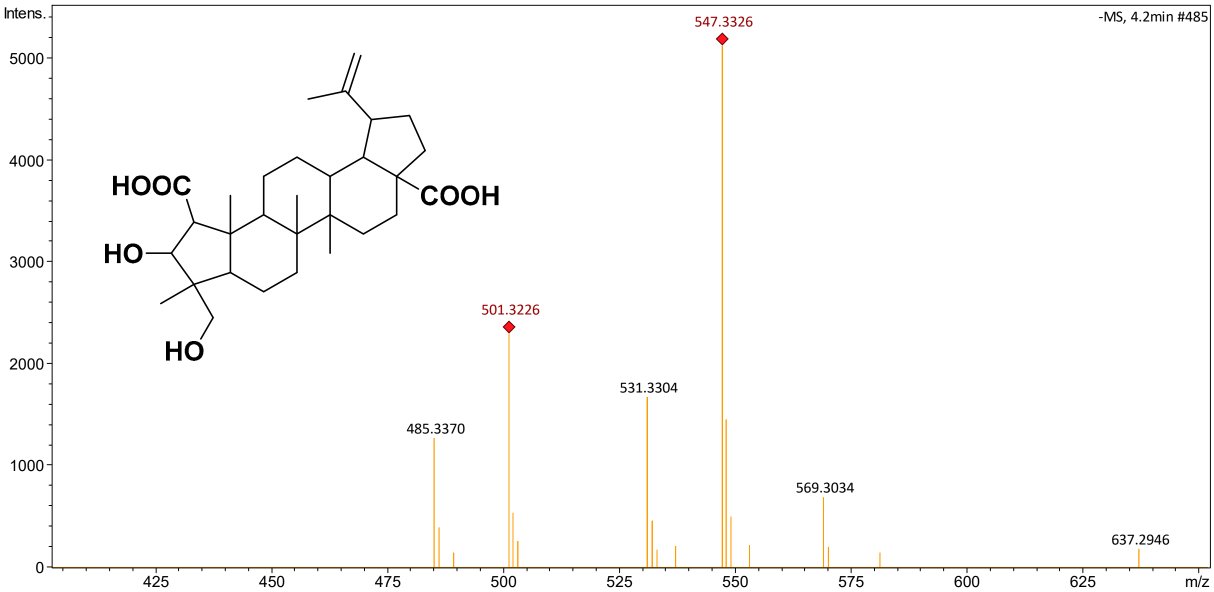

Figure S51: (+) HRESIMS spectrum of compound **26** (*m/z* 501)

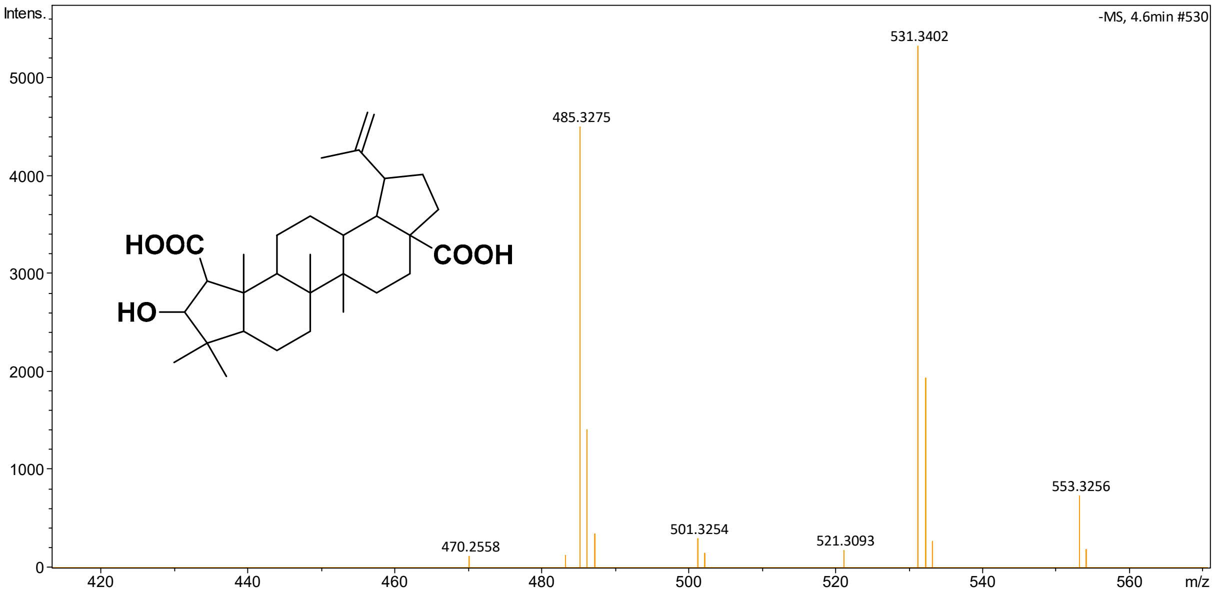

Figure S52: (+) HRESIMS spectrum of compound **27** (*m/z* 485)

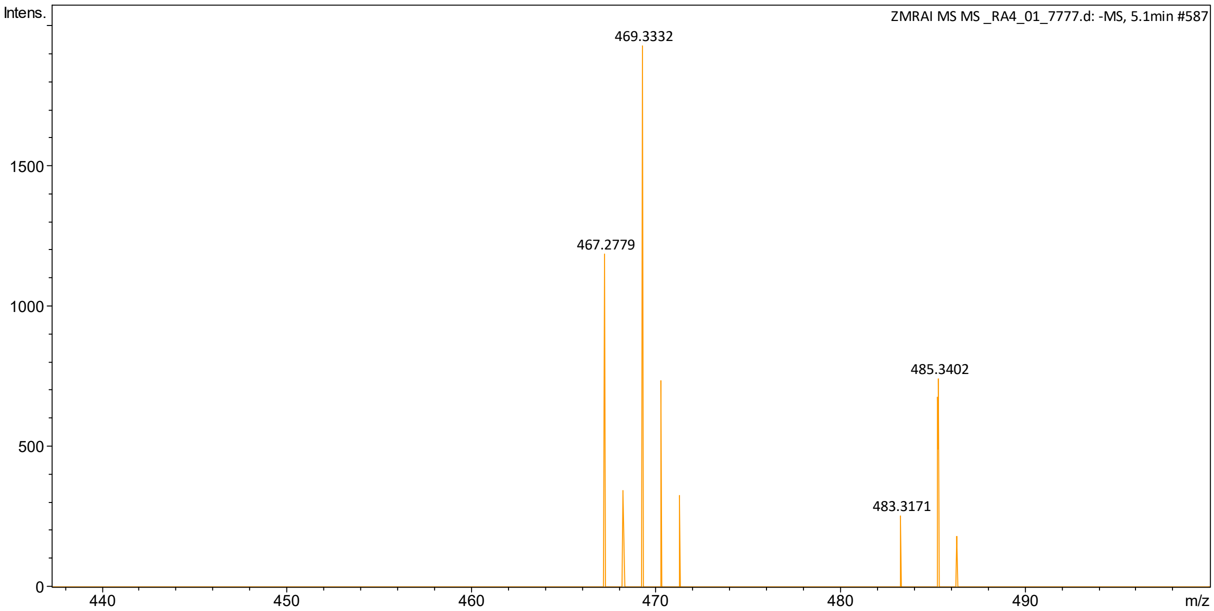

Figure S53: (+) HRESIMS spectrum of compound **28** (*m/z* 469)

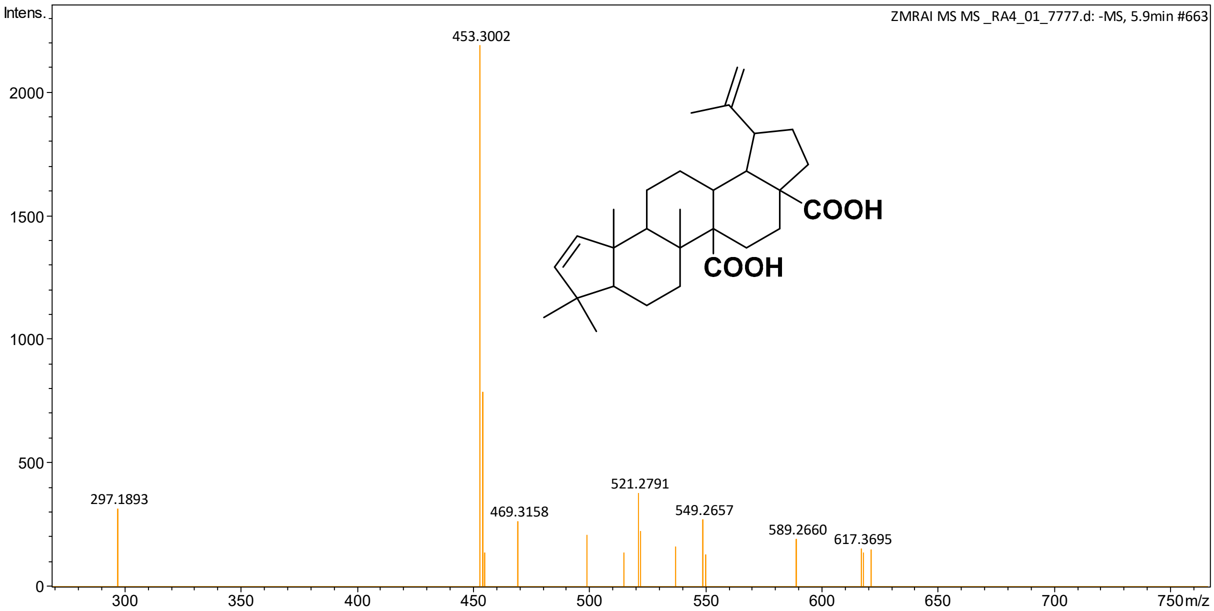

Figure S54: (+) HRESIMS spectrum of compound **29** (*m/z* 453)

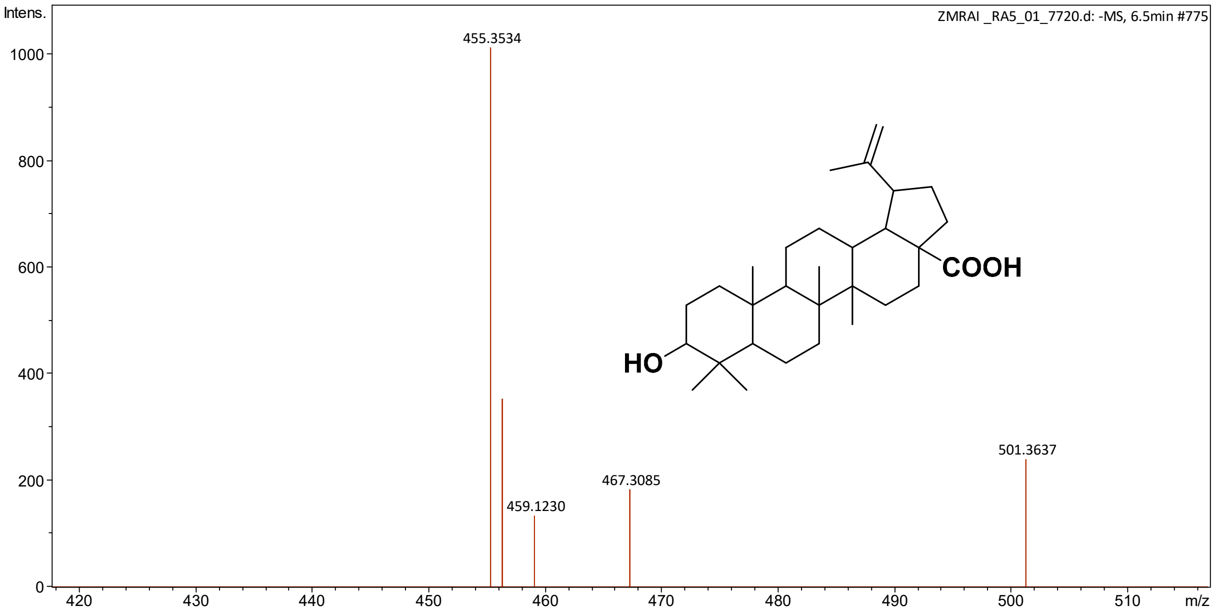

Figure S55: (+) HRESIMS spectrum of compound **30** (*m/z* 455)

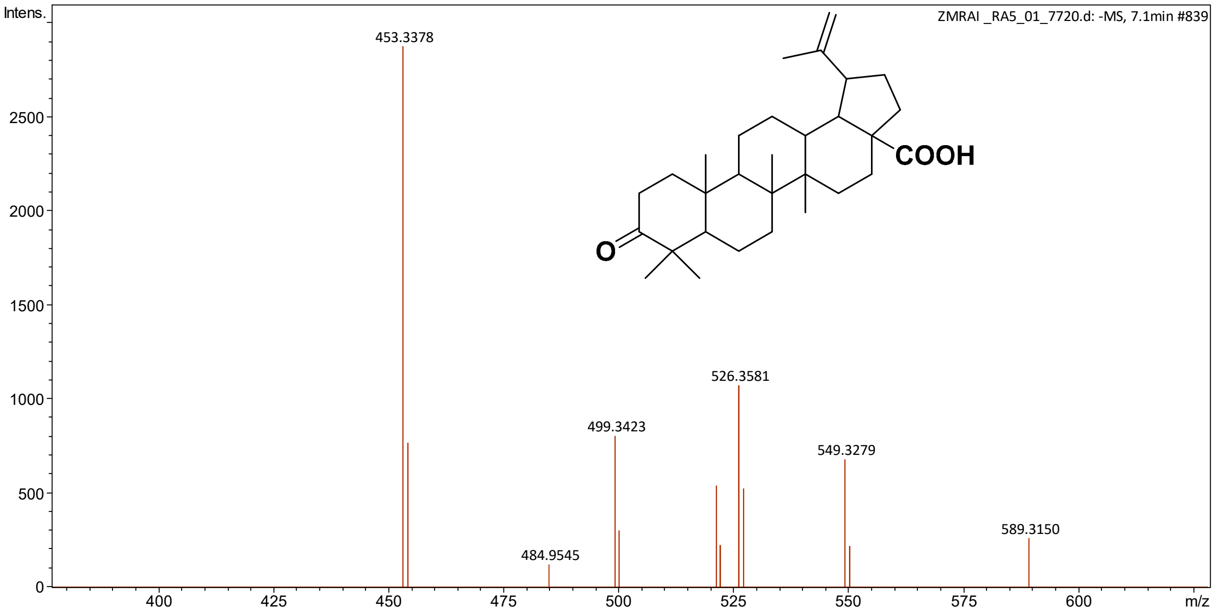

Figure S56: (+) HRESIMS spectrum of compound **31** (*m/z* 453)

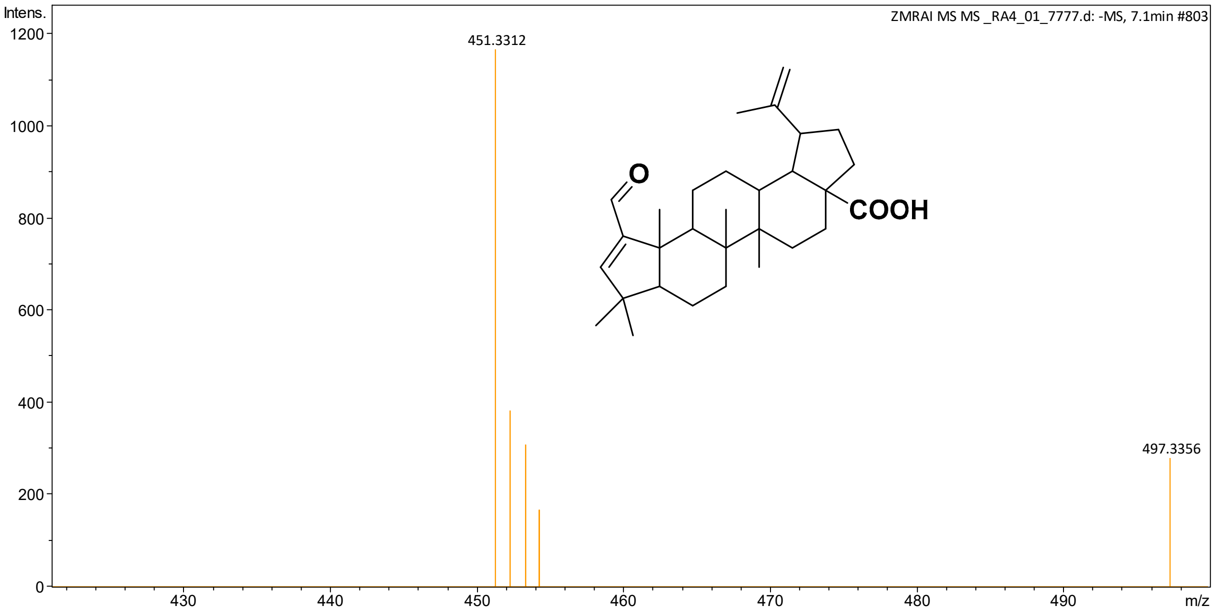

Figure S57: (+) HRESIMS spectrum of compound **32** (*m/z* 451)

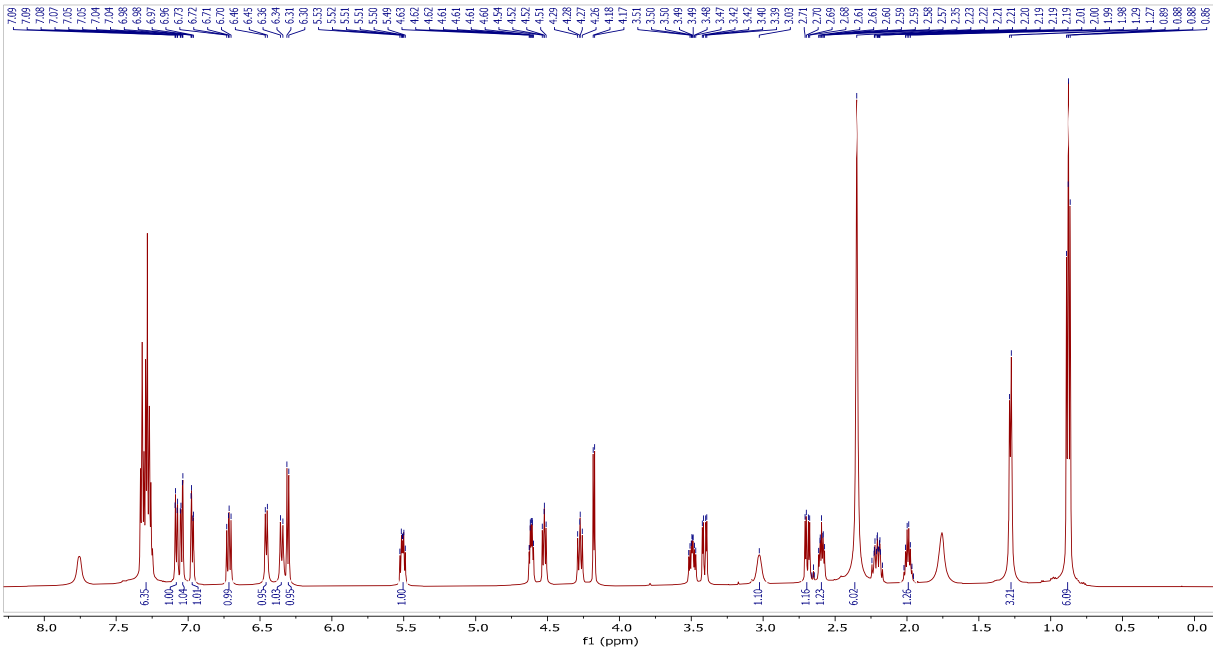

Figure S58: ^{1}HNMR spectrum ($CDCl_3$, 600 MHz) of compound **5**

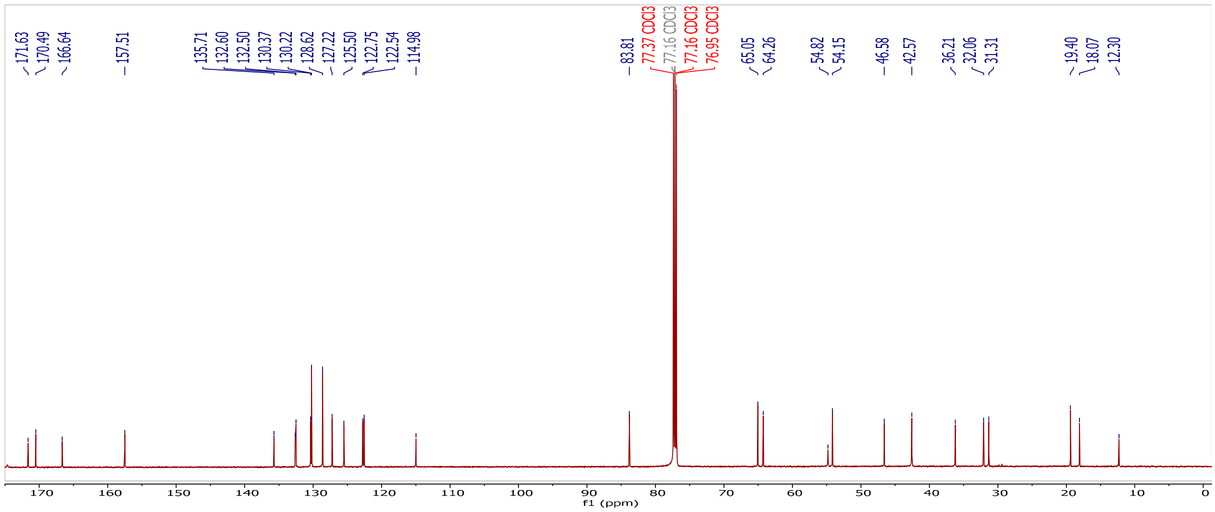

Figure S59: ^{13}CNMR spectrum ($CDCl_3$, 150 MHz) of compound **5**

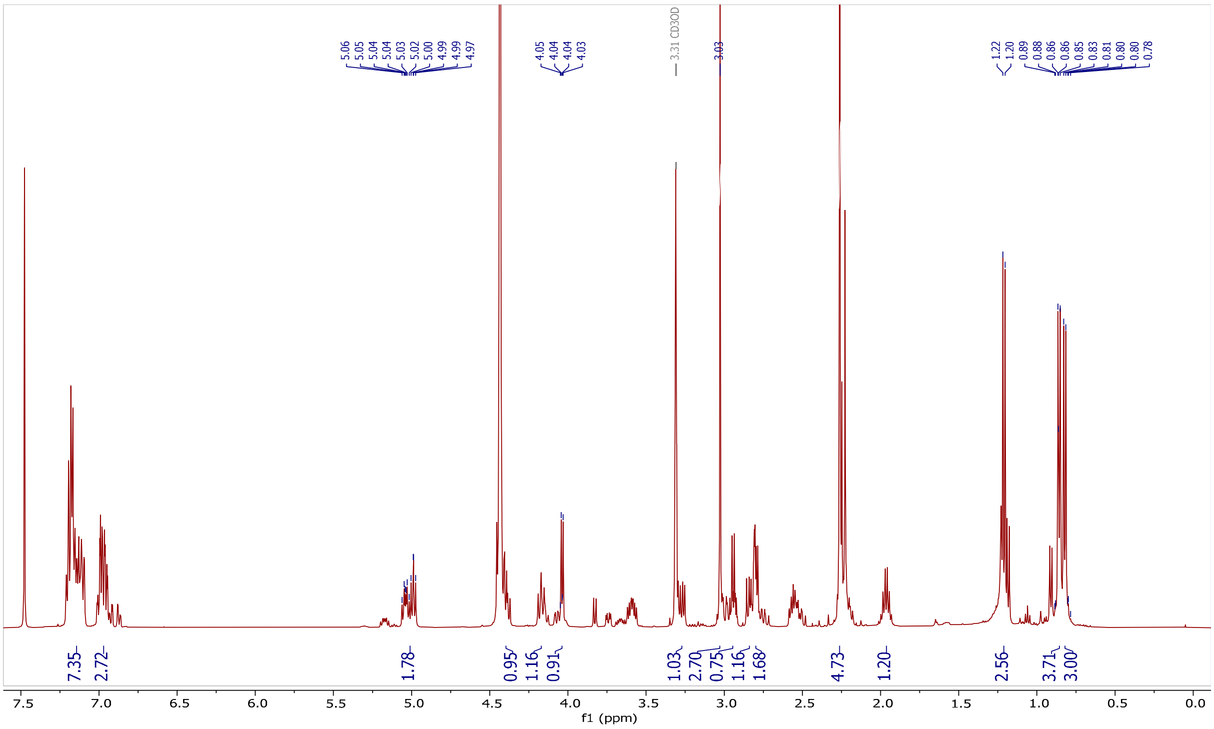

Figure S60: ^{1}HNMR spectrum ($CDCl_3$+ CD_3OD, 600 MHz) of compound **6**

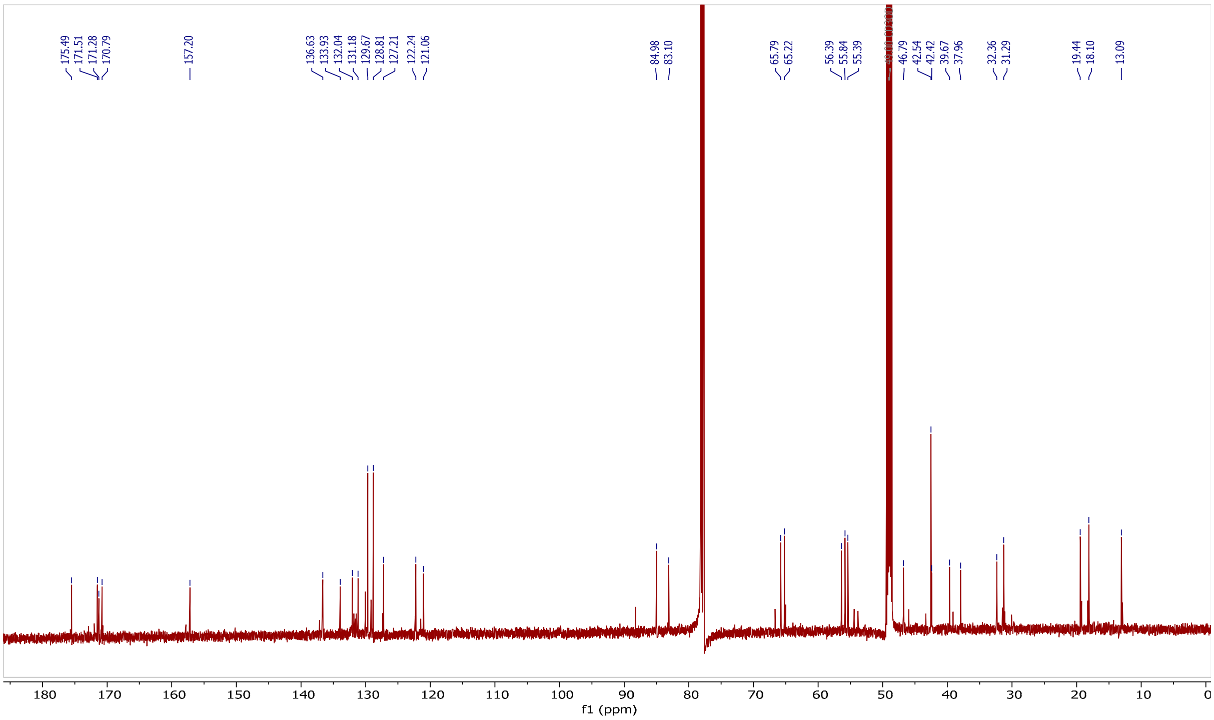

Figure S61: ^{13}CNMR spectrum ($CDCl_3$ + CD_3OD, 150 MHz) of compound **6**

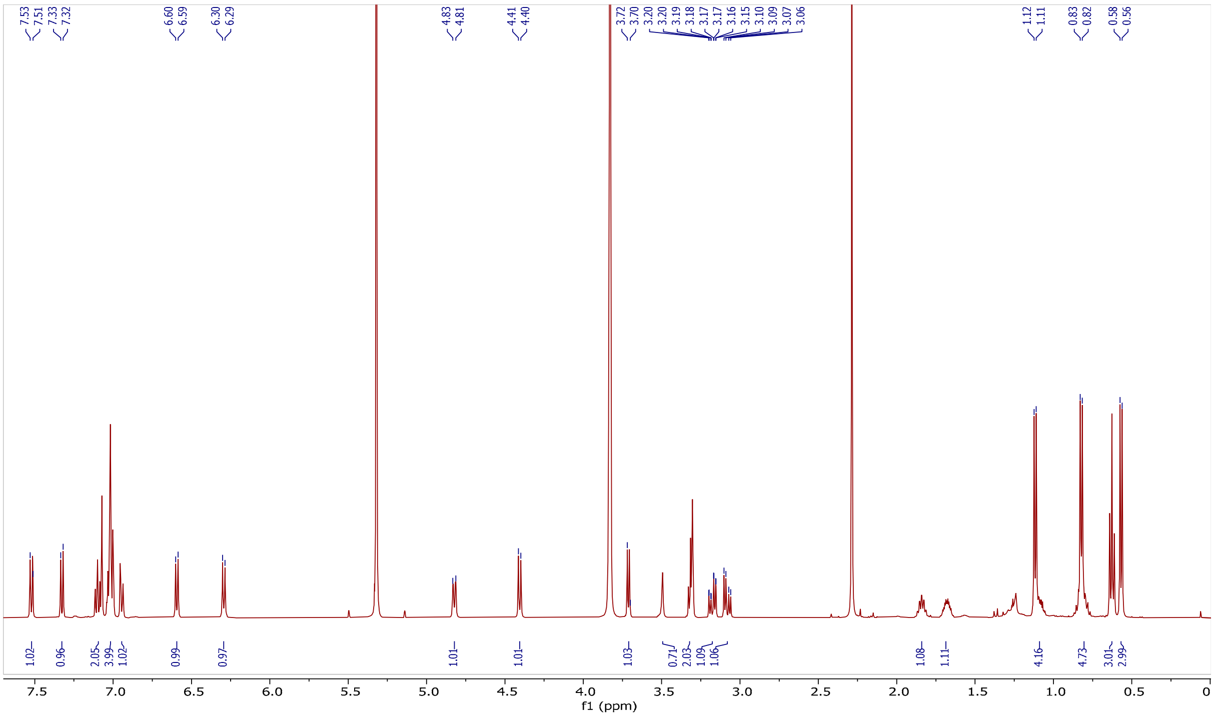

Figure S62: ^{1}HNMR spectrum ($CDCl_3$+ CD_3OD, 600 MHz) of compound **14**

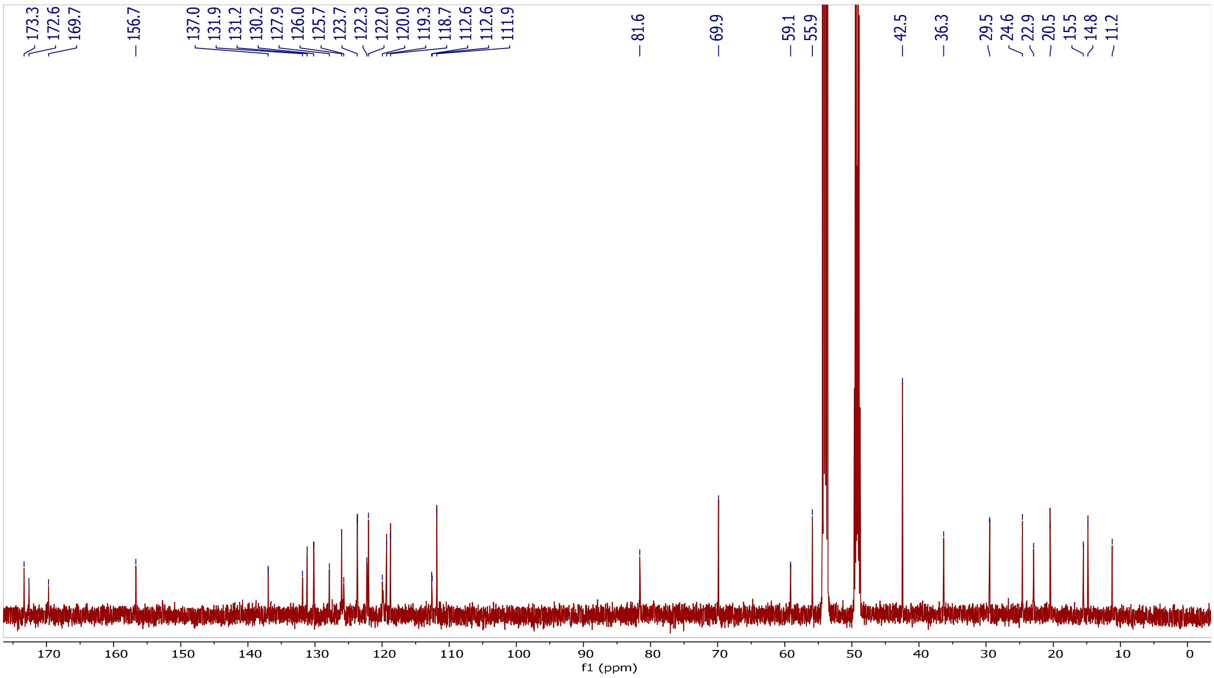

Figure S63: ^{13}CNMR spectrum ($CDCl_3$+ CD_3OD, 150 MHz) of compound **14**

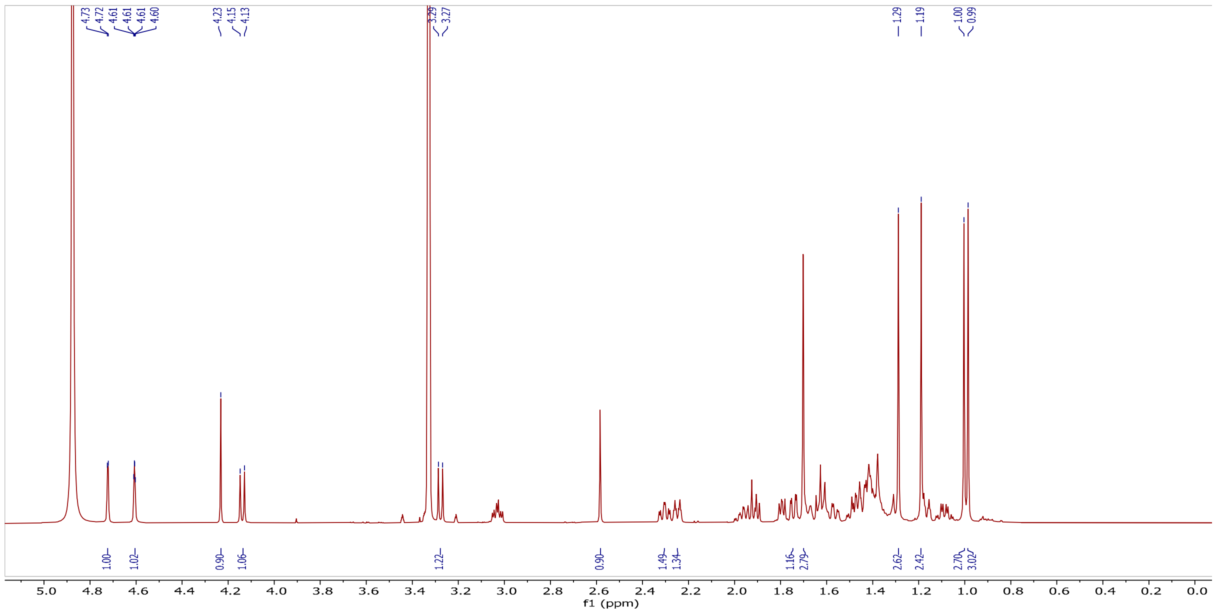

Figure S64: ^{1}H NMR spectrum (CD_3OD, 600 MHz) of compound **26**

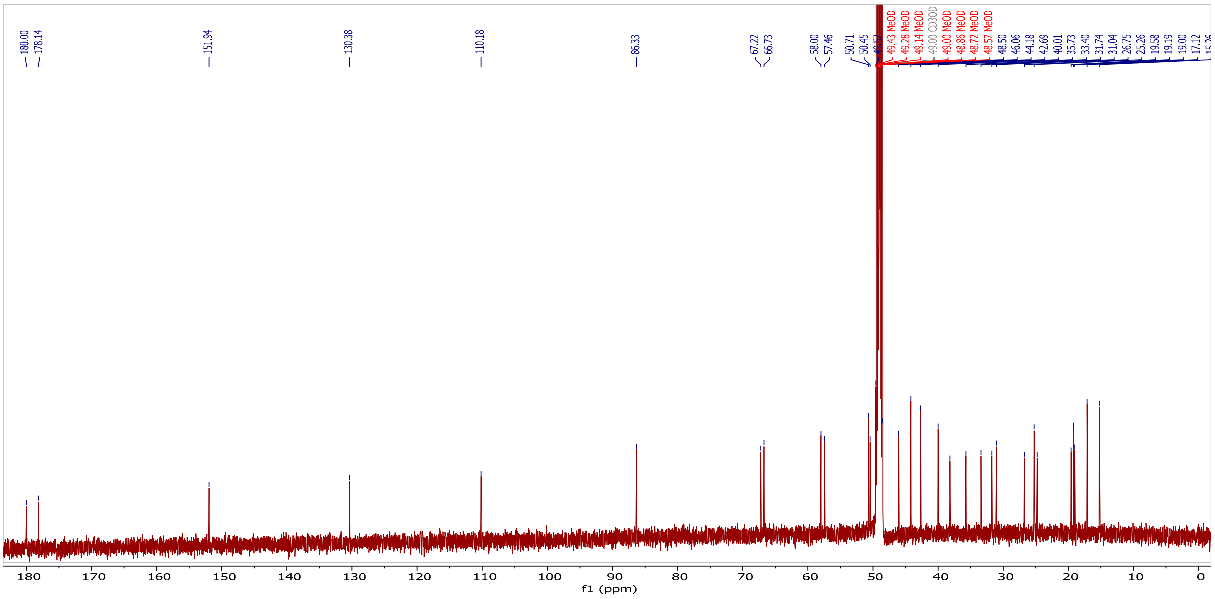

Figure S65: ^{13}CNMR spectrum (CD_3OD, 150 MHz) of compound **26**

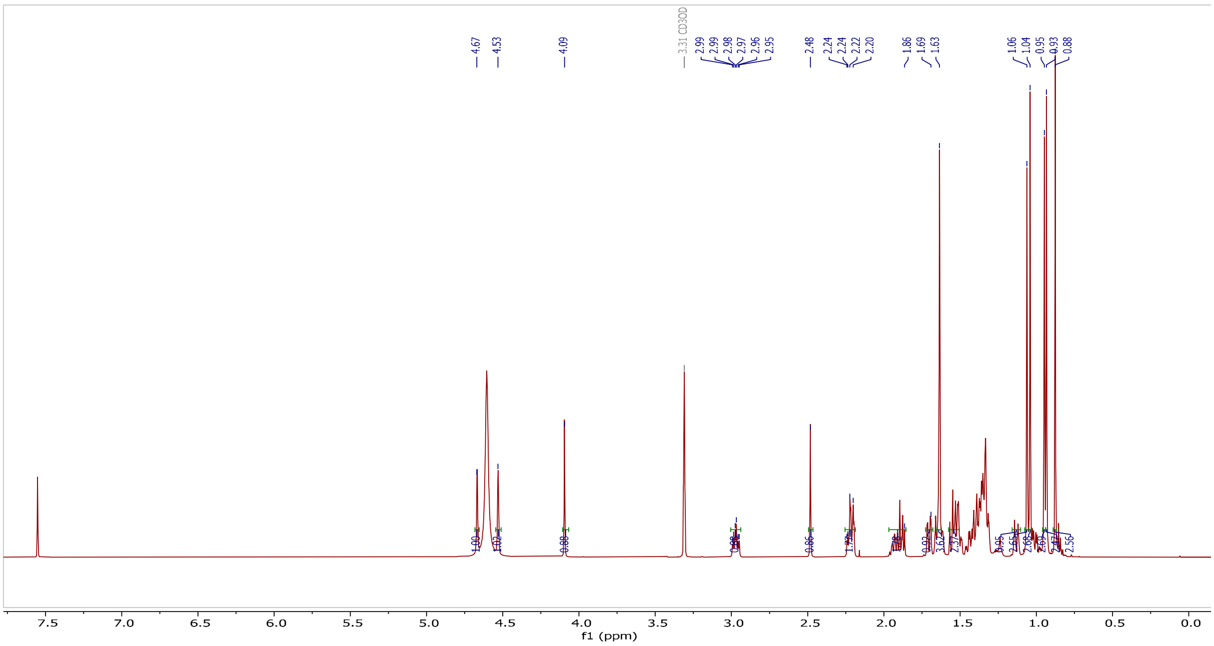

Figure S66: ^{1}H NMR spectrum ($CDCl_3$ / CD_3OD, 600MHz) of compound **27**

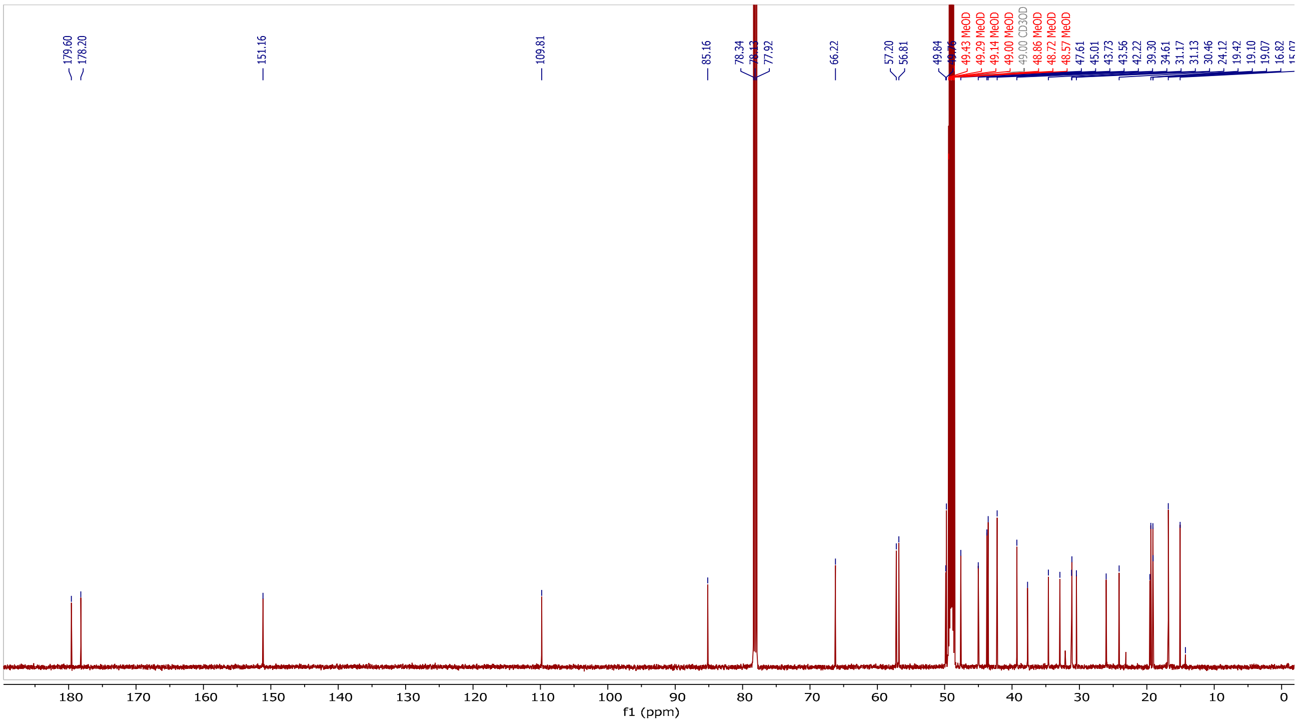

Figure S67: ^{13}C NMR spectrum ($CDCl_3$ / CD_3OD, 150 MHz) of compound **27**

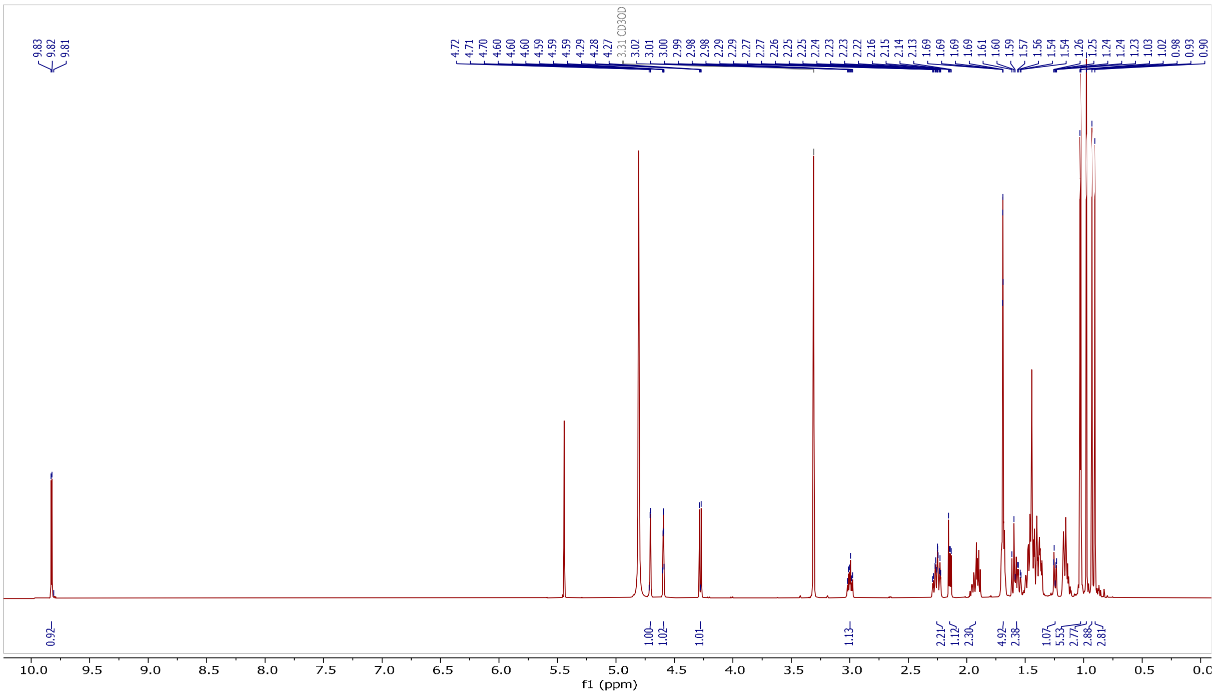

Figure S68: ^{1}HNMR spectrum (CD_3OD, 600 MHz) of compound **28**

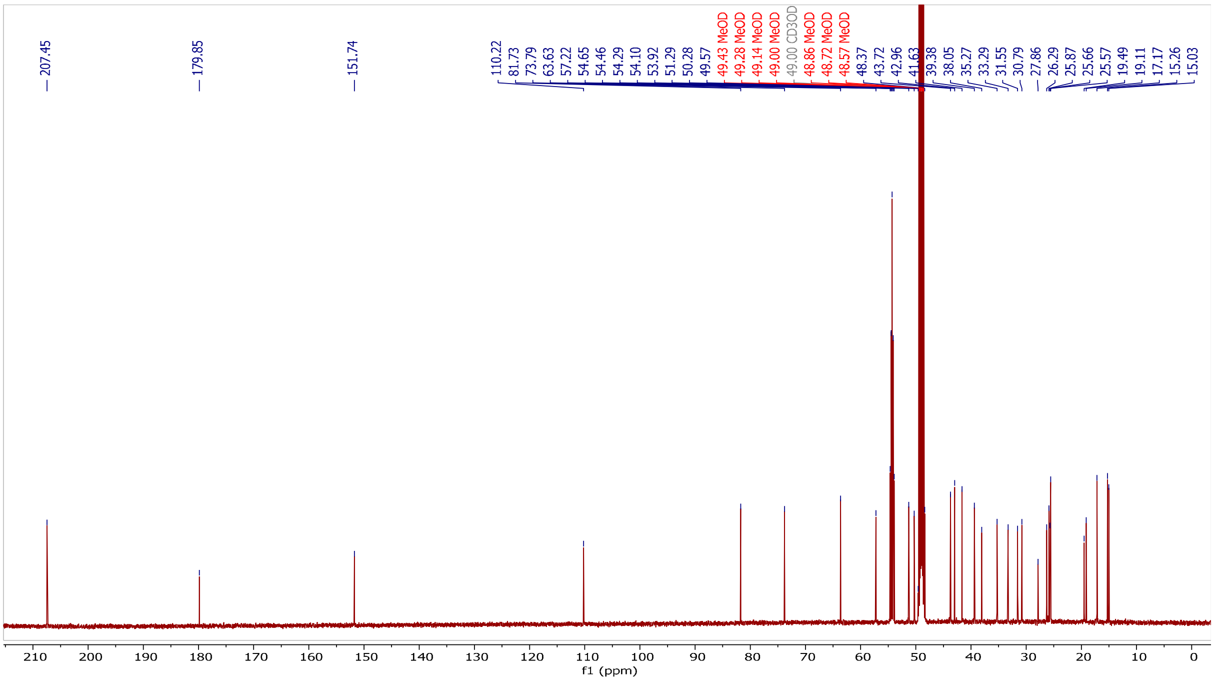

Figure S69: ^{13}CNMR spectrum (CD_3OD, 600 MHz) of compound **28**

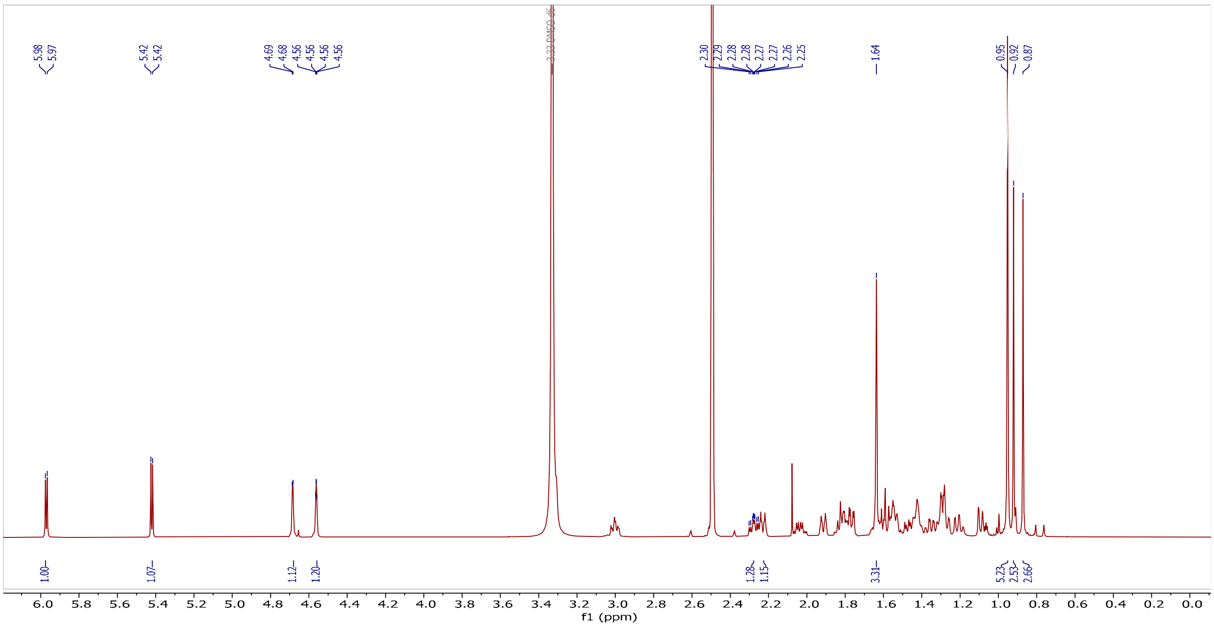

Figure S70: ^{1}HNMR spectrum (DMSO-d_6, 600 MHz) of compound **29**

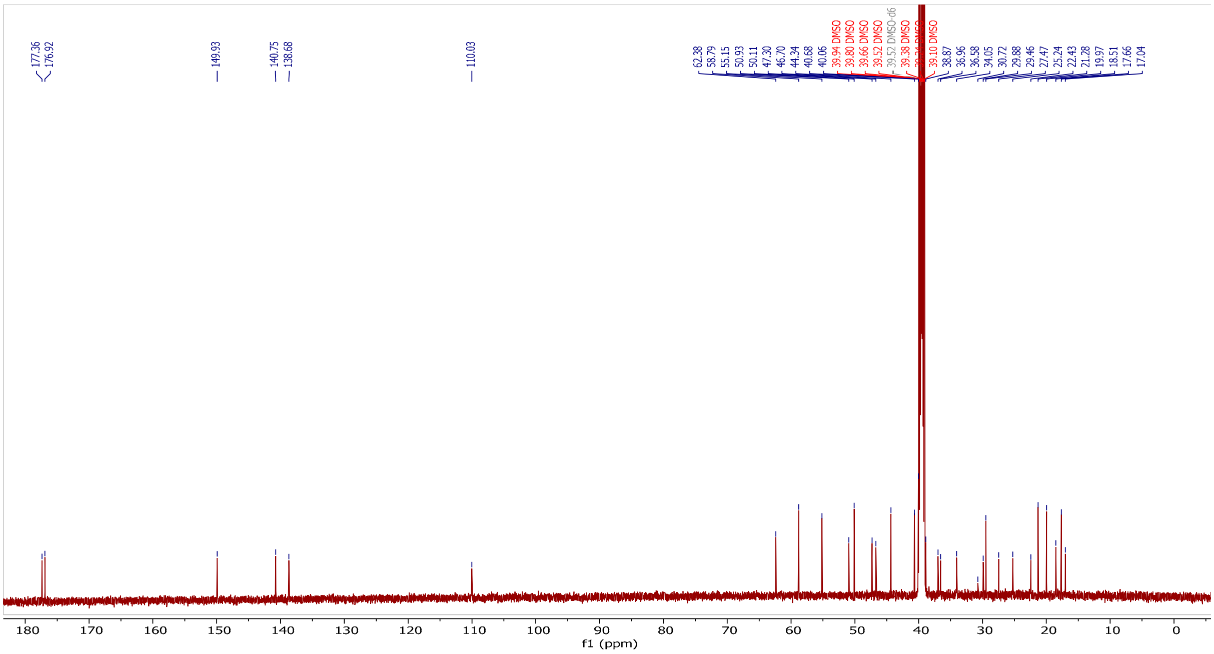

Figure S71: ^{13}CNMR spectrum of (DMSO-d_6, 150 MHz) of compound **29**

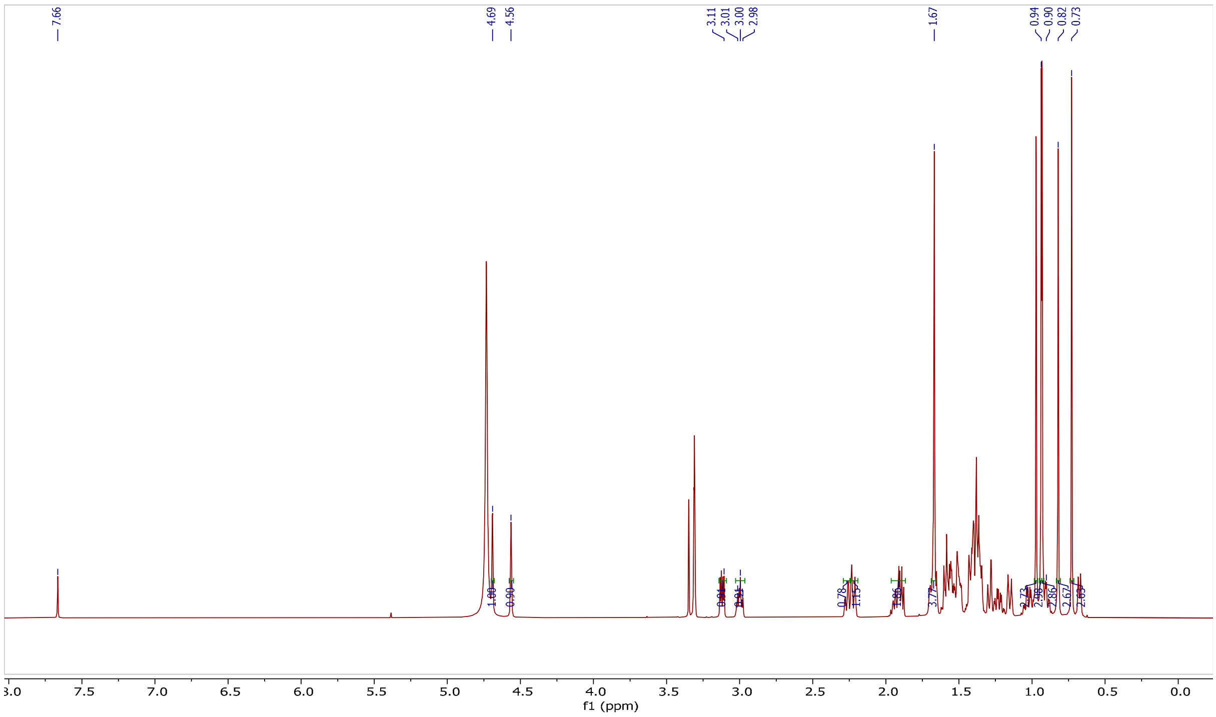

Figure S72: ^{1}H NMR spectrum ($CDCl_3$ / CD_3OD, 600 MHz) of **30**

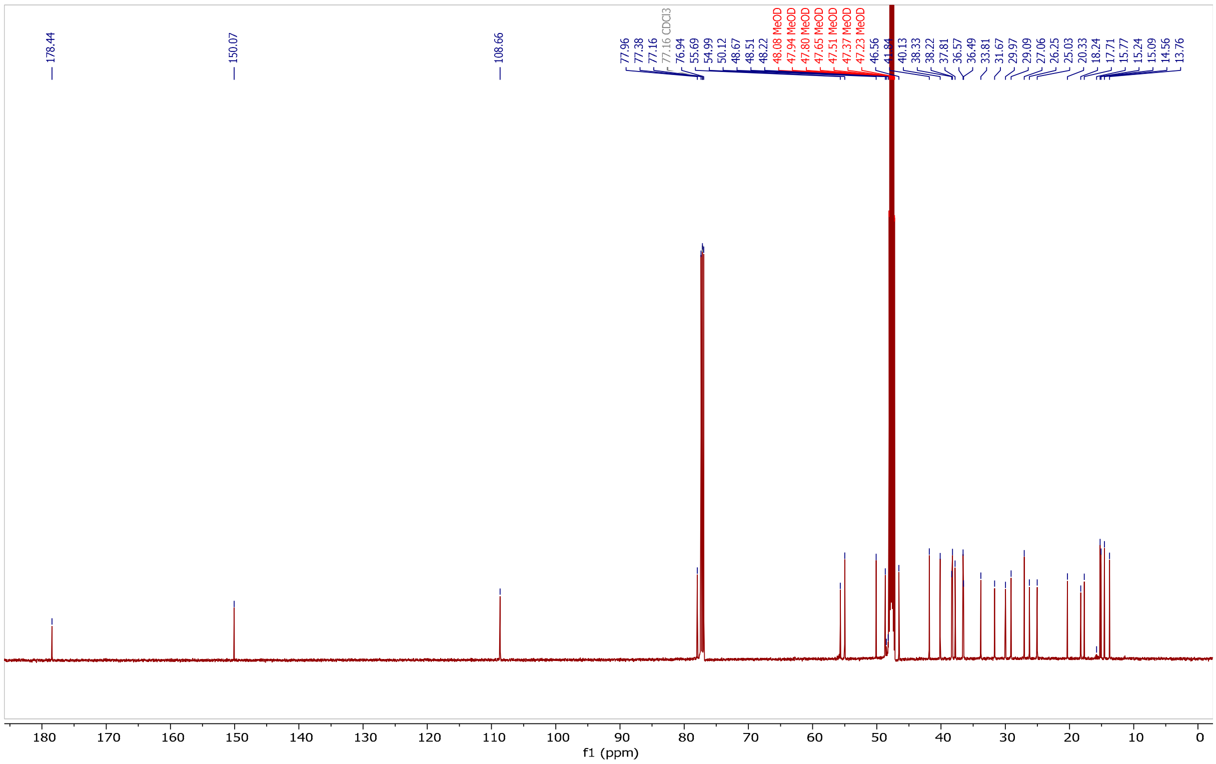

Figure S73: ^{13}C NMR spectrum ($CDCl_3$ / CD_3OD, 150 MHz) of compound **30**

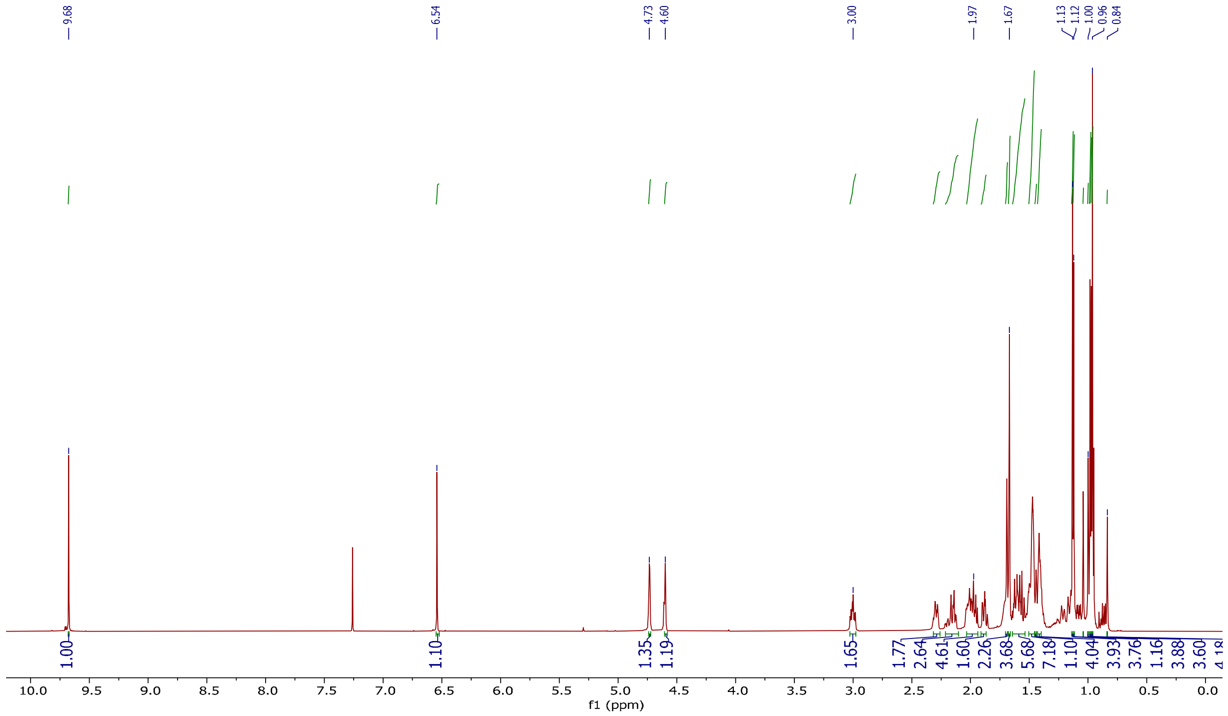

Figure S74: ^{1}H NMR spectrum ($CDCl_3$, 600 MHz) of compound **32**

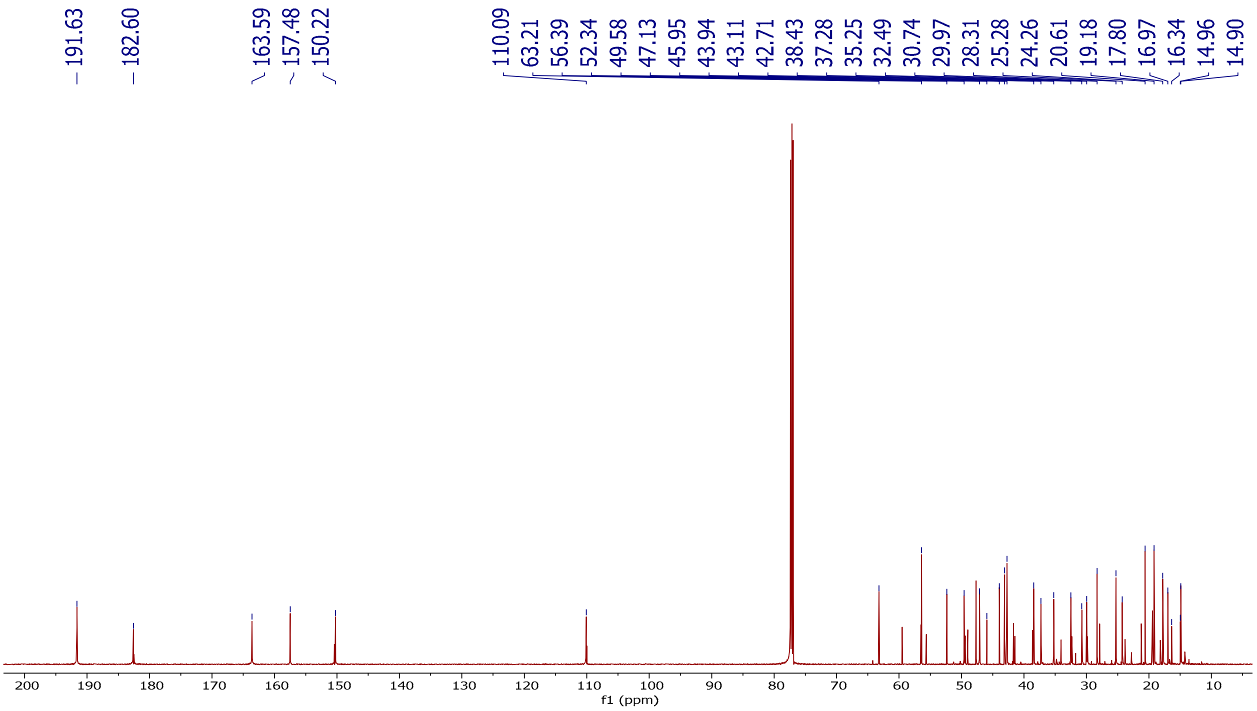

Figure S75: ^{13}C NMR spectrum ($CDCl_3$, 150 MHz) of compound **32**

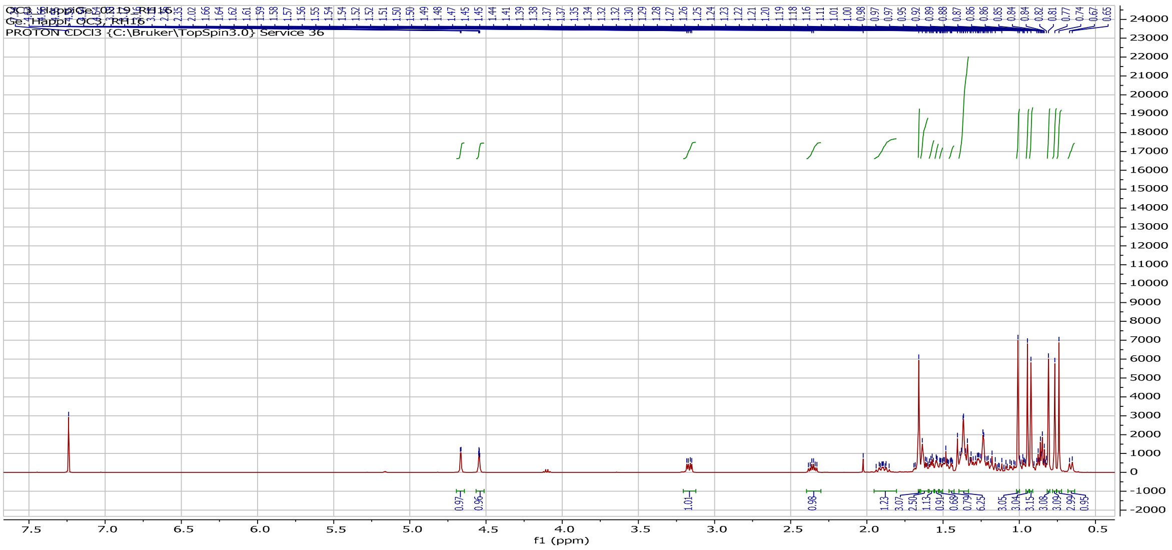

Figure S76: ^{1}H NMR spectrum ($CDCl_3$, 500 MHz) of compound **33**

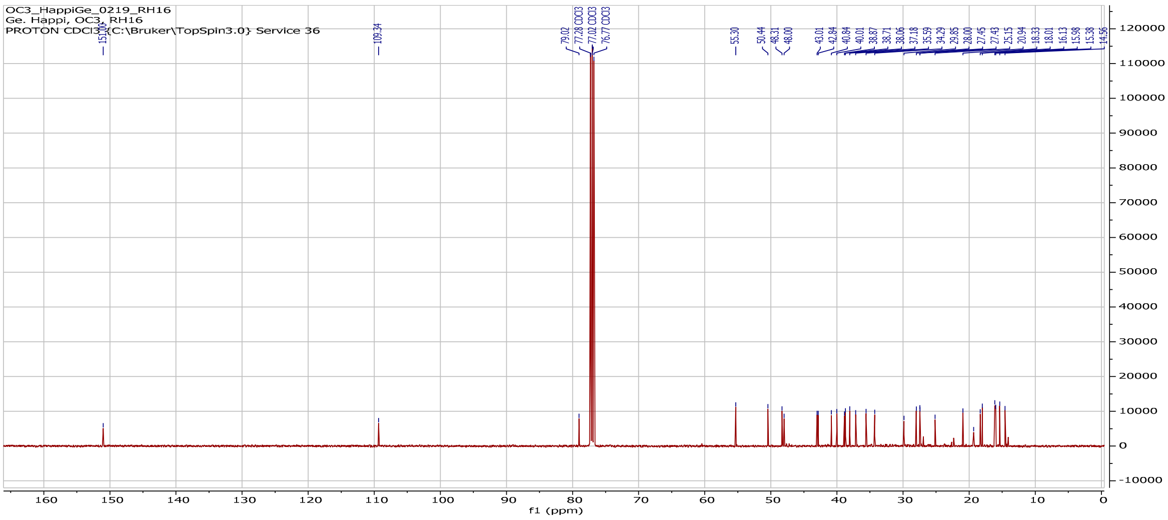

Figure S77: ^{13}C NMR spectrum ($CDCl_3$, 125 MHz) of compound **33**

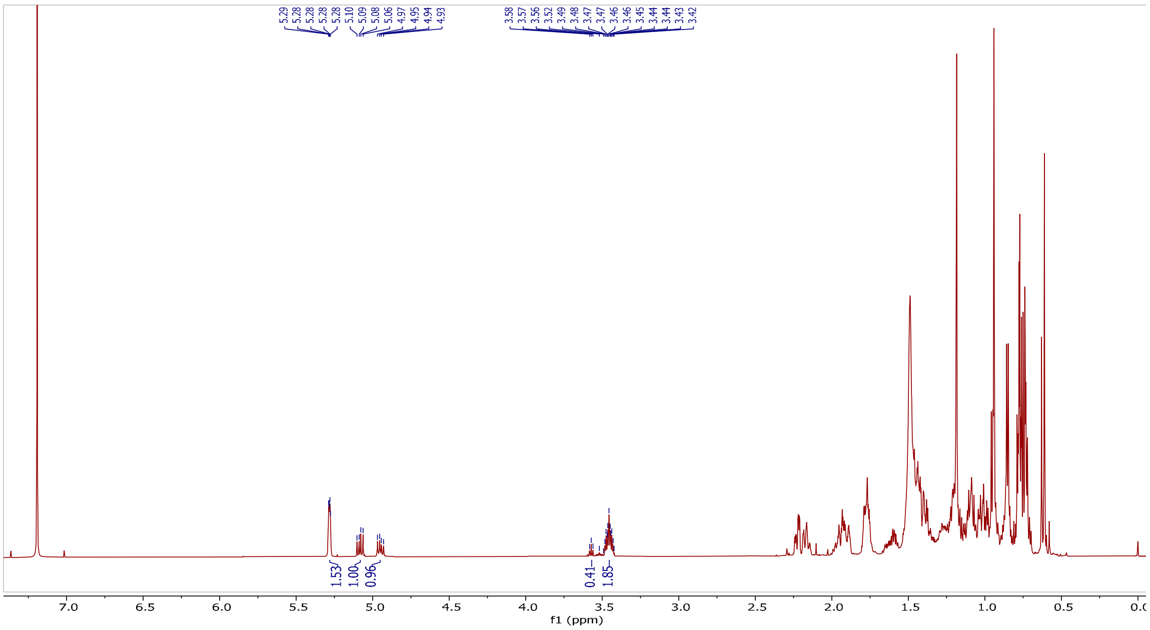

Figure S78: ^{1}H NMR spectrum ($CDCl_3$, 600 MHz) of compound **34** and **35**

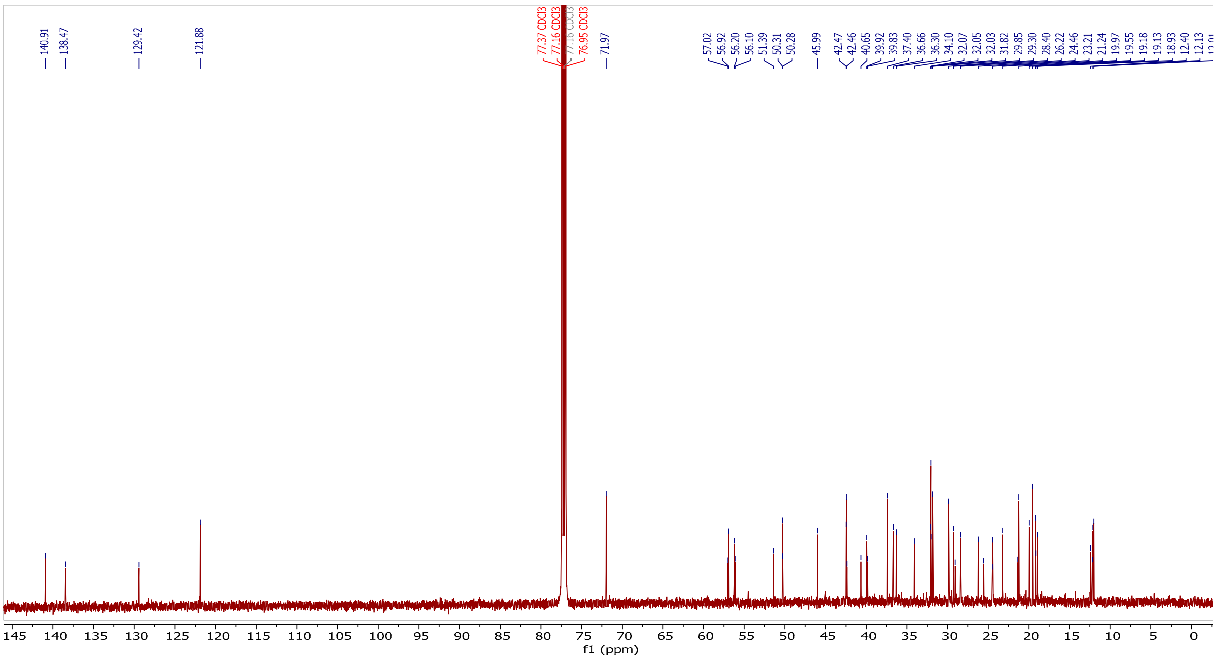

Figure S79: ^{13}C NMR spectrum ($CDCl_3$, 600 MHz) of compound **34** and **35**

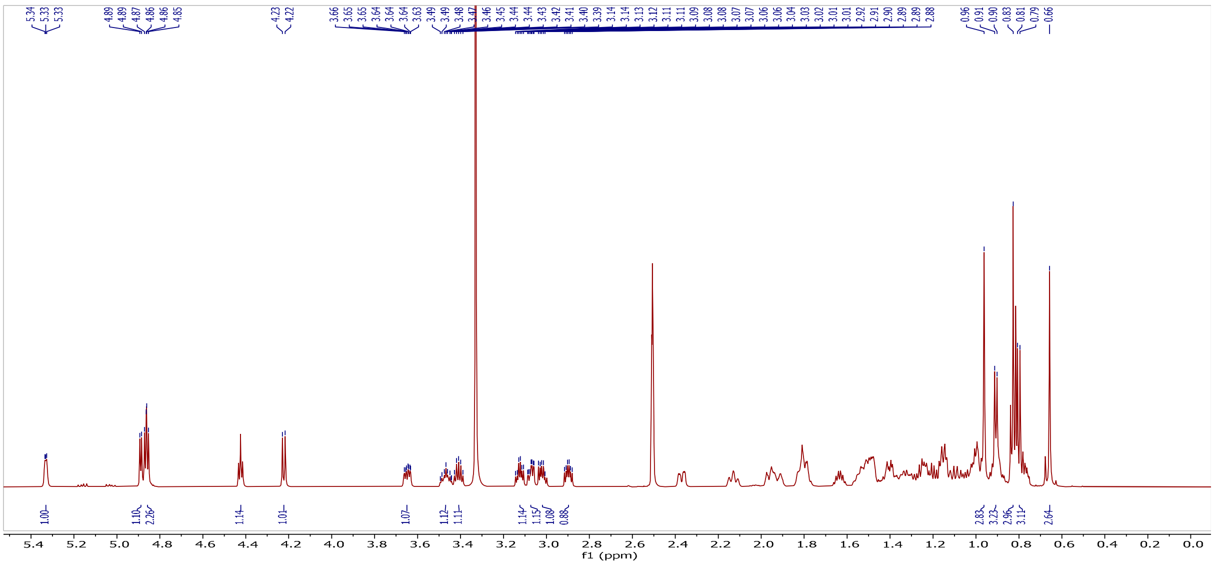

Figure S80: ^{1}HNMR spectrum (DMSO-d_6, 600 MHz) of compound **36**

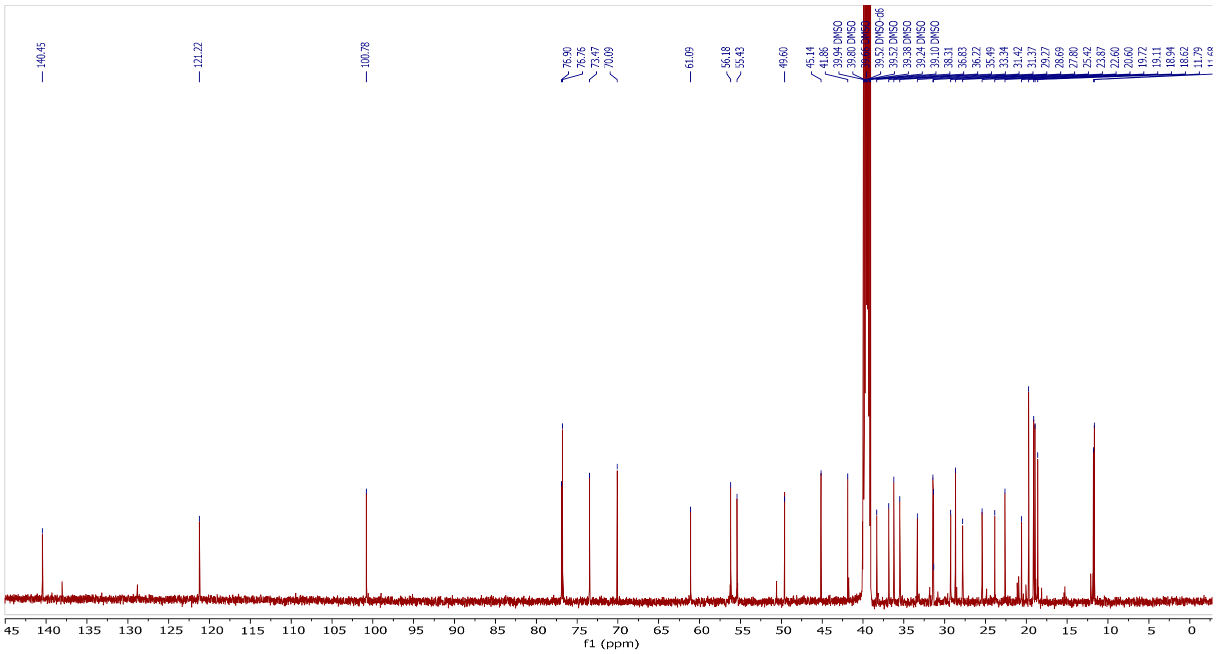

Figure S81: ^{13}CNMR spectrum (DMSO-d_6, 600 MHz) of compound **36**

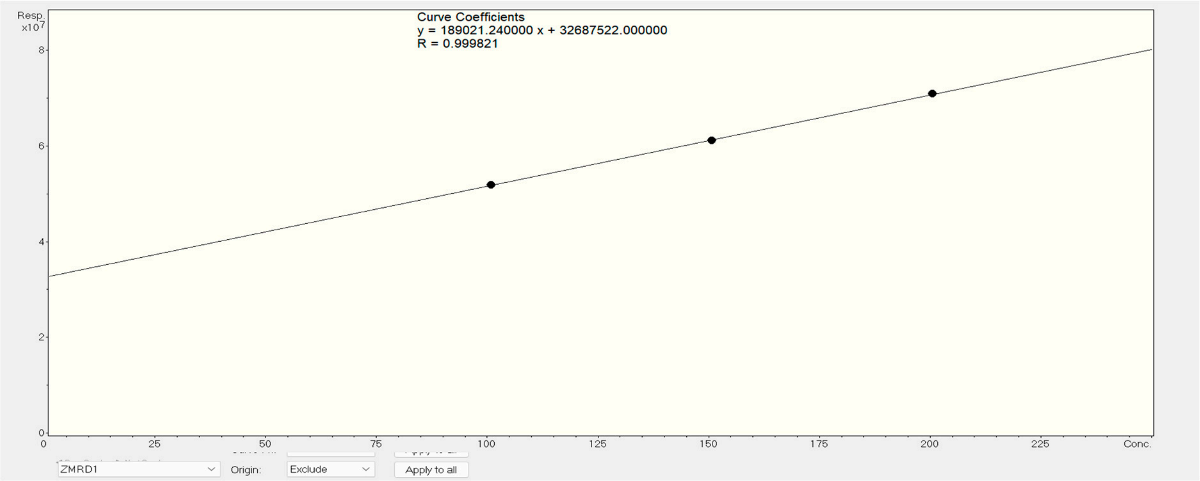

Figure S82: Calibration curve of mauritine A (**5**) using BPC

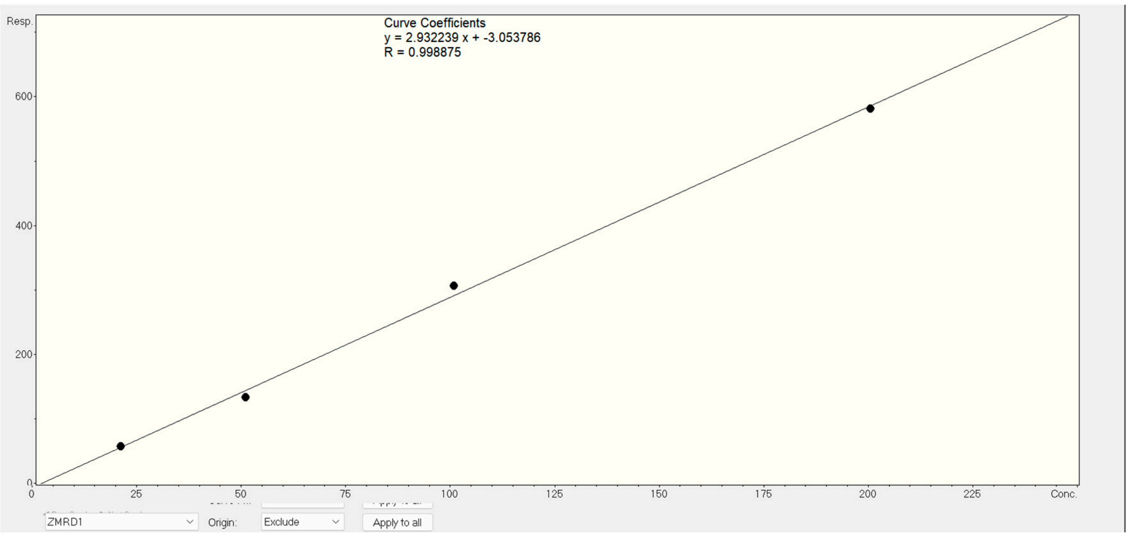

Figure S83: Calibration curve of mauritine A (**5**) using UV (λ=254 nM)

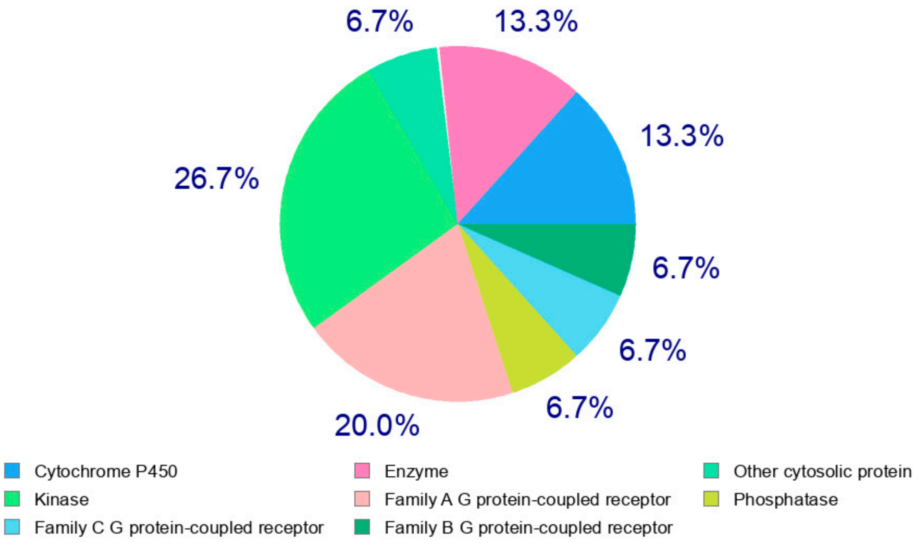

Figure S84: Pie chart of molecular targets of Artemisinin

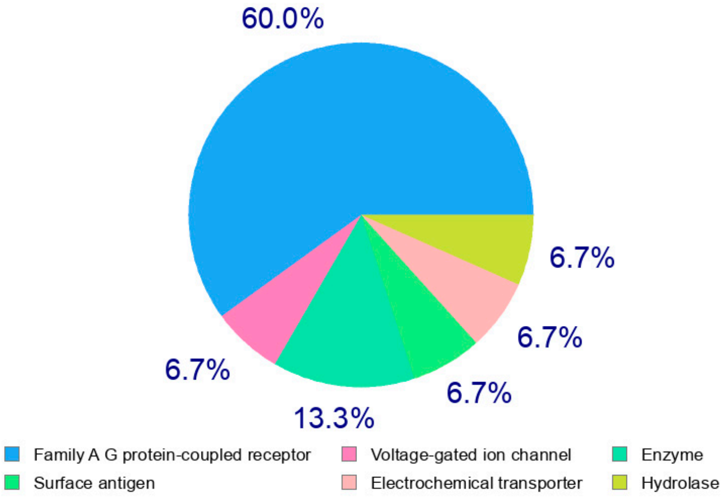

Figure S85: Pie chart of molecular targets of Chloroquine

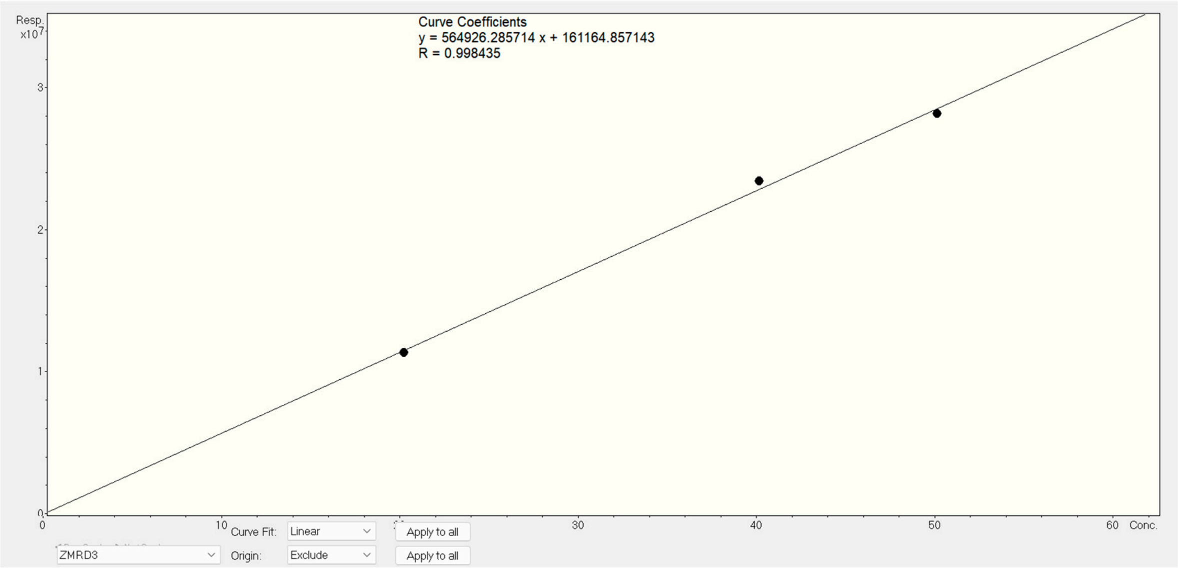

Figure S86: Calibration curve of amphibine A (**14**) using EIC
